# Scaffold Hopping Strategy toward New 4‑Aminoquinazolines Active Against Extracellular and Intracellular *Mycobacterium tuberculosis*

**DOI:** 10.1021/acsmedchemlett.5c00276

**Published:** 2025-06-30

**Authors:** Guilherme Arraché Gonçalves, Alexia de Matos Czeczot, Marcia Alberton Perelló, Eric Greve, Renee Allen, Camili Zanella Zotti, Laura Calle González, Andresa Berger, Josiane Delgado Paz, Lídia Klatt Oliveira, Sidnei Moura e Silva, Cristiano Valim Bizarro, Luiz Augusto Basso, Tanya Parish, Pablo Machado

**Affiliations:** † Instituto Nacional de Ciência e Tecnologia em Tuberculose, Centro de Pesquisas em Biologia Molecular e Funcional, 28102 Pontifícia Universidade Católica do Rio Grande do Sul, 90616-900 Porto Alegre, Rio Grande do Sul, Brazil; ‡ Programa de Pós-Graduação em Medicina e Ciências da Saúde, Pontifícia Universidade Católica do Rio Grande do Sul, 90616-900 Porto Alegre, Rio Grande do Sul, Brazil; § Center for Global Infectious Disease Research, 145793 Seattle Children’s Research Institute, Seattle, Washington 98101, United States of America; ∥ Programa de Pós-Graduação em Biologia Celular e Molecular, Pontifícia Universidade Católica do Rio Grande do Sul, 90616-900 Porto Alegre, Rio Grande do Sul, Brazil; ⊥ Laboratório de Biotecnologia de Produtos Naturais e Sintéticos, Universidade de Caxias do Sul, 95070-560 Caxias do Sul, Rio Grande do Sul, Brazil; # Department of Pediatrics, University of Washington School of Medicine, Seattle, Washington 98109, United States of America

**Keywords:** *Mycobacterium tuberculosis*, Drug design, Structure−activity relationship, Intracellular activity, Quinazolines

## Abstract

A series of 4-aminoquinazolines was designed through a scaffold hopping approach inspired by pharmacophoric features of known antimycobacterial agents. The compounds were synthesized via a one-pot silylation–amination reaction under solvent-free conditions, affording the desired molecules in 70%–99% yields. Antimycobacterial evaluation using multiple *Mycobacterium tuberculosis* strains and assay platforms revealed potent activity, with MIC values as low as 0.28 μM. Structure–activity relationship analysis identified the *N*-(3-phenylpropyl)­quinazolin-4-amine scaffold as a promising chemotype. Mechanistic studies indicated that the compounds do not act via QcrB inhibition, membrane disruption, ROS induction, or MmpL3 targeting. The most active derivatives displayed favorable selectivity indices, lacked broad-spectrum antibacterial activity, and demonstrated intracellular efficacy in a macrophage infection model. Despite low metabolic stability, the scaffold’s potency, selectivity, and intracellular activity support its potential as a lead series. These findings suggest a novel, yet unidentified mechanism of action and provide a promising starting point for anti-TB drug campaigns.

## Graphical abstract

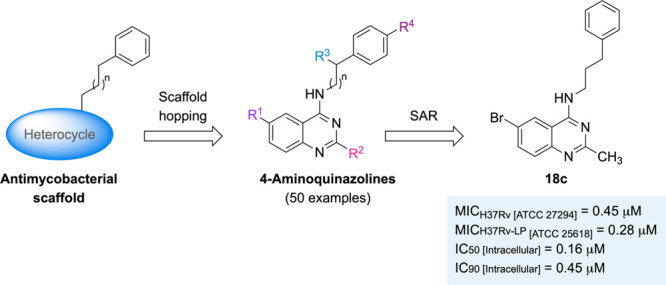

Tuberculosis (TB) has been recognized as a global health problem since the 1990s. Despite being preventable, treatable, and curable, TB continues to impact millions of people worldwide every year. The latest Global TB Report indicated that 10.8 million people developed TB in 2023, with 1.25 million deaths attributed to the disease.^1^ TB remains a persistent global health challenge, driven by a complex interplay of socioeconomic determinants (e.g., poverty) and risk factors that contribute to disease progression, such as undernutrition, alcohol use disorders, smoking, HIV infection, and diabetes.^1,2^ The biology and pathogenesis of the etiological agent, *Mycobacterium tuberculosis*, also plays a role in the complexity of the disease.^1–3^

Approximately one-quarter of the global population is estimated to be latently infected with *M. tuberculosis*.^1^ This asymptomatic, nontransmissible state is characterized by dormant bacilli, which can reactivate and progress to active TB, particularly in immunocompromised individuals. Mycobacteria primarily establish infection in the lungs, leading to the formation of tuberculous granulomas, which serve as a niche for bacterial persistence.^2,3^ Within these granulomas, heterogeneous bacillary populations emerge, exhibiting varying metabolic states and drug susceptibilities.^3,4^ This dynamic and complex environment presents a significant challenge to antimicrobial therapy often requiring prolonged multidrug treatment to ensure complete bacterial eradication and prevent disease relapse.^1,5^

The first-line treatment comprises four drugs (isoniazid, rifampicin, ethambutol, and pyrazinamide) and requires at least 6 months of therapy with strict adherence. Patient compliance and treatment discontinuation are some of the main challenges in TB chemotherapy, potentially leading to the emergence of drug-resistant strains.^1,5,6^ Several strains resistant to isoniazid and rifampicin have already been identified, including those resistant to newer drugs such as bedaquiline and delamanid.^6,7^ Thus, there is a pressing need to develop novel drugs that, ideally, act through mechanisms distinct from current TB treatments.

Aligned with our research program, we aim to develop and explore novel scaffolds with antitubercular potential. Since the approval of bedaquiline, an ATP synthase inhibitor, the mycobacterial oxidative phosphorylation pathway has gained substantial attention over the past decade.^8,9^ Within this context, we have contributed to the field by designing and synthesizing compounds that inhibit bacterial viability by targeting the cytochrome *bc*_1_ complex.^10–14^ This target has attracted significant attention, with reports describing diverse chemical classes that inhibit *M. tuberculosis* growth by specifically targeting the QcrB subunit of the cytochrome *bc*_1_ complex.^9^ These classes include triazolopyrimidines **1** (TAP),^13^ benzimidazoles **2** (FABA),^14^ and thieno­[2,3-*d*]­pyrimidines **3** (CWHM-1023)^15^ (Figure 1A). Interestingly, from a chemical perspective, these compounds share three main structural similarities, as represented in the general structure **4**: (a) a heterocycle; (b) a linker of variable length; and (c) a benzene moiety at the terminal portion of the linker (Figure 1B). Furthermore, a substantial body of research has reported that quinazoline-based compounds exhibit antitubercular activity.^16–20^ Importantly, some studies have reported that certain quinazoline derivatives can interfere with the bacillus’s energy metabolism.^21–24^

**Figure 1:**
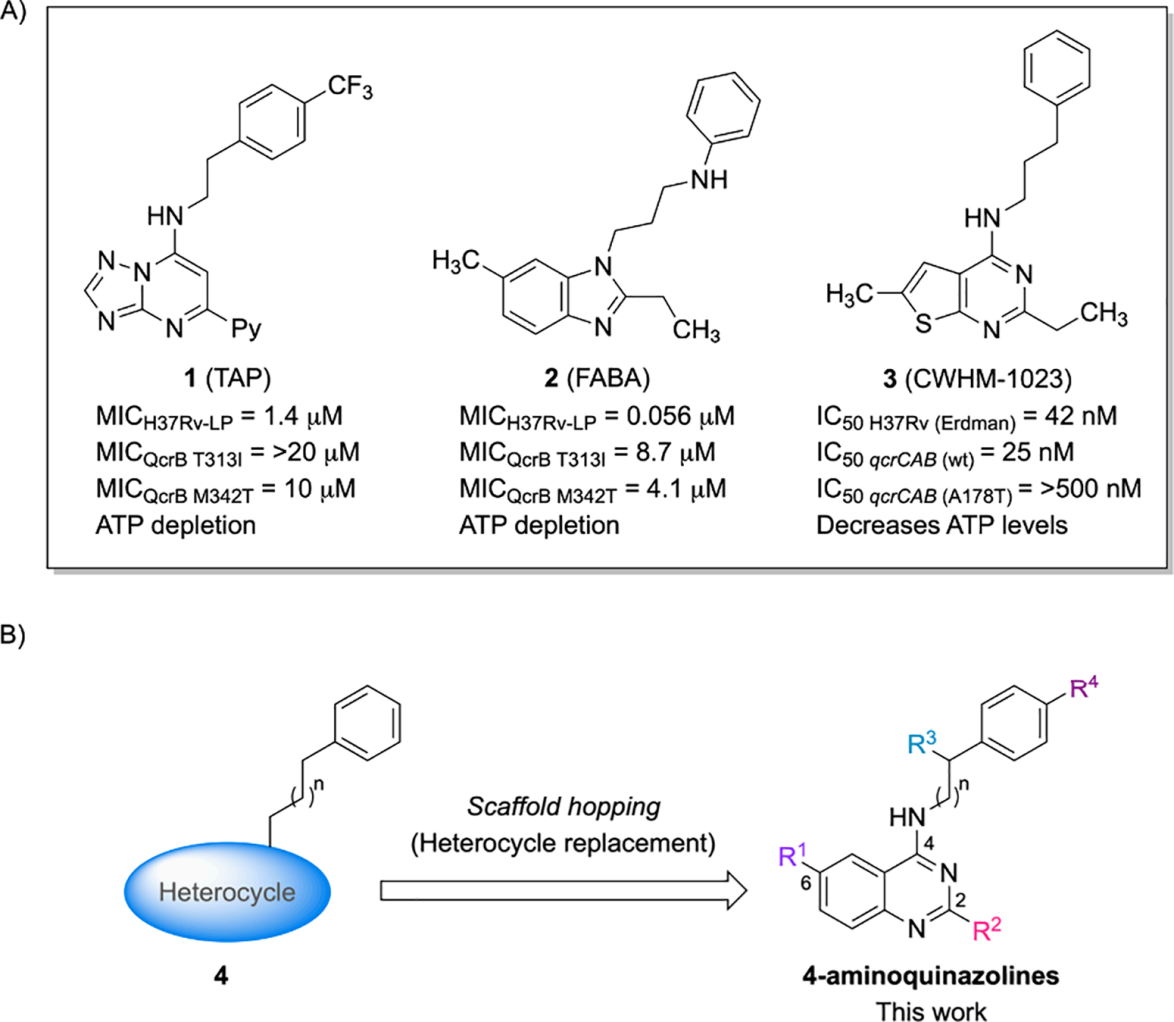
(A) *M. tuberculosis* QcrB inhibitors; (B) Pharmacophoric features of *M. tuberculosis* growth inhibitors represented in general structure **4**, which guided the design of the investigated 4-aminoquinazolines.

Therefore, considering the pharmacophoric features of the general structure **4** and the presence of quinazoline scaffolds in molecules with potent antimycobacterial activity, a classical scaffold hopping strategy was proposed. The new compounds were designed by incorporating a 4-aminoquinazoline core into structure **4** (Figure 1B), while retaining the original linker and benzene moiety. Notably, scaffold hopping has been frequently employed in early stage drug discovery, where known active chemical structures serve as starting points to generate new analogues that preserve the biological profile of the parent structure.^25,26^

Based on this rationale, herein, a series of 4-aminoquinazolines was synthesized and initially evaluated against *M. tuberculosis* H37Rv. The most promising compounds were selected for further evaluation using an absorbance/fluorescence-based assay with the *M. tuberculosis* H37Rv-LP strain. Mechanistic studies were conducted to explore potential modes of action, including QcrB inhibition, membrane disruption, ROS induction, and MmpL3 targeting. In parallel, HepG2 and Vero cell viability assays were performed to provide insights into selectivity and cytotoxicity. Metabolic stability in liver microsomes was also assessed to inform druglike properties. Finally, the intracellular antimycobacterial activity of selected molecules was investigated using a macrophage infection model, aiming to validate their potential efficacy in the intracellular environment characteristic of tuberculosis infection.

The synthesis of the target 4-aminoquinazolines was accomplished in two steps (Scheme 1). In the first step, the Niementowski reaction was employed to generate key quinazolinone intermediates **7a**–**7d**.^27^ Specifically, anthranilic acids **5a**–**5d** (R^1^ = H, OCH_3_, Br, I) were reacted with thioacetamide **6** in a 1:1.5 molar ratio at 150 °C, affording 2-methylquinazolin-4­(3*H*)-ones **7a**–**7d** in 52%–76% yields. The reaction mixtures solidified after heating, and yields were primarily influenced by crystallization efficiency, as the starting anthranilic acids were fully consumed. Compound **7e** was commercially sourced and used directly in the next step without purification. Substitution patterns at the 2- (R^2^ = H, CH_3_) and 6-positions (R^1^ = H, OCH_3_, Br, I) were chosen based on electronic and structural criteria, incorporating electron-donating (EDG) and electron-withdrawing groups (EWG) to further explore their influence on antimycobacterial activity.

**Scheme 1:**
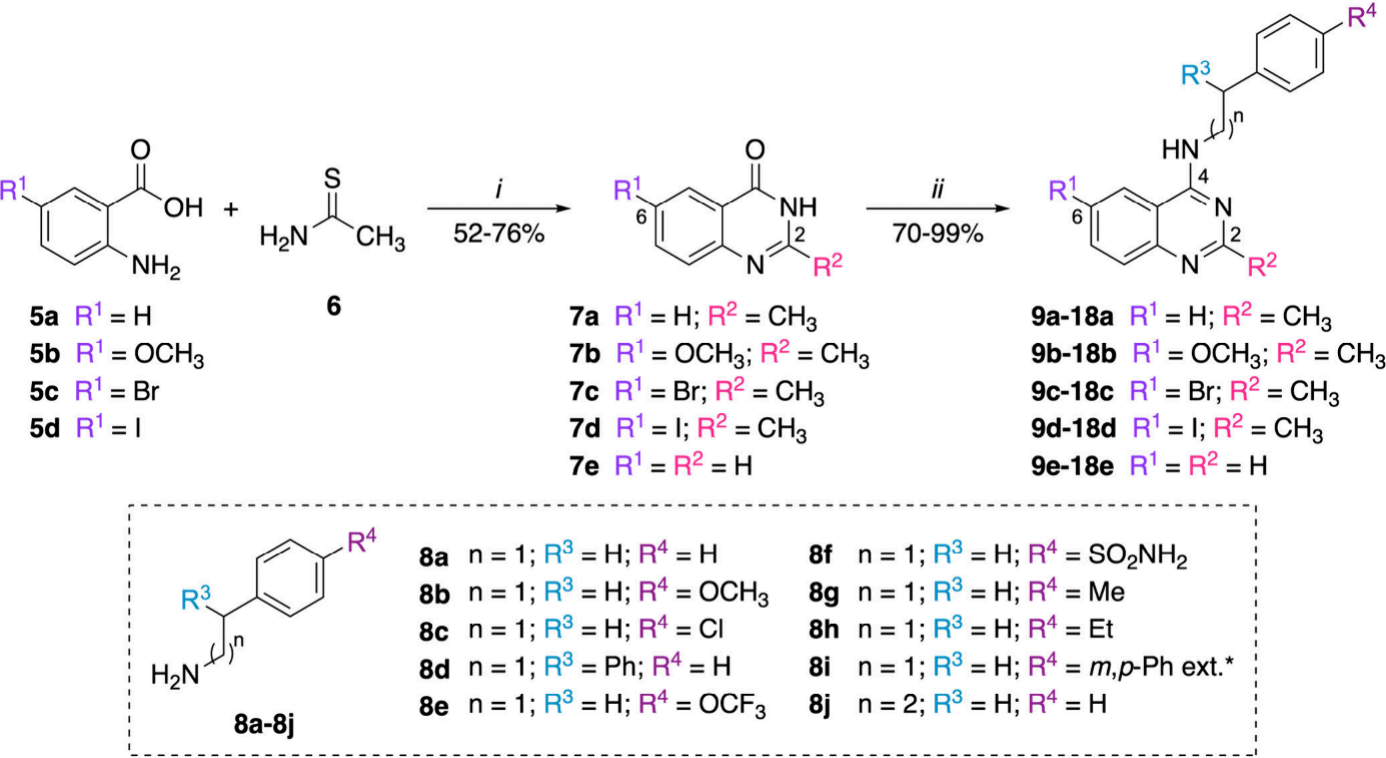
Synthesis of 4-Aminoquinazolines^a^

a Conditions and reagents are as follows: (i) *T* = 150 °C, 2 h, 52–76%. (ii): **8a**–**8j**, HMDS, (NH_4_)_2_SO_4_, *T* = 125 °C, 24–48 h, 70%–99%. *8i: 2-(naphthalen-2-yl)­ethan-1-amine.

In the second reaction step, a silylation–amination reaction mediated by hexamethyldisilazane (HMDS) was used to obtain the final 4-aminoquinazolines **9a**–**18e**, with 70%–99% yields The protocol, previously reported by our group,^28^ employed a 1:3:3 molar ratio of quinazolinone, amine, and HMDS under solvent-free conditions at 125 °C, in the presence of ammonium sulfate ((NH_4_)_2_SO_4_, 10 mol %) as a Lewis acid catalyst. For amine **8i**, the HMDS ratio was increased to 1:3:4 to ensure solubilization. This one-pot process avoided the need for chlorination and was compatible with a broad range of functional groups, including EDGs, EWGs, and bulky moieties. Product yields were more dependent on purification efficiency than on the electronic or steric nature of the substituents. Structural variations were introduced at R^3^ and R^4^ (on the amine moiety), R^1^ and R^2^ (on the quinazoline core), and via linker elongation (*n* = 1 or 2). Specifically, the 4-position was diversified using phenethylamines (**8a**–**8i**, *n* = 1) and the homologous 3-phenylpropan-1-amine (**8j**, *n* = 2), providing insights into the influence of linker extension on antimycobacterial activity. For reaction times and yields corresponding to each substituent, see the Supporting Information.

The minimum inhibitory concentrations (MICs) of the 4-aminoquinazolines **9a**–**18e** were determined using two *M. tuberculosis* H37Rv strains. Initially, the entire series was screened against the H37Rv strain (ATCC 27294) using the resazurin reduction microplate assay (REMA).^29^ The synthesized compounds exhibited MIC values ranging from 0.45 to >121.8 μM (Table 1), highlighting a range of antimycobacterial potency within the series. Relevant structure–activity relationship (SAR) insights were obtained for the series (Figure 2A). For clarity, the compounds were grouped into five subseries, based on substituents on the quinazoline core: (a) **9a**–**18a** (R^1^ = H; R^2^ = CH_3_); (b) **9b**–**18b** (R^1^ = OCH_3_; R^2^ = CH_3_); (c) **9c**–**18c** (R^1^ = Br; R^2^ = CH_3_); (d) **9d**–**18d** (R^1^ = I; R^2^ = CH_3_); and (e) **9e**–**18e** (R^1^ = R^2^ = H). Substituents on the heterocyclic ring were shown to significantly influence antimycobacterial activity. Molecules from subseries **9e**–**18e**, lacking substitution on the quinazoline ring, showed the highest MIC values, indicating low activity. In contrast, introducing a methyl group at 2-position improved potency. For instance, **13a** (R^4^ = OCF_3_) was 8-fold more active than **13e** (MIC = 7.2 vs 60.0 μM). Substitution at 6-position followed the trend I > Br > OCH_3_ ≥ H. Methoxy had limited effect, as seen in the similar activity of subseries **9a**–**18a** and **9b**–**18b**. Halogenation at R^1^ led to more active compounds; notably, **9d** (MIC = 3.2 μM) was 12-fold more potent than **9a**.

**Table 1:**
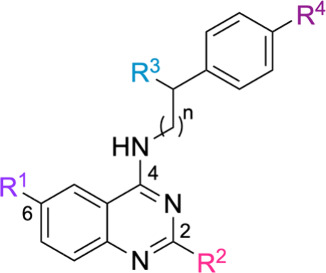
In Vitro Activity of 4-Aminoquinazolines against *M. tuberculosis* H37Rv (ATCC 27294)

entry	*n*	R^1^	R^2^	R^3^	R^4^	MIC^a^ (μM)	*c* log *P* ^b^
**9a**	1	H	CH_3_	H	H	38.​0	2.​62
**10a**	1	H	CH_3_	H	OCH_3_	34.​1	2.​54
**11a**	1	H	CH_3_	H	Cl	16.​8	3.​34
**12a**	1	H	CH_3_	Ph	H	29.​5	4.​06
**13a**	1	H	CH_3_	H	OCF_3_	7.​2	3.​65
**14a**	1	H	CH_3_	H	SO_2_NH_2_	>116.​8^c^	0.​79^d^
**15a**	1	H	CH_3_	H	Me	18.​0	3.​12
**16a**	1	H	CH_3_	H	Et	17.​2	3.​65
**17a**	1	H	CH_3_	H	*m*,*p*-Ph ext.	8.​0	3.​80
**18a**	2	H	CH_3_	H	H	4.​5	3.​00
**9b**	1	OCH_3_	CH_3_	H	H	34.​1	2.​54
**10b**	1	OCH_3_	CH_3_	H	OCH_3_	30.​9	2.​46
**11b**	1	OCH_3_	CH_3_	H	Cl	30.​5	3.​26
**12b**	1	OCH_3_	CH_3_	Ph	H	27.​1	3.​98
**13b**	1	OCH_3_	CH_3_	H	OCF_3_	13.​3	3.​57
**14b**	1	OCH_3_	CH_3_	H	SO_2_NH_2_	>107.​4^c^	0.​71^d^
**15b**	1	OCH_3_	CH_3_	H	Me	16.​3	3.​04
**16b**	1	OCH_3_	CH_3_	H	Et	15.​6	3.​57
**17b**	1	OCH_3_	CH_3_	H	*m*,*p*-Ph ext.	7.​3	3.​72
**18b**	2	OCH_3_	CH_3_	H	H	4.​1	2.​92
**9c**	1	Br	CH_3_	H	H	7.​3	3.​49
**10c**	1	Br	CH_3_	H	OCH_3_	13.​4	3.​41
**11c**	1	Br	CH_3_	H	Cl	13.​3	4.​20
**12c**	1	Br	CH_3_	Ph	H	>23.​9^c^	4.​92^d^
**13c**	1	Br	CH_3_	H	OCF_3_	5.​9	4.​51
**14c**	1	Br	CH_3_	H	SO_2_NH_2_	>94.​9^c^	1.​65^d^
**15c**	1	Br	CH_3_	H	Me	7.​0	3.​99
**16c**	1	Br	CH_3_	H	Et	6.​8	4.​51
**17c**	1	Br	CH_3_	H	*m*,*p*-Ph ext.	3.​2	4.​66
**18c**	2	Br	CH_3_	H	H	0.​45	3.​87
**9d**	1	I	CH_3_	H	H	3.​2	3.​75
**10d**	1	I	CH_3_	H	OCH_3_	1.​5	3.​67
**11d**	1	I	CH_3_	H	Cl	0.​73	4.​46
**12d**	1	I	CH_3_	Ph	H	>21.​5^c^	5.​18^d^
**13d**	1	I	CH_3_	H	OCF_3_	5.​3	4.​77
**14d**	1	I	CH_3_	H	SO_2_NH_2_	>85.​4^c^	1.​91^d^
**15d**	1	I	CH_3_	H	Me	3.​1	4.​25
**16d**	1	I	CH_3_	H	Et	6.​0	4.​77
**17d**	1	I	CH_3_	H	*m*,*p*-Ph ext.	1.​4	4.​92
**18d**	2	I	CH_3_	H	H	1.​6	4.​13
**9e**	1	H	H	H	H	80.​2	2.​12
**10e**	1	H	H	H	OCH_3_	71.​6	2.​04
**11e**	1	H	H	H	Cl	>35.​2^c^	2.​84^d^
**12e**	1	H	H	Ph	H	>30.​7^c^	3.​56^d^
**13e**	1	H	H	H	OCF_3_	60.​0	3.​15
**14e**	1	H	H	H	SO_2_NH_2_	>121.​8^c^	0.​30^d^
**15e**	1	H	H	H	Me	>38.​0^c^	2.​62^d^
**16e**	1	H	H	H	Et	36.​0	3.​15
**17e**	1	H	H	H	*m*,*p*-Ph ext.	>16.​7^c^	3.​30^d^
**18e**	2	H	H	H	H	19.​0	2.​50
**19 (INH)**	–	–	–	–	–	2.​3	–​
**20 (RIF)**	–	–	–	–	–	0.​20	–​

a The values obtained from the assay were converted from μg/mL to μM.

b *c* log *P* determined using ChemDraw software (version: 20.0.0.38).

c The symbol “>” means that the substance has a MIC above the concentrations tested.

d Substances removed from the MIC × *c* log *P* nonlinear regression analysis due to lack of activity at the tested concentrations. *m*,*p*-Ph ext., 2-naphthyl group. INH, isoniazid. RIF, rifampicin.

**Figure 2:**
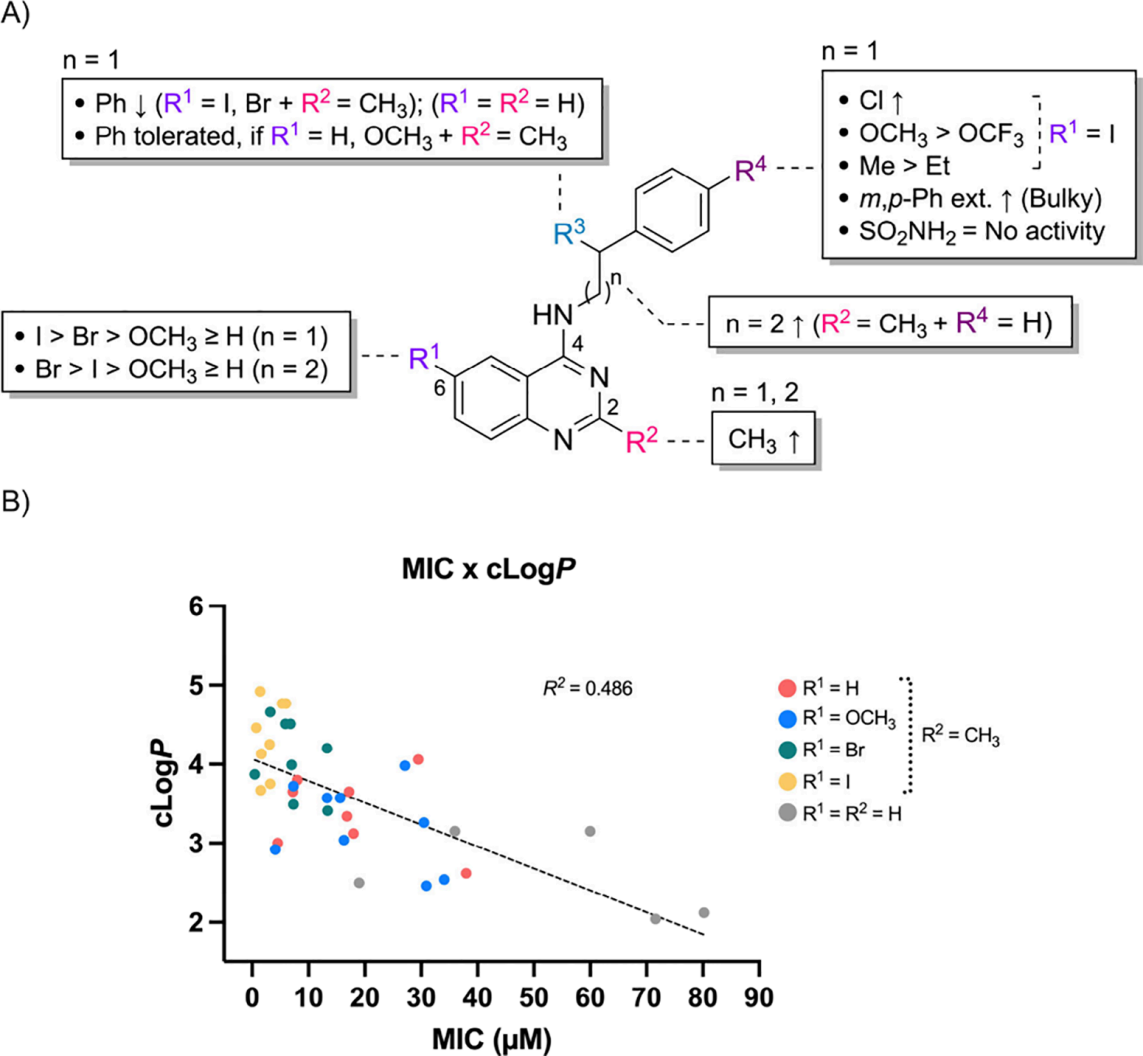
(A) Structure–activity relationship of the 4-aminoquinazoline series. (B) Correlation between MIC values and *c* log *P* for the 4-aminoquinazoline series. Nonlinear regression was performed using GraphPad Prism 10 (Version 10.1.1), yielding a *Y*-intercept of 4.065, a slope of −0.0276, and an *R* ^*2*^ of 0.486 (*n* = 39).

A correlation between *c* log *P* and MIC was observed (Figure 2B), reflecting SAR trends: I > Br > OCH_3_ ≥ H (R^1^) and CH_3_ > H (R^2^). The least active subseries (**9e**–**18e**) clustered in the lower midright region of the plot, while **9a**–**18a** and **9b**–**18b** occupied an intermediate zone. Structures from subseries **9c**–**18c** and **9d**–**18d**, which had higher *c* log *P* values, were grouped in the upper midleft region and represented the most potent molecules. However, while increased lipophilicity generally favored activity, this relationship was not strictly linear within each subseries. The moderate *R* ^*2*^ value (0.486) suggests that *c* log *P* contributes to activity, but other molecular properties are also likely involved. These may include molecular volume, steric hindrance, rigidity, solubility, permeability, and electronic effects. Within this context, comparison between 4-aminoquinazolines bearing phenethylamines (**8a**–**8i**, *n* = 1) and those with an extended linker (**8j**, *n* = 2) further enriched the SAR analysis.

Within each subseries, the R^4^ substituent influenced antimycobacterial activity to varying degrees. While some modifications had minimal impact, compared to the unsubstituted compounds (**9a–9e**, R^4^ = H), others led to notable improvements in potency. For instance, derivatives bearing an OCF_3_ group at R^4^—such as **13a–13c** and **13e**—displayed enhanced activity (MIC = 5.9–60.0 μM) relative to their unsubstituted (**9a–9c**, **9e**; MIC = 7.3–80.2 μM) and OCH_3_-substituted counterparts (**10a–10c**, **10e**; MIC = 13.4–71.6 μM). However, this trend did not extend to iodinated analogues, as **9d** (R^4^ = H) and **10d** (R^4^ = OCH_3_) were more potent (MIC = 3.2 and 1.5 μM, respectively) than the corresponding OCF_3_-containing compound **13d** (MIC = 5.3 μM). Apart from **10d**, OCH_3_ substitution had limited impact across the series. Furthermore, alkyl substituents at R^4^ (Me, Et) produced MIC values (from 3.1 to >38.0 μM) comparable to those of OCF_3_ analogues, with methyl and ethyl derivatives exhibiting similar activity. In contrast, chlorine substitution at R^4^ yielded mixed results within the **11a–11e** series. Among them, **11d** (R^1^ = I; R^2^ = CH_3_) emerged as one of the most potent molecules (MIC = 0.73 μM), showing 23- and 18-fold activity gains over **11a** and **11c**, respectively. This highlights the favorable impact of iodine substitution at 6-position when combined with chlorine at R^4^. Conversely, sulfonamide substitution at R^4^ led to a complete loss of activity across **14a–14e** (MIC > 85.4 μM), suggesting that high polarity at this position compromises antimycobacterial efficacy.

In addition, steric factors also played a critical role. The introduction of a phenyl group at R^3^ significantly reduced activity in halogenated analogues **12c** and **12d** (MIC > 23.9 and >21.5 μM), despite their high *c* log *P* values (4.92 and 5.18, respectively). These results reinforce the idea that lipophilicity alone does not ensure potency and that excessive bulk at the 4-substituent may interfere with a putative target binding. Although the phenyl group was tolerated in nonhalogenated analogues **12a** and **12b**, no notable gains in activity were observed. By contrast, introducing a naphthyl group at R^4^ generally enhanced potency. This effect was particularly evident in halogenated structures **17c** (R^1^ = Br) and **17d** (R^1^ = I), which ranked among the most active derivatives (MIC = 3.2 and 1.4 μM, respectively). Nonhalogenated analogs **17a** and **17b** (R^1^ = H, OCH_3_) showed moderate activity (MIC = 8.0 and 7.3 μM), while **17e** (R^1^ = R^2^ = H) was inactive at the evaluated concentration (MIC > 16.7 μM). These results suggest that increased molecular volume at R^4^ may favor binding, potentially by improving accommodation of the terminal *N*-phenethyl chain.

Further SAR insights emerged from linker homologation. Extension of the alkyl chain at position 4 (*n* = 2) led to consistent improvements in activity across **18a–18e** (R^4^ = H), compared to their phenethyl analogues **9a–9e** (*n* = 1). While the enhancement was modest for **18d** (MIC = 1.6 μM), more pronounced gains were observed for **18a**, **18b**, and **18e** (MIC = 4.1–19.0 μM). Notably, 4-aminoquinazoline **18c** (R^1^ = Br) was the most potent of the series, with an MIC of 0.45 μM—approximately 10-fold more active than **18a** (MIC = 4.5 μM) and 16-fold more active than its phenethyl counterpart **9c** (MIC = 7.3 μM). Together, these results highlight that both chain elongation at the 4-position and specific quinazoline core substitutions (R^1^ = Br or I; R^2^ = CH_3_) are key structural determinants of enhanced antimycobacterial activity in this series.

Based on initial screening results, 15 4-aminoquinazolines were selected for further evaluation using a more sensitive and quantitative absorbance/fluorescence-based assay.^30^ For this purpose, the *M. tuberculosis* H37Rv-LP strain (ATCC 25618), which constitutively expresses codon-optimized DsRed,^31^ was employed. The selected compounds inhibited bacillary growth with MIC_90_ values ranging from 0.28 μM to 24 μM (Table 2). Given that these molecules were designed via scaffold hopping from known QcrB inhibitors, the cytochrome *bc*_1_ QcrB subunit was investigated as the primary target for this series. To evaluate this, the selected structures were tested against a strain of *M. tuberculosis* with the mutation QcrB_T313I_ (a residue critical for inhibitor binding at the Q_P_ site), and MIC values were compared to those obtained against the parental H37Rv-LP strain (Table 2). Overall, MIC values determined using the H37Rv-LP strain were consistent with those observed for H37Rv (ATCC 27294) via the REMA assay. In some cases, the H37Rv strain displayed slightly reduced susceptibility. When tested against the QcrB_T313I_ mutant strain a shift in MIC values would be expected if QcrB were the molecular target. However, no significant MIC differences were observed between the wild-type (H37Rv-LP) and mutant strains. These results suggest that the synthesized compounds do not inhibit QcrB at the Q_P_ site, the known binding region of several established inhibitors,^32,33^ including the clinical candidate Q203.^32,34^ This finding implies that the heterocycle replacement introduced via scaffold hopping likely disrupted essential features required for productive binding to QcrB. Afterward, ATP depletion assays were performed, as prior studies have shown that quinazoline derivatives may disrupt oxidative phosphorylation through alternative pathways.^21–24^ However, none of the selected molecules produced a significant ATP depletion profile, including the most potent derivative, **18c**, compared to the positive control Q203 (Supporting Information). Collectively, these results indicate that incorporation of the quinazoline scaffold into the structural pattern **4** (Figure 1B) did not preserve QcrB inhibition as the primary mode of action. Nevertheless, the potent antimycobacterial activity observed, along with clear SAR trends, supports the hypothesis that these compounds act through an alternative—and potentially novel—mechanism.

**Table 2:**
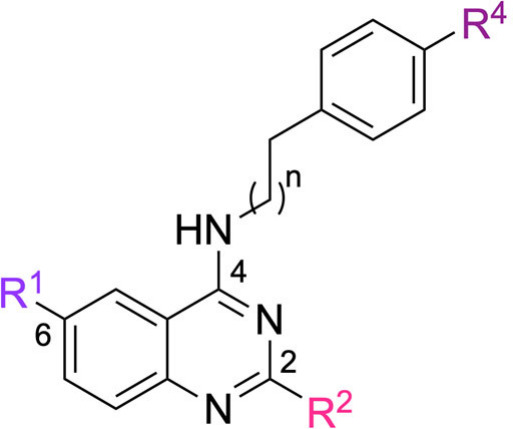
In Vitro Activity of Selected 4-Aminoquinazolines against *M. tuberculosis* H37Rv-LP (ATCC 25618) and QcrB_T313I_ Mutant

entry	*n*	R^1^	R^2^	R^4^	parental wild-type strain^a^ (μM)	QcrB_T313I_ (μM)
**13c**	1	Br	CH_3_	OCF_3_	3.4	4.0
**16c**	1	Br	CH_3_	Et	2.0	2.4
**17c**	1	Br	CH_3_	*m*,*p*-Ph ext.	2.5	2.2
**9d**	1	I	CH_3_	H	0.95	0.67
**10d**	1	I	CH_3_	OCH_3_	0.92	1.2
**11d**	1	I	CH_3_	Cl	0.84	0.80
**13d**	1	I	CH_3_	OCF_3_	3.7	5.2
**15d**	1	I	CH_3_	Me	0.48	0.70
**16d**	1	I	CH_3_	Et	1.9	1.8
**17d**	1	I	CH_3_	*m*,*p*-Ph ext.	1.4	1.2
**18a**	2	H	CH_3_	H	1.6	1.0
**18b**	2	OCH_3_	CH_3_	H	1.5	1.3
**18c**	2	Br	CH_3_	H	0.28	<0.19
**18d**	2	I	CH_3_	H	0.58	0.48
**18e**	2	H	H	H	24.0	29.0
**20 (RIF)**	–	–	–	–	0.030	0.010

a H37Rv-LP (ATCC 25618). *m*,*p*-Ph ext., 2-naphthyl group. RIF, rifampicin.

Further mechanistic studies were conducted on the 15 selected 4-aminoquinazolines. None of the compounds disrupted the bacillary membrane potential (Δψ) or induced reactive oxygen species (ROS) production within their MIC range (see the Supporting Information). Additionally, the molecules were tested against an *M. tuberculosis* H37Rv-LP mutant strain harboring three mutations in the MmpL3 transporter (F255L, V646M, and F644I), which confer resistance to multiple structurally diverse inhibitors, that target MmpL3 including AU1235.^35^ No significant differences in MIC values were observed compared to the parental strain (H37Rv-LP), indicating that MmpL3 is unlikely to be the molecular target of this compound series (see the Supporting Information).

The cytotoxicity and selectivity profiles of the selected 4-aminoquinazolines were evaluated in HepG2 and Vero cell lines using two complementary assays: MTT, which assesses mitochondrial metabolic activity, and neutral red (NR) uptake, which reflects lysosomal integrity. From these assays, CC_50_ values and corresponding selectivity indices (SI = CC_50_/MIC_H37Rv-LP) were determined (Table 3). The MTT assay revealed a clear trend among the halogenated derivatives at the 6-position. Brominated analogues, such as **13c** and **16c**–**17c**, exhibited marked cytotoxicity across both cell lines (CC_50_ = <1 μM to 5.1 μM), while iodine-substituted counterparts were notably less toxic. Compounds **10d** and **11d** demonstrated acceptable selectivity in HepG2 cells (SI = 12 for both), although **11d** significantly reduced viability of Vero cells (CC_50_ = 2.3 μM). The incorporation of a methyl group at R^4^, as in molecule **15d**, appeared beneficial, enhancing selectivity in HepG2 (SI = 21). By contrast, substituents such as OCF_3_, ethyl, or naphthyl at the same position were associated with reduced selectivity (SI = 1.2–6.9). Further insights were gained from the evaluation of the homologous series **18a**–**e** (*n* = 2), which revealed a broad range of cytotoxicity profiles (CC_50_ = <1 μM to >20 μM). Notably, analogues lacking substitution at 2- and 6-positions, such as **18a** and **18e**, particularly reduced the HepG2 viability (CC_50_ = 3.7 and <1 μM, respectively). Although Vero cells displayed slightly greater resistance, the corresponding SIs remained low. In contrast, 4-aminoquinazolines **18b**–**18d** exhibited improved safety profiles (CC_50_ = 11.6 to >20 μM). Among these, **18b**, while not reaching SI > 10 in Vero cells, showed attenuated cytotoxicity (CC_50_ = 15 μM). Importantly, halogenation at the 6-position, as seen in **18c** and **18d**, positively impacted both antimycobacterial activity and host cell selectivity. Compound **18c** emerged as a promising candidate, exhibiting a 21-fold higher SI in HepG2 and 8-fold in Vero, compared to its unsubstituted analogue **18a**. A comparison between structures **9d** (*n* = 1) and **18d** (*n* = 2) further suggested that linker elongation at the 4-position contributes to reduced cytotoxicity and improved selectivity. The NR uptake assay corroborated the MTT results, reinforcing the overall cytotoxicity trends. To explore whether mitochondrial dysfunction contributed to reduced viability, ATP quantification assays were performed in HepG2 cells cultured in glucose- and galactose-containing media (see the Supporting Information). The resulting CC_50_ values (1.5–20.3 μM) were consistent with those observed in MTT and NR assays. Importantly, no significant decrease in CC_50_ values was observed under galactose conditions, suggesting that mitochondrial dysfunction is not a major contributor to the observed reduction in cell viability induced by these compounds.

**Table 3:** Determination of Cell Viability of 4-Aminoquinazolines Using the MTT and NR Assays

entry	MIC^a^ (μM)	CC_50_ HepG2^b^ (μM)	CC_50_ Vero^b^ (μM)	SI^e^ HepG2	SI^e^ Vero
**13c**	3.4	<1;^c^ <1^d^	<1;^c^ 3.2^d^	<1;^c^ <1^d^	<1;^c^ <1^d^
**16c**	2.0	1.6;^c^ 1.8^d^	1.8;^c^ 5.6^d^	<1;^c^ < 1^d^	<1;^c^ 2.8^d^
**17c**	2.5	<1;^c^ <1^d^	5.1;^c^ 6.6^d^	<1;^c^ <1^d^	2.0;^c^ 2.6^d^
**9d**	0.95	8.5;^c^ 7.9^d^	11.7;^c^ 11.7^d^	9.0;^c^ 8.3^d^	12.3;^c^ 12.3^d^
**10d**	0.92	11.1;^c^ 8.5^d^	9.0;^c^ 15.9^d^	12.1;^c^ 9.2^d^	9.8;^c^ 17.3^d^
**11d**	0.84	10.5;^c^ 6.6^d^	2.3;^c^ 11.8^d^	13.0;^c^ 7.9^d^	2.70;^c^ 14.0^d^
**13d**	3.7	7.4;^c^ 7.3^d^	4.4;^c^ 2.3^d^	2.0;^c^ 2.0^d^	1.2;^c^ <1^d^
**15d**	0.48	10.2;^c^ 10.6^d^	10.7;^c^ 12.6^d^	21.3;^c^ 22.1^d^	22.3;^c^ 26.3^d^
**16d**	1.9	8.3;^c^ 8.3^d^	6.0;^c^ 8.9^d^	4.4;^c^ 4.4^d^	3.2;^c^ 4.7^d^
**17d**	1.4	6.9;^c^ 4.7^d^	9.7;^c^ 10.4^d^	4.9;^c^ 3.4^d^	6.9;^c^ 7.4^d^
**18a**	1.6	3.7;^c^ 1.0^d^	7.8;^c^ 9.6^d^	2.3;^c^ <1^d^	4.9;^c^ 6.0^d^
**18b**	1.5	>20;^c^ 17.0^d^	15.0;^c^ 17.4^d^	>13.0;^c^ 11.3^d^	9.7;^c^ 11.6^d^
**18c**	0.28	13.8;^c^ 13.2^d^	11.6;^c^ 14.0^d^	49.3;^c^ 47.1^d^	41.4;^c^ 50.0^d^
**18d**	0.58	>20;^c^ 18.3^d^	14.9;^c^ 16.1^d^	>34.5;^c^ 31.6^d^	25.7;^c^ 27.8^d^
**18e**	24.0	<1;^c^ <1^d^	3.6;^c^ 8.5^d^	<1;^c^ <1^d^	<1;^c^ <1^d^

a H37Rv-LP (ATCC 25618).

b The cell viability and selectivity index (SI) of the compounds were evaluated in HepG2 and Vero cell lines. Cell viability was measured using MTT and Neutral Red (NR) assays, with results expressed as the concentration required to reduce viability by 50% (CC_50_).

c Determined by the MTT method.

d Determined by the NR assay.

e Selectivity index (SI = CC_50_/MICH37Rv-LP).

Furthermore, based on their antimycobacterial activity and favorable selectivity profiles in mammalian cells, the 4-aminoquinazolines **18b**–**18d** were selected for additional screening against a broader panel of bacterial strains, including *Staphylococcus aureus* (ATCC 29213), *Escherichia coli* RFM 795, *Mycobacterium abscessus* 103R, *Mycobacterium avium* 2285S, *Mycobacterium avium* 2285R, and *Mycobacterium smegmatis* mc^2^155. Except for derivative **18b**, none of the tested compounds exhibited activity against the evaluated bacterial strains at the highest concentration tested (MIC = >12.4 μM to >100 μM), suggesting a high degree of selectivity toward *M. tuberculosis* (see the Supporting Information). Compound **18b** showed moderate activity against *E. coli* RFM 795, with an MIC of 54 μM. Notably, this strain carries a mutation in the *lptD* gene, which encodes a key protein involved in lipopolysaccharide assembly. Disruption or deletion of *lptD* impairs outer membrane integrity and permeability, underscoring its essential role in maintaining the bacterial envelope.^36^

Afterward, the microsomal stability of 4-aminoquinazolines **18b**–**18d** was evaluated in the presence of mice liver microsomes (MLM) and human liver microsomes (HLM) (see the Supporting Information). All evaluated compounds exhibited high intrinsic clearance in both systems. Correspondingly, their half-lives (*t*_1/2_) were short, ranging from 2.6 to 6.9 min in MLM and from 5.5 to 7.5 min in HLM. These findings suggest that limited metabolic stability may represent a liability for the in-vivo efficacy of this chemical series. Nonetheless, it is important to highlight that some drugs with low microsomal stability—such as verapamil—can still exhibit clinical utility, depending on other pharmacokinetic and pharmacodynamic properties.^37^

Finally, to assess the ability of 4-aminoquinazolines **18b**–**18d** to inhibit *M. tuberculosis* within host cells, intracellular activity was evaluated using infected THP-1 macrophage-like cells. THP-1 cells are widely employed in host–pathogen interaction studies due to their immunological and functional resemblance to human macrophages.^38^ Evaluated molecules demonstrated intracellular activity comparable to that observed under extracellular conditions (Table 4). Substitution at 2- and 6-positions of the quinazoline scaffold appeared to enhance intracellular efficacy. Compounds **18b** (R^1^ = OCH_3_; R^2^ = CH_3_) and **18d** (R^1^ = I; R^2^ = CH_3_) exhibited IC_90_ values ranging from 1.2 to 1.9 μM. Notably, structure **18c** (R^1^ = Br; R^2^ = CH_3_) was the most potent, with an IC_90_ of 0.45 μM. The ability of 4-aminoquinazolines to inhibit *M. tuberculosis* in both extracellular and intracellular environments support the potential of this chemical class in anti-TB drug discovery campaigns.

**Table 4:**
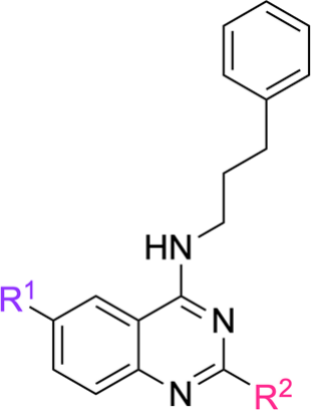
Intracellular Activity of 4-Aminoquinazolines in a Macrophage Model of *M. tuberculosis* Infection

entry	R^1^	R^2^	IC_50_ ^a^ (μM)	IC_90_ ^b^ (μM)
**18b**	OCH_3_	CH_3_	0.84	1.9
**18c**	Br	CH_3_	0.16	0.45
**18d**	I	CH_3_	0.47	1.2

a Intracellular IC_50_ is the concentration required to inhibit growth of H37Rv-LP (ATCC 25618) inside THP-1 cells by 50%.

b Intracellular IC_90_ is the concentration required to inhibit growth of H37Rv-LP (ATCC 25618) inside THP-1 cells by 90%.

Therefore, in this study, a series of 4-aminoquinazolines was designed through a scaffold hopping approach based on the pharmacophoric features of known antimycobacterial compounds. The target molecules were synthesized via a one-pot silylation–amination protocol under solvent-free conditions, affording the desired structures in 70%–99% yields. Furthermore, the antimycobacterial activity of the synthesized structures was thoroughly evaluated using different *M. tuberculosis* strains and complementary assay protocols, reinforcing the robustness of the findings.

The most active derivatives inhibited bacillary growth with MIC values as low as 0.45 μM (REMA) or 0.28 μM (fluorescence-based assay). Importantly, the study yielded clear and valuable SAR insights. In particular, the identification of the 2­(R^2^),6­(R^1^)-substituted *N*-(3-phenylpropyl)­quinazolin-4-amine scaffold—represented by compounds **18b**–**18d** (R^1^ = OCH_3_, Br, I; R^2^ = CH_3_)—was a key outcome. Mechanistic investigations revealed that 4-aminoquinazoline derivatives are unlikely to act through inhibition of the QcrB subunit of the cytochrome *bc*_1_ complex. The absence of MIC shifts in the QcrB_T313I_ mutant strain suggests that the quinazoline core lacks some essential molecular features for interaction with the Q_P_ site. In addition, the compounds did not disrupt membrane potential, induce reactive oxygen species (ROS), or appear to target the MmpL3 transporter, ruling out several common mechanisms of action. Additionally, the selectivity of the synthesized molecules was supported by cytotoxicity profiling using mammalian cell lines, where most derivatives showed favorable selectivity index. Moreover, no significant antibacterial activity was observed against a panel of other bacterial species, suggesting selective activity against *M. tuberculosis*. Crucially, representative structures from this series demonstrated potent intracellular activity in a macrophage infection model (THP-1 cells), highlighting their ability to reach and act on bacilli internalized within host cells. This is particularly relevant given the intracellular nature of *M. tuberculosis* during infection and supports the notion that these compounds possess suitable physicochemical properties for cellular permeability and retention.

Finally, taken together, the results demonstrate that 4-aminoquinazolines exhibit potent and selective antimycobacterial activity through a mechanism distinct from known and structurally related compounds—likely involving a novel, yet unidentified molecular target. These findings support the potential of this scaffold as a starting point for future anti-TB drug discovery efforts.

## Supplementary Material



## Notes

Materials and methods, synthetic procedures, analytical, spectroscopy and spectrometry data, biological assay protocols (MIC determination against wild-type and mutant *M. tuberculosis* strains, intracellular activity, ATP depletion, ROS generation, membrane potential, antimicrobial spectrum, and cell viability), in silico analyses, and microsomal stability studies. Additional experimental results and ^1^H and ^13^C NMR spectra for all synthesized compounds are also provided (PDF)

## ABBREVIATIONS

MIC: minimum inhibitory concentration
HMDS: hexamethyldisilazane
REMA: resazurin reduction microplate assay
ROS: reactive oxygen species
Δψ: membrane potential
MmpL3: Mycobacterial protein Large 3
NR: neutral red
MLM: mouse liver microsomes
HLM: human liver microsomes
*t*_1/2_: half-life
*m*: *p*-Ph ext., 2-naphthyl group
*c* log*P*: calculated logarithm of the octanol/water partition coefficient
LptD: lipopolysaccharide assembly protein
