# Supplementary material for: Scaffold Hopping Strategy toward New 4‑Aminoquinazolines Active Against Extracellular and Intracellular Mycobacterium tuberculosis
Source: ACS Med Chem Lett. 2025 Jun 30;16(7):1410–9. doi: 10.1021/acsmedchemlett.5c00276 (PMC12257397; doi:10.1021/acsmedchemlett.5c00276)
Supplement: Supplementary file 1 [file ml5c00276_si_001.pdf]

Scaffold Hopping Strategy Toward New 4-Aminoquinazolines Active Against Extracellular and Intracellular *Mycobacterium tuberculosis*

Guilherme Arraché Gonçalves[1,2], Alexia de Matos Czeczot[1], Marcia Alberton Perelló[1], Eric Greve[3], Renee Allen[3], Camili Zanella Zotti[1], Laura Calle González[1,4], Andresa Berger[1,4], Josiane Delgado Paz[1], Lídia Klatt Oliveira[1], Sidnei Moura e Silva[5], Cristiano Valim Bizarro[1,4], Luiz Augusto Basso[1,2,4], Tanya Parish*[3,6], Pablo Machado*[1,2,4]

[1] Instituto Nacional de Ciência e Tecnologia em Tuberculose, Centro de Pesquisas em Biologia Molecular e Funcional, Pontifícia Universidade Católica do Rio Grande do Sul, 90616-900 Porto Alegre, Rio Grande do Sul, Brazil

[2] Programa de Pós-Graduação em Medicina e Ciências da Saúde, Pontifícia Universidade Católica do Rio Grande do Sul, 90616-900 Porto Alegre, Rio Grande do Sul, Brazil

[3] Center for Global Infectious Disease Research, Seattle Children's Research Institute, Seattle, Washington, United States of America

[4] Programa de Pós-Graduação em Biologia Celular e Molecular, Pontifícia Universidade Católica do Rio Grande do Sul, 90616-900 Porto Alegre, Rio Grande do Sul, Brazil

[5] Laboratório de Biotecnologia de Produtos Naturais e Sintéticos, Universidade de Caxias do Sul, 95070-560 Caxias do Sul, Rio Grande do Sul, Brazil

[6] Department of Pediatrics, University of Washington School of Medicine, Seattle, Washington 98109, United States of America

*Corresponding author:

Tanya Parish (Tanya.Parish@seattlechildrens.org);

Pablo Machado (pablo.machado@pucrs.br).

Supporting Information

TABLE OF CONTENTS

1. Materials and methods 3

1.1. General methods 3

1.2. Chemistry 4

1.3. Biological assays 27

1.3.1. Determination of minimum inhibitory concentration (ATCC 27294) 27

1.3.2. Determination of minimum inhibitory concentration (ATCC 25618) 28

1.3.3. Determination of minimum inhibitory concentration in the mutant strains LP-0106898-RM1 (QcrB T313I) and LP-0497754-RM301 (MmpL3) of *M. tuberculosis* 28

1.3.4. Intracellular activity 28

1.3.5. ATP depletion assay 29

1.3.6. Reactive Oxygen Species (ROS) assay 29

1.3.7. Membrane potential (Δψ) assay 30

1.3.8. Antimicrobial spectrum assays 30

1.3.9. Cell viability evaluation 32

1.4. In silico methods 34

1.5. Microsomal stability 35

2. Results 36

2.1. Determination of minimum inhibitory concentration against H37Rv-LP (ATCC 25618) and its mutants 36

2.2. ATP depletion assay 37

2.3. Reactive Oxygen Species (ROS) assay 38

2.4. Membrane potential (Δψ) assay 39

2.5. Antimicrobial spectrum assays 40

2.6. Microsomal stability 41

3. ^{1}H and ^{13}C NMR spectra of the synthesized compounds 43

4. References 78

Supporting Information

1. Materials and methods

1.1. General methods

All solvents and reagents, including the quinazolin-4(3*H*)-one precursor **5e**[1], were obtained from commercial sources and used without further purification. When relevant, the relative purity of some reagents was determined by high-performance liquid chromatography (HPLC). The progress of the reaction was monitored using thin-layer chromatography (TLC) with Merck TLC Silica gel 60 F254. Melting points (m.p.) were determined using a Microquímica MQAPF-302 apparatus and were uncorrected. Infrared (IR) spectra were recorded on Perkin-Elmer Spectrum 100 FT-IR spectrometer with a Universal ATR sampling accessory. ^{1}H and ^{13}C nuclear magnetic resonance (NMR) spectra were acquired on an Avance III HD Bruker spectrometer (Bruker Corporation, Fällanden, Switzerland) with standard pulse sequences operating at 400 MHz for ^{1}H nuclei and 100 MHz for ^{13}C nuclei. Chemical shifts (δ) were expressed in parts per million (ppm) relative to $CDCl_3$ or DMSO-d_6, which were used as the solvent, and to TMS, as an internal standard. High-resolution mass spectra (HRMS) analyses were performed on Bruker MicroTOF-QII using electrospray ionization (ESI) (University of Caxias do Sul, Brazil). Compound purity was measured using a Dionex UltiMate 3000 HPLC system (Thermo Fisher Scientific Inc., Waltham, MA, USA) equipped with a dual pump, automatic injector, and UV detector. Stock solutions (1.0 mg/mL) of each product were prepared in acetonitrile/methanol (1:1, v/v) and diluted to 0.5 mg/mL for analysis. For data acquisition and processing, calculations were performed using the Chromeleon 6.80 SR11 software Build 3160 (183147). The HPLC conditions: reversed-phase (RP) column, 5 µm Nucleodur C-18 (250 × 4.6 mm); flow rate, 1.5 mL/min; UV detection at 254 nm; 100% water (0.1% acetic acid) was maintained from 0 to 7 min, followed by a linear gradient from 100% water (0.1% acetic acid) to 90% acetonitrile/methanol (1:1, v/v) from 7 to 15 min and subsequently returned to 100% water (0.1% acetic acid) in 5 min and maintained for more 10 min. All the synthesized compounds were ≥ 98% pure. Importantly, no unexpected or unusually high safety hazards were encountered.

---

[1]Purity previously determined by HPLC (99.92% at 254 nm; t_R = 13.31). The HPLC conditions are the same as those used for the synthesized compounds.

1.2. Chemistry

The synthetic route consisted of two steps (**Scheme 1**). The Niementowski reaction provided the precursors **7a-7d**.[1] Subsequently, the silylation-amination reaction was carried out to obtain the products **9a-18e**.[2] The compounds **7a-7c, 9a-12a**, **9b-12b**, **9c-12c**, and **9e-12e** were synthesized following the method previously reported by our group.[2]

Scheme S1. Synthesis of 4-aminoquinazolines.[a]

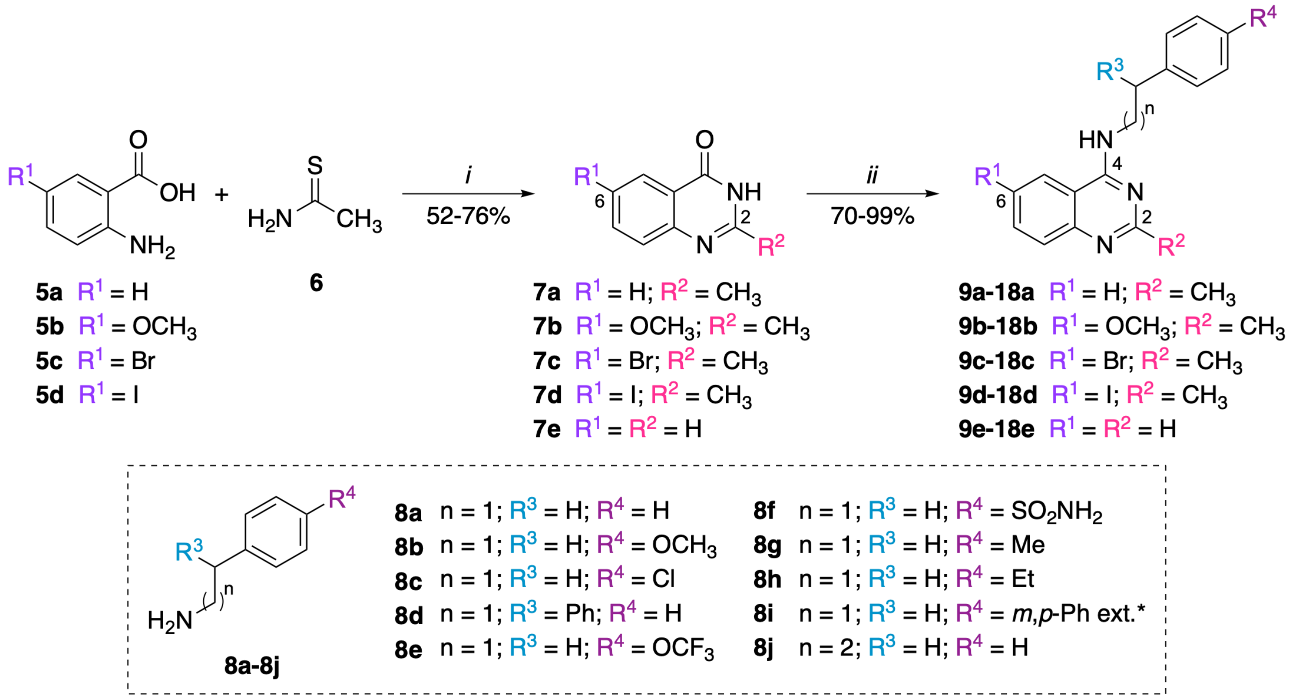

[a]Conditions and reagents are as follows: *i*) T = 150 °C, 2 h, 52-76%. *ii*): **8a-8j**, HMDS, $(NH_4)_2SO_4$, T = 125 °C, 24-48 h, 70-99%. **m*,*p*-Ph ext. (**8i**: 2-(naphthalen-2-yl)ethan-1-amine).

1.2.1. General procedure for the synthesis of 2-methylquinazolin-4(3*H*)-ones (7a-7d)

In a round-bottom flask, the appropriate substituted anthranilic acid **5a-5d** (14.58 mmol, 1.0 equiv.) and thioacetamide **6** (21.87 mmol, 1.5 equiv.) were refluxed at 150 °C for 2 h. After reaction completion, the crude solid was crystallized from ethanol or 2-propanol.[1]

Scheme S2. Synthesis of 2-methylquinazolin-4(3*H*)-ones (7a-7d).[a]

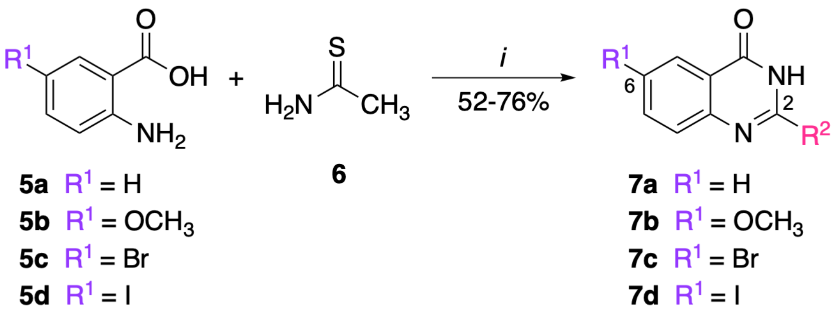

[a]Conditions and reagents are as follows: *i*) T = 150 °C, 2 h, 52-76%.

6-iodo-2-methylquinazolin-4(3*H*)-one (7d): crystallized from 2-propanol, yellowish white solid, yield: 56%, m.p. = 310-312 °C. HPLC: 99.61% at 254 nm (t_R = 16.36 min). FT-IR (ATR cm^{-1}): 1613-1674 (-C(=O)-NH-), 2877-3013 ($C\text{-}H_{arom}$), 3154 (N-H). ^1H NMR (400 MHz, DMSO-d_6) δ ppm: 2.33 (s, 3H, CH_3), 7.35 (d, *J* = 8.5 Hz, 1H, CH_{arom}), 8.02 (dd, *J* = 2.1, 8.6 Hz, 1H, CH_{arom}), 8.32 (d, *J* = 2.1 Hz, 1H, CH_{arom}), 12.28 (s, 1H, NH). ^{13}C (100 MHz, DMSO-d_6) δ ppm: 21.51, 90.08, 122.13, 128.38, 133.49, 141.99, 147.71, 154.51, 159.73.

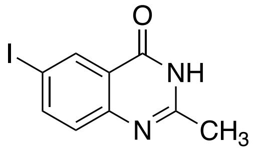

7d

1.2.2. General procedure for the synthesis of 4-aminoquinazolines (9a-18e)

In a round-bottom flask, the appropriate 2-methylquinazolin-4(3*H*)-one **7a-7e** (0.5 mmol, 1.0 equiv.), amine **8a-8j** (1.5 mmol, 3.0 equiv.), HMDS (1.5 mmol, 3.0 equiv.), and ammonium sulfate ($(NH_4)_2SO_4$) (0.05 mmol, 0.1 equiv.) were refluxed at 125 °C for 24–48 h. If the amine has a boiling point (b.p.) ≤ 125 °C (**8c**) or requires longer reaction times (**8c, 8d:** 48 h), the reaction was conducted in a Schlenk flask. Additionally, the reactions with the amine **8i** required 4.0 equiv. of HMDS. Upon completion, HMDS was removed under reduced pressure. The crude solid was purified by column chromatography using an appropriate solvent mixture (isocratic). The mobile phases used are specified in the description of each product.[2]

Scheme S3. Synthesis of 4-aminoquinazolines.[a]

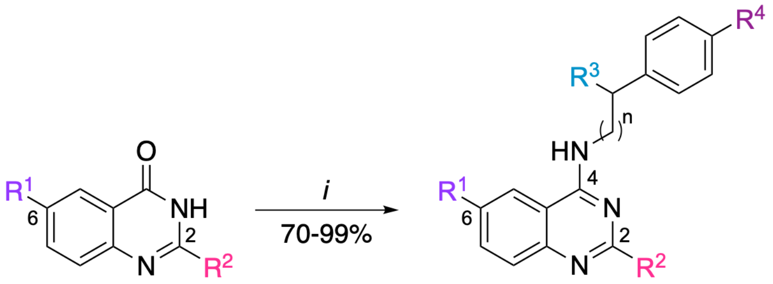

7a R^1 = H; R^2 = CH_3
7b R^1 = OCH_3; R^2 = CH_3
7c R^1 = Br; R^2 = CH_3
7d R^1 = I; R^2 = CH_3
7e R^1 = R^2 = H

9a-18a R^1 = H; R^2 = CH_3
9b-18b R^1 = OCH_3; R^2 = CH_3
9c-18c R^1 = Br; R^2 = CH_3
9d-18d R^1 = I; R^2 = CH_3
9e-18e R^1 = R^2 = H

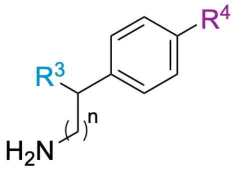

8a-8j

8a n = 1; R^3 = H; R^4 = H
8b n = 1; R^3 = H; R^4 = OCH_3
8c n = 1; R^3 = H; R^4 = Cl
8d n = 1; R^3 = Ph; R^4 = H
8e n = 1; R^3 = H; R^4 = OCF_3
8f n = 1; R^3 = H; R^4 = SO_2NH_2
8g n = 1; R^3 = H; R^4 = Me
8h n = 1; R^3 = H; R^4 = Et
8i n = 1; R^3 = H; R^4 = *m,p*-Ph ext.*
8j n = 2; R^3 = H; R^4 = H

**Entry**	n	R^1	R^2	R^3	R^4	t (h)	Yield (%)
**9a**	1	H	CH_3	H	H	24	85
**10a**	1	H	CH_3	H	OCH_3	24	84
**11a**	1	H	CH_3	H	Cl	48	97
**12a**	1	H	CH_3	Ph	H	48	89
**13a**	1	H	CH_3	H	OCF_3	24	92
**14a**	1	H	CH_3	H	SO_2NH_2	24	98

**15a**	1	H	CH_3	H	Me	24	90
**16a**	1	H	CH_3	H	Et	24	90
**17a**	1	H	CH_3	H	*m*,*p*-Ph ext.*	24	70
**18a**	2	H	CH_3	H	H	24	93
**9b**	1	OCH_3	CH_3	H	H	24	99
**10b**	1	OCH_3	CH_3	H	OCH_3	24	98
**11b**	1	OCH_3	CH_3	H	Cl	48	96
**12b**	1	OCH_3	CH_3	Ph	H	48	92
**13b**	1	OCH_3	CH_3	H	OCF_3	24	90
**14b**	1	OCH_3	CH_3	H	SO_2NH_2	24	95
**15b**	1	OCH_3	CH_3	H	Me	24	95
**16b**	1	OCH_3	CH_3	H	Et	24	96
**17b**	1	OCH_3	CH_3	H	*m*,*p*-Ph ext.*	24	77
**18b**	2	OCH_3	CH_3	H	H	24	98
**9c**	1	Br	CH_3	H	H	24	91
**10c**	1	Br	CH_3	H	OCH_3	24	96
**11c**	1	Br	CH_3	H	Cl	48	96
**12c**	1	Br	CH_3	Ph	H	48	92
**13c**	1	Br	CH_3	H	OCF_3	24	96
**14c**	1	Br	CH_3	H	SO_2NH_2	24	95
**15c**	1	Br	CH_3	H	Me	24	95
**16c**	1	Br	CH_3	H	Et	24	93
**17c**	1	Br	CH_3	H	*m*,*p*-Ph ext.*	24	77
**18c**	2	Br	CH_3	H	H	24	96
**9d**	1	I	CH_3	H	H	24	97
**10d**	1	I	CH_3	H	OCH_3	24	95
**11d**	1	I	CH_3	H	Cl	48	92
**12d**	1	I	CH_3	Ph	H	48	76
**13d**	1	I	CH_3	H	OCF_3	24	94
**14d**	1	I	CH_3	H	SO_2NH_2	24	98
**15d**	1	I	CH_3	H	Me	24	97

**16d**	1	I	CH_3	H	Et	24	91
**17d**	1	I	CH_3	H	*m*,*p*-Ph ext.*	24	81
**18d**	2	I	CH_3	H	H	24	94
**9e**	1	H	H	H	H	24	99
**10e**	1	H	H	H	OCH_3	24	96
**11e**	1	H	H	H	Cl	48	98
**12e**	1	H	H	Ph	H	48	95
**13e**	1	H	H	H	OCF_3	24	90
**14e**	1	H	H	H	SO_2NH_2	24	98
**15e**	1	H	H	H	Me	24	96
**16e**	1	H	H	H	Et	24	98
**17e**	1	H	H	H	*m*,*p*-Ph ext.*	24	86
**18e**	2	H	H	H	H	24	96

[a]Conditions and reagents are as follows: *i*) **8a-8j**, HMDS, $(NH_4)_2SO_4$, T = 125 °C, 24-48 h, 70-99%. **m*,*p*-Ph ext. (naphtyl group).

2-methyl-*N*-(4-(trifluoromethoxy)phenethyl)quinazolin-4-amine (13a): column chromatography on silica gel (ethyl acetate, 100%), white solid, yield: 92%, m.p. = 123-125 °C. HPLC: 99.21% at 254 nm (t_R = 14.96 min). FT-IR (ATR cm^{-1}): 1152-1250 (C-F), 1353 (C-N), 1572-1618 ($C{=}N_{quinazoline}$), 2932 ($-CH_2-$), 3058 ($C-H_{arom}$), 3204 (N-H). ^1H NMR (400 MHz, DMSO-d_6) δ ppm: 2.49 (s, 3H, CH_3), 3.02 (t, *J* = 7.3 Hz, 2H, CH_2), 3.79 (q, *J* = 6.8 Hz, 2H, CH_2); 7.28 (d, *J* = 8.2 Hz, 2H, CH_{arom}); 7.41 (d, *J* = 8.5 Hz, 2H, CH_{arom}); 7.47 (t, *J* = 7.7 Hz, 1H, CH_{arom}); 7.63 (d, *J* = 8.4 Hz, 1H, CH_{arom}); 7.75 (t, *J* = 7.5 Hz, 1H, CH_{arom}); 8.23 (d, *J* = 8.3 Hz, 1H, CH_{arom}); 8.65 (s, 1H, NH). ^{13}C (100 MHz, DMSO-d_6) δ ppm: 25.47, 33.57, 41.91, 112.68, 120.01 (q, *J* = 255.8 Hz, OCF_3), 120.74, 122.65, 125.02, 125.26, 130.41, 132.77, 139.01, 146.73, 147.50, 159.38, 160.92, 162.82. HRMS (ESI): *m/z* calc. for $C_{18}H_{17}F_3N_3O$ [M + H]$^+$: 348.1318; found, 348.1331.

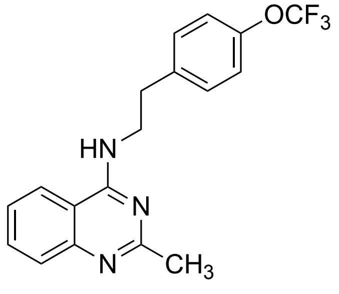

13a

4-(2-((2-methylquinazolin-4-yl)amino)ethyl)benzenesulfonamide (14a): column chromatography on silica gel (ethyl acetate/methanol, 95/5) followed by crystallization in ethanol*, yellow solid, yield: 98% (*89%, 2 crops), m.p. = 245-246 °C. HPLC: 99.68% at 254 nm (t_R = 13.06 min). FT-IR (ATR cm^{-1}): 1152 (S=O), 1312 (S=O), 1362 (C-N), 1579-1616 ($C=N_{quinazoline}$), 2957 ($-CH_2-$), 3281-3388 (N-H). ^1H NMR (400 MHz, DMSO-d_6) δ ppm: 2.47 (s, 3H, CH_3), 3.06 (t, *J* = 7.3 Hz, 2H, CH_2), 3.77 (q, *J* = 6.7 Hz, 2H, CH_2), 7.30 (s, 2H, NH_2), 7.41 (t, *J* = 7.6 Hz, 1H, CH_{arom}), 7.46 (d, *J* = 7.9 Hz, 2H, CH_{arom}), 7.59 (d, *J* = 8.3 Hz, 1H, CH_{arom}), 7.69 (t, *J* = 7.7 Hz, 1H, CH_{arom}), 7.75 (d, *J* = 8.0 Hz, 2H, CH_{arom}), 8.14 (d, *J* = 8.3 Hz, 1H, CH_{arom}), 8.25 (t, *J* = 5.6 Hz, 1H, CH_{arom}). ^{13}C (100 MHz, DMSO-d_6) δ ppm: 26.30, 34.12, 41.60, 112.99, 122.32, 124.50, 125.63, 126.83, 129.06, 132.20, 141.99, 143.88, 149.64, 159.24, 163.25. HRMS (ESI): *m/z* calc. for $C_{17}H_{19}N_4O_2S$ $[M + H]^+$: 343.1223; found, 343.1238.

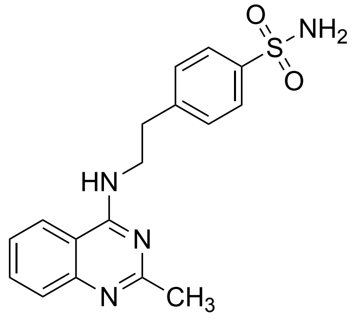

14a

2-metil-*N*-(4-metilfenetil)quinazolin-4-amina (15a): column chromatography on silica gel (ethyl acetate, 100%) followed by crystallization in hexane*, yellow solid, yield: 90% (*85%), m.p. = 139-140 °C. HPLC: 99.33% at 254 nm (t_R = 15.06 min). FT-IR (ATR cm^{-1}): 1350 (C-N), 1567-1615 ($C=N_{quinazoline}$), 2922 ($-CH_2-$), 3120 ($C-H_{arom}$), 3221 (N-H). ^1H NMR (400 MHz, $CDCl_3$) δ ppm: 2.34 (s, 3H, CH_3), 2.65 (s, 3H, CH_3), 2.97 (t, *J* = 6.9 Hz, 2H, CH_2), 3.90 (q, *J* = 6.6 Hz, 2H, CH_2), 5.79 (s, 1H, NH), 7.14 (s, 4H, CH_{arom}), 7.33 (t, *J* = 7.6 Hz, 1H, CH_{arom}), 7.51 (d, *J* = 8.3 Hz, 1H), 7.65 (t, *J* = 7.8 Hz, 1H, CH_{arom}), 7.74 (d, *J* = 8.4 Hz, 1H, CH_{arom}). ^{13}C (100 MHz, $CDCl_3$) δ ppm: 21.03, 26.69, 34.93, 42.25, 113.05, 120.28, 124.97, 127.83, 128.75, 129.45, 132.38, 135.93, 136.18, 150.08, 159.35, 164.50. HRMS (ESI): *m/z* calc. for $C_{18}H_{20}N_3$ $[M + H]^+$: 278.1652; found, 278.1663.

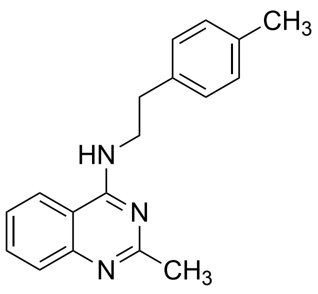

15a

***N*-(4-ethylphenethyl)-2-methylquinazolin-4-amine (16a):** column chromatography on silica gel (ethyl acetate, 100%) followed by crystallization in hexane*, white solid, yield: 90% (*80%), m.p. = 105-106 °C. HPLC: 99.86% at 254 nm (t_R = 15.47 min). FT-IR (ATR cm^{-1}): 1351 (C-N), 1571-1618 ($C=N_{quinazoline}$), 2930 ($-CH_2-$), 3052 ($C-H_{arom}$), 3223 (N-H). ^1H NMR (400 MHz, $CDCl_3$) δ ppm: 1.24 (t, *J* = 7.6 Hz, 3H, CH_3), 2.64 (m, 5H), 2.98 (t, *J* = 6.9 Hz, 2H, CH_2), 3.91 (q, *J* = 6.6 Hz, 2H, CH_2), 5.77 (s, 1H, NH), 7.17 (s, 4H, CH_{arom}), 7.33 (t, *J* = 7.6 Hz, 1H, CH_{arom}), 7.51 (d, *J* = 8.3 Hz, 1H, CH_{arom}), 7.65 (t, *J* = 7.4 Hz, 1H, CH_{arom}), 7.75 (d, *J* = 8.4 Hz, 1H, CH_{arom}). ^{13}C (100 MHz, $CDCl_3$) δ ppm: 15.57, 26.71, 28.49, 34.96, 42.24, 113.05, 120.26, 124.96, 127.87, 128.25, 128.81, 132.37, 136.19, 142.61, 150.13, 159.35, 164.52. HRMS (ESI): *m/z* calc. for $C_{19}H_{22}N_3$ $[M + H]^+$: 292.1808; found, 292.1820.

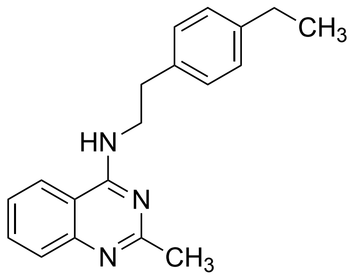

16a

2-methyl-*N*-(2-(naphthalen-2-yl)ethyl)quinazolin-4-amine (17a): column chromatography on silica gel (ethyl acetate, 100%) followed by crystallization in hexane:ethyl acetate (1:1)*, yellowish white solid, yield: 70% (*60%), m.p. = 142-143 °C. HPLC: 99.67% at 254 nm (t_R = 14.68 min). FT-IR (ATR cm^{-1}): 1350 (C-N), 1572-1615 ($C=N_{quinazoline}$), 2957 ($-CH_2-$), 3047 ($C-H_{arom}$), 3210 (N-H). ^1H NMR (400 MHz, $CDCl_3$) δ ppm: 2.67 (s, 3H, CH_3), 3.18 (t, *J* = 6.9 Hz, 2H, CH_2), 4.02 (q, *J* = 6.6 Hz, 2H, CH_2), 5.78 (s, 1H, NH), 7.23 – 7.33 (m, 1H), 7.38 – 7.41 (m, 1H), 7.45 – 7.47 (m, 2H), 7.62 – 7.69 (m, 2H), 7.73 – 7.84 (m, 5H). ^{13}C (100 MHz, $CDCl_3$) δ ppm: 26.74, 35.54, 42.06, 113.03, 120.24, 125.00, 125.61, 126.23, 127.24, 127.27, 127.50, 127.72, 127.89, 128.46, 132.38, 132.41, 133.69, 136.59, 150.14, 159.36, 164.50. HRMS (ESI): *m/z* calc. for $C_{21}H_{20}N_3$ $[M + H]^+$: 314.1652; found, 314.1658.

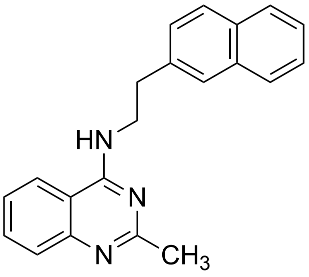

17a

2-metil-*N*-(3-fenilpropil)quinazolin-4-amina (18a): column chromatography on silica gel (ethyl acetate, 100%), white solid, yield: 93%, m.p. = 111 °C. HPLC: 99.85% at 254 nm (t_R = 14.03 min). FT-IR (ATR cm^{-1}): 1342 (C-N), 1571-1618 ($C=N_{quinazoline}$), 2924 ($-CH_2-$), 3025 ($C-H_{arom}$), 3224 (N-H). ^{1}H NMR (400 MHz, $CDCl_3$) δ ppm: 2.06 (p, *J* = 7.2 Hz, 2H, CH_2), 2.63 (s, 3H, CH_3), 2.77 (t, *J* = 7.4 Hz, 2H, CH_2), 3.72 (q, *J* = 6.8 Hz, 2H, CH_2), 5.75 (s, 1H, NH), 7.19 – 7.25 (m, 3H, CH_{arom}), 7.27 – 7.34 (m, 3H, CH_{arom}), 7.45 (d, *J* = 8.4 Hz, 1H, CH_{arom}), 7.64 (ddd, *J* = 1.4, 6.9, 8.4 Hz, 1H, CH_{arom}), 7.74 (d, *J* = 8.4 Hz, 1H, CH_{arom}). ^{13}C (100 MHz, $CDCl_3$) δ ppm: 26.69, 30.69, 33.63, 40.96, 112.97, 120.36, 124.85, 126.09, 127.77, 128.44, 128.60, 132.34, 141.62, 150.03, 159.41, 164.46. HRMS (ESI): *m/z* calc. for $C_{18}H_{20}N_3$ $[M + H]^+$: 278.1652; found, 278.1666.

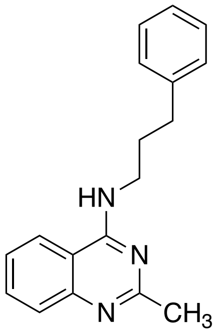

18a

6-methoxy-2-methyl-*N*-(4-(trifluoromethoxy)phenethyl)quinazolin-4-amine (13b): column chromatography on silica gel (ethyl acetate, 100%) followed by crystallization in hexane*, white solid, yield: 90% (*70%), m.p. = 182-183 °C. HPLC: 99.34% at 254 nm (t_R = 14.80 min). FT-IR (ATR cm^{-1}): 1160-1245 (C-F), 1353 (C-N), 1580-1629 ($C=N_{quinazoline}$), 2934 ($-CH_2-$), 3069 ($C-H_{arom}$), 3214 (N-H). ^{1}H NMR (400 MHz, DMSO-d_6) δ ppm: 2.50 (s, 3H, CH_3), 3.03 (t, *J* = 7.4 Hz, 2H, CH_2), 3.82 (q, *J* = 6.9 Hz, 2H, CH_2), 3.88 (s, 3H, OCH_3), 7.26 (d, *J* = 8.1 Hz, 2H, CH_{arom}), 7.40 (d, *J* = 8.4 Hz, 2H, CH_{arom}), 7.44 (dd, *J* = 2.6, 9.0 Hz, 1H, CH_{arom}), 7.64 (d, *J* = 9.1 Hz, 1H, CH_{arom}), 7.83 (d, *J* = 2.6 Hz, 1H, CH_{arom}), 9.16 (s, 1H, NH). ^{13}C (100 MHz, DMSO-d_6) δ ppm: 24.05, 33.57, 42.18, 56.01, 103.23, 112.92, 120.02 (q, *J*

= 255.5 Hz, OCF_3), 120.78, 124.30, 124.59, 130.46, 138.83, 146.78, 157.11, 159.04, 160.06. HRMS (ESI): *m/z* calc. for $C_{19}H_{19}F_3N_3O_2$ $[M + H]^+$: 378.1424; found, 378.1435.

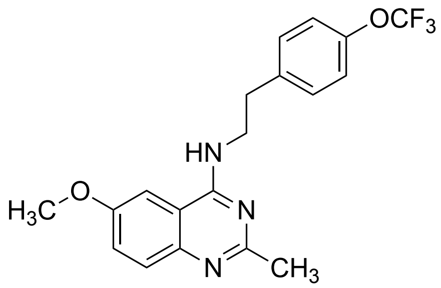

13b

4-(2-((6-methoxy-2-methylquinazolin-4-yl)amino)ethyl)benzenesulfonamide (14b): column chromatography on silica gel (ethyl acetate/methanol, 98/2) followed by crystallization in ethanol*, yellowish white solid, yield: 95% (*84%, 2 crops), m.p. = 232 °C. HPLC: 99.99% at 254 nm (t_R = 12.85 min). FT-IR (ATR cm^{-1}): 1146 (S=O), 1245 (C-O), 1309 (S=O), 1356 (C-N), 1583-1625 ($C{=}N_{quinazoline}$), 2966 ($-CH_2-$), 3182-3382 (N-H). ^1H NMR (400 MHz, DMSO-d_6) δ ppm: 2.45 (s, 3H, CH_3), 3.06 (t, *J* = 7.5 Hz, 2H, CH_2), 3.77 (q, *J* = 6.7 Hz, 2H, CH_2), 3.85 (s, 3H, OCH_3), 7.31 (s, 2H, NH_2), 7.34 (dd, *J* = 2.6, 8.9 Hz, 1H, CH_{arom}), 7.47 (d, *J* = 8.0 Hz, 2H, CH_{arom}), 7.54 (d, *J* = 9.1 Hz, 1H, CH_{arom}), 7.58 (d, *J* = 2.7 Hz, 1H, CH_{arom}), 7.77 (d, *J* = 8.0 Hz, 2H, CH_{arom}), 8.11 (t, *J* = 5.7 Hz, 1H, NH). ^{13}C (100 MHz, DMSO-d_6) δ ppm: 26.03, 34.28, 41.70, 55.67, 102.13, 113.32, 123.02, 125.66, 128.38, 129.07, 142.01, 143.94, 144.87, 156.19, 158.69, 160.99. HRMS (ESI): *m/z* calc. for $C_{18}H_{21}N_4O_3S$ $[M + H]^+$: 373.1329; found, 373.1348.

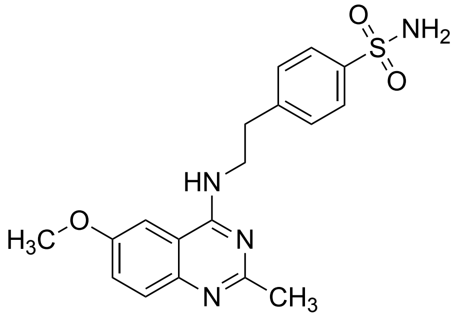

14b

6-methoxy-2-methyl-*N*-(4-methylphenethyl)quinazolin-4-amine (15b): column chromatography on silica gel (hexane/ethyl acetate, 25/75) followed by crystallization in hexane:ethyl acetate* (7:3) at 0 °C, yellow solid, yield: 96% (*85%), m.p. = 176-177 °C. HPLC: 99.43% at 254 nm (t_R = 15.73 min). FT-IR (ATR cm^{-1}): 1243 (C-O), 1351 (C-N), 1575-1626 ($C{=}N_{quinazoline}$), 2928 ($-CH_2-$), 3124 ($C{-}H_{arom}$), 3237 (N-H). ^1H NMR (400 MHz, $CDCl_3$) δ ppm: 2.34 (s, 3H, CH_3), 2.63 (s, 3H, CH_3), 2.98 (t, *J* = 7.0 Hz, 2H,

CH_2), 3.80 (s, 3H, OCH_3), 3.89 (q, *J* = 6.6 Hz, 2H, CH_2), 5.71 (s, 1H, NH), 6.80 (d, *J* = 2.6 Hz, 1H, CH_{arom}), 7.14 (s, 4H, CH_{arom}), 7.31 (dd, *J* = 2.5, 9.2 Hz, 1H, CH_{arom}), 7.68 (d, *J* = 9.0 Hz, 1H, CH_{arom}). ^{13}C (100 MHz, $CDCl_3$) δ ppm: 21.03, 26.41, 34.96, 42.35, 55.58, 100.23, 113.32, 123.20, 128.82, 129.37, 129.40, 136.09, 136.16, 145.46, 156.88, 158.76, 162.36. HRMS (ESI): *m/z* calc. for $C_{19}H_{22}N_3O$ [M + H]$^+$: 308.1757; found, 308.1765.

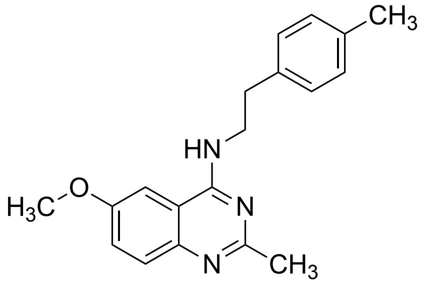

15b

***N*-(4-ethylphenethyl)-6-methoxy-2-methylquinazolin-4-amine (16b):** column chromatography on silica gel (ethyl acetate, 100%) followed by washing with hexane*, white solid, yield: 96% (*85%), m.p. = 185-186 °C. HPLC: 99.72% at 254 nm (t_R = 15.62 min). FT-IR (ATR cm^{-1}): 1246 (C-O), 1349 (C-N), 1578-1628 (C=$N_{quinazoline}$), 2935 (-CH_2-), 3070 (C-H_{arom}), 3213 (N-H). ^1H NMR (400 MHz, $CDCl_3$) δ ppm: 1.23 (t, *J* = 7.7 Hz, 3H, CH_3), 2.63 (s, 5H), 2.99 (t, *J* = 7.0 Hz, 2H, CH_2), 3.80 (s, 3H, OCH_3), 3.90 (q, *J* = 6.6 Hz, 2H, CH_2), 5.72 (s, 1H, NH), 6.80 (d, *J* = 2.7 Hz, 1H, CH_{arom}), 7.17 (s, 4H, CH_{arom}), 7.31 (dd, *J* = 2.6, 9.1 Hz, 1H, CH_{arom}), 7.68 (d, *J* = 9.1 Hz, 1H, CH_{arom}). ^{13}C (100 MHz, $CDCl_3$) δ ppm: 15.61, 26.40, 28.49, 34.99, 42.31, 55.57, 100.20, 113.32, 123.25, 128.20, 128.90, 129.36, 136.34, 142.61, 145.44, 156.89, 158.77, 162.36. HRMS (ESI): *m/z* calc. for $C_{20}H_{24}N_3O$ [M + H]$^+$: 322.1914; found, 322.1921.

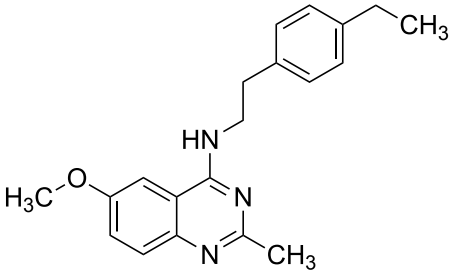

16b

6-methoxy-2-methyl-*N*-(2-(naphthalen-2-yl)ethyl)quinazolin-4-amine (17b): column chromatography on silica gel (hexane/ethyl acetate, 55/45) followed by crystallization in ethyl acetate*, white solid, yield: 77% (*64%, >2 crops), m.p. = 187-188 °C. HPLC: 99.68% at 254 nm (t_R = 15.85 min). FT-IR (ATR cm^{-1}): 1246 (C-O), 1349 (C-N), 1575-1628 (C=$N_{quinazoline}$), 2930 (-CH_2-), 3046

(C-H_{arom}), 3198 (N-H). ^1H NMR (400 MHz, DMSO-d_6) δ ppm: 2.47 (s, 3H, CH_3), 3.15 (t, *J* = 7.5 Hz, 2H, CH_2), 3.81 – 3.89 (m, 5H), 7.34 (dd, *J* = 2.7, 9.0 Hz, 1H, CH_{arom}), 7.42 – 7.49 (m, 3H, CH_{arom}), 7.54 (d, *J* = 9.0 Hz, 1H, CH_{arom}), 7.60 (d, *J* = 2.8 Hz, 1H, CH_{arom}), 7.77 – 7.88 (m, 4H, CH_{arom}), 8.15 (s, 1H, NH). ^{13}C (100 MHz, DMSO-d_6) δ ppm: 26.05, 34.75, 41.98, 55.64, 102.13, 113.35, 123.01, 125.20, 125.88, 126.58, 127.22, 127.37, 127.50, 127.67, 128.34, 131.66, 133.11, 137.37, 144.87, 156.17, 158.73, 161.01. HRMS (ESI): *m/z* calc. for $C_{22}H_{22}N_3O$ $[M + H]^+$: 344.1757; found, 344.1765.

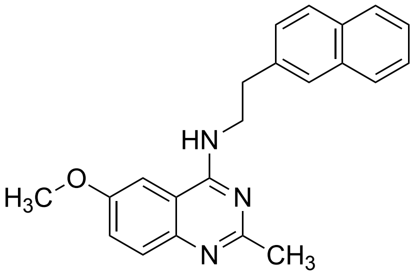

17b

6-methoxy-2-methyl-*N*-(3-phenylpropyl)quinazolin-4-amine (18b): column chromatography on silica gel (ethyl acetate, 100%), white solid, yield: 98%, m.p. = 185-186 °C. HPLC: 99.23% at 254 nm (t_R = 15.08 min). FT-IR (ATR cm^{-1}): 1249 (C-O), 1354 (C-N), 1578-1626 (C=$N_{quinazoline}$), 2925 (-CH_2-), 3047 (C-H_{arom}), 3259 (N-H). ^1H NMR (400 MHz, $CDCl_3$) δ ppm: 2.06 (p, *J* = 7.3 Hz, 2H, CH_2), 2.61 (s, 3H, CH_3), 2.76 (t, *J* = 7.5 Hz, 2H, CH_2), 3.72 (q, *J* = 6.7 Hz, 2H, CH_2), 3.80 (s, 3H, OCH_3), 5.89 (s, 1H, NH), 6.87 (d, *J* = 2.7 Hz, 1H, CH_{arom}), 7.17 – 7.22 (m, 3H, CH_{arom}), 7.26 – 7.32 (m, 3H, CH_{arom}), 7.68 (d, *J* = 9.1 Hz, 1H, CH_{arom}). ^{13}C (100 MHz, $CDCl_3$) δ ppm: 26.32, 30.73, 33.62, 41.05, 55.70, 100.50, 113.28, 123.23, 126.07, 128.43, 128.52, 129.12, 141.66, 145.22, 156.91, 158.91, 162.26. HRMS (ESI): *m/z* calc. for $C_{19}H_{22}N_3O$ $[M + H]^+$: 308.1757; found, 308.1767.

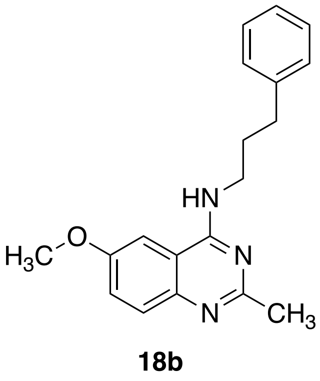

18b

6-bromo-2-methyl-*N*-(4-(trifluoromethoxy)phenethyl)quinazolin-4-amine (13c): column chromatography on silica gel (hexane/ethyl acetate, 65/35) followed by crystallization in hexane*,

white solid, yield: 96% (*80%), m.p. = 149-150 °C. HPLC: 99.99% at 254 nm (t_R = 15.29 min). FT-IR (ATR cm^{-1}): 1153-1245 (C-F), 1351 (C-N), 1566-1611 (C=N$_{quinazoline}$), 2969 (-CH$_2$-), 3100 (C-H$_{arom}$), 3237 (N-H). ^{1}H NMR (400 MHz, DMSO-d$_6$) δ ppm: 2.58 (s, 3H, CH$_3$), 3.04 (t, *J* = 7.3 Hz, 2H, CH$_2$), 3.87 (q, *J* = 6.8 Hz, 2H, CH$_2$), 7.27 (d, *J* = 8.1 Hz, 2H, CH$_{arom}$), 7.41 (d, *J* = 8.3 Hz, 2H, CH$_{arom}$), 7.78 (d, *J* = 8.9 Hz, 1H, CH$_{arom}$), 8.07 (d, *J* = 9.2 Hz, 1H, CH$_{arom}$), 8.82 (s, 1H, CH$_{arom}$), 10.12 (s, 1H, NH). ^{13}C (100 MHz, DMSO-d$_6$) δ ppm: 22.87, 33.14, 42.59, 113.26, 119.14, 119.99 (q, *J* = 256.0 Hz, OCF$_3$), 120.79, 122.40, 126.34, 130.52, 137.65, 138.35, 139.56, 146.84, 158.84, 161.87. HRMS (ESI): *m/z* calc. for $C_{18}H_{16}BrF_3N_3O$ [M + H]$^+$: 426.0423; found, 426.0437.

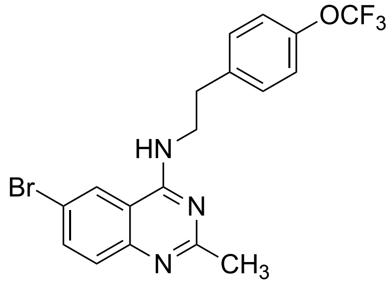

13c

4-(2-((6-bromo-2-methylquinazolin-4-yl)amino)ethyl)benzenesulfonamide (14c): column chromatography on silica gel (hexane/ethyl acetate, 2/8) followed by crystallization in ethanol*, white solid, yield: 95% (*87%, 2 crops), m.p. = 247 °C. HPLC: 98.99% at 254 nm (t_R = 13.04 min). FT-IR (ATR cm^{-1}): 1154 (S=O), 1336 (S=O), 1575-1609 (C=N$_{quinazoline}$), 2929 (-CH$_2$-), 3293 (N-H), 3419-3491(N-H). ^{1}H NMR (400 MHz, DMSO-d$_6$) δ ppm: 2.45 (s, 3H, CH$_3$), 3.04 (t, *J* = 7.3 Hz, 2H, CH$_2$), 3.75 (q, *J* = 6.8 Hz, 2H, CH$_2$), 7.29 (s, 2H, NH$_2$), 7.46 (d, *J* = 8.0 Hz, 2H, CH$_{arom}$), 7.53 (d, *J* = 8.8 Hz, 1H, CH$_{arom}$), 7.75 (d, *J* = 8.0 Hz, 2H, CH$_{arom}$), 7.82 (dd, *J* = 2.1, 8.9 Hz, 1H, CH$_{arom}$), 8.36 (t, *J* = 5.5 Hz, 1H, NH), 8.45 (d, *J* = 2.2 Hz, 1H, CH$_{arom}$). ^{13}C (100 MHz, DMSO-d$_6$) δ ppm: 26.27, 33.96, 41.68, 114.37, 116.68, 124.88, 125.63, 129.05, 129.17, 135.19, 142.02, 143.75, 148.56, 158.36, 163.90. HRMS (ESI): *m/z* calc. for $C_{17}H_{18}BrN_4O_2S$ [M + H]$^+$: 421.0328; found, 421.0348.

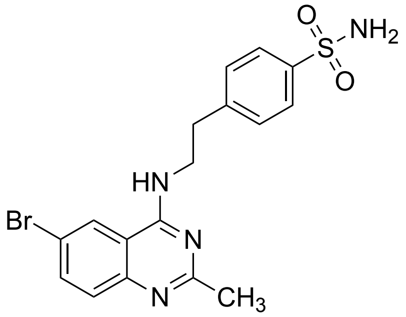

14c

6-bromo-2-methyl-*N*-(4-methylphenethyl)quinazolin-4-amine (15c): column chromatography on silica gel (hexane/ethyl acetate, 7/3) followed by crystallization in hexane*, white solid, yield: 95% (*80%), m.p. = 146-147 °C. HPLC: 99.93% at 254 nm (t_R = 15.82 min). FT-IR (ATR cm^{-1}): 1349 (C-N), 1565-1610 ($C{=}N_{quinazoline}$), 2924 ($-CH_2-$), 3098 ($C-H_{arom}$), 3239 (N-H). ^1H NMR (400 MHz, $CDCl_3$) δ ppm: 2.35 (s, 3H, CH_3), 2.63 (s, 3H, CH_3), 2.97 (t, J = 7.0 Hz, 2H, CH_2), 3.89 (q, J = 6.7 Hz, 2H, CH_2), 5.73 (s, 1H, NH), 7.14 (s, 4H, CH_{arom}), 7.60 (d, J = 8.8 Hz, 1H, CH_{arom}), 7.67 (d, J = 2.1 Hz, 1H, CH_{arom}), 7.71 (dd, J = 2.0, 8.9 Hz, 1H, CH_{arom}). ^{13}C (100 MHz, $CDCl_3$) δ ppm: 21.04, 26.66, 34.85, 42.44, 114.32, 117.92, 123.15, 128.72, 129.49, 129.69, 135.69, 135.75, 136.29, 148.93, 158.39, 164.96. HRMS (ESI): *m/z* calc. for $C_{18}H_{19}BrN_3$ $[M + H]^+$: 356.0757; found, 356.0767.

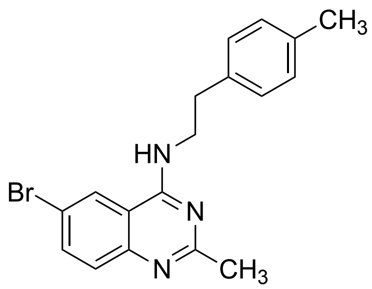

15c

6-bromo-*N*-(4-ethylphenethyl)-2-methylquinazolin-4-amine (16c): column chromatography on silica gel (hexane/ethyl acetate, 65/35) followed by washing with hexane*, white solid, yield: 93% (*61%), m.p. = 132-133 °C. HPLC: 99.20% at 254 nm (t_R = 15.52 min). FT-IR (ATR cm^{-1}): 1348 (C-N), 1567-1610 ($C{=}N_{quinazoline}$), 2930 ($-CH_2-$), 3104 ($C-H_{arom}$), 3230 (N-H). ^1H NMR (400 MHz, $CDCl_3$) δ ppm: 1.25 (t, J = 7.6 Hz, 3H, CH_3), 2.64 (d, J = 13.0 Hz, 5H), 2.98 (t, J = 7.0 Hz, 2H, CH_2), 3.90 (q, J = 6.7 Hz, 2H, CH_2), 5.73 (s, 1H. NH), 7.17 (s, 4H, CH_{arom}), 7.61 (d, J = 8.8 Hz, 1H, CH_{arom}), 7.67 (d, J = 2.1 Hz, 1H, CH_{arom}), 7.71 (dd, J = 2.0, 8.8 Hz, 1H, CH_{arom}). ^{13}C (100 MHz, $CDCl_3$) δ ppm: 15.58, 26.64, 28.50, 34.87, 42.42, 114.31, 117.94, 123.18, 128.29, 128.79, 129.62, 135.71, 135.99, 142.73, 148.83, 158.39, 164.94. HRMS (ESI): *m/z* calc. for $C_{19}H_{21}BrN_3$ $[M + H]^+$: 370.0913; found, 370.0925.

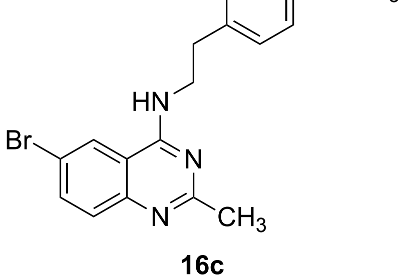

16c

6-bromo-2-methyl-*N*-(2-(naphthalen-2-yl)ethyl)quinazolin-4-amine (17c): column chromatography on silica gel (hexane/ethyl acetate, 4/6) followed by crystallization in ethyl acetate and washing with hexane*, white solid, yield: 77% (*55%, >2 crops), m.p. = 167-168 °C. HPLC: 99.86% at 254 nm (t_R = 15.70 min). FT-IR (ATR cm^{-1}): 1346 (C-N), 1566-1611 (C=$N_{quinazoline}$), 2963 (-CH_2-), 3036 (C-H_{arom}), 3236 (N-H). ^{1}H NMR (400 MHz, DMSO-d_6) δ ppm: 2.46 (s, 3H, CH_3), 3.13 (t, *J* = 7.5 Hz, 2H, CH_2), 3.82 (q, *J* = 6.8 Hz, 2H, CH_2), 7.42 – 7.49 (m, 3H, CH_{arom}), 7.53 (d, *J* = 8.8 Hz, 1H, CH_{arom}), 7.76 – 7.87 (m, 5H, CH_{arom}), 8.38 (s, 1H, NH), 8.47 (s, 1H, CH_{arom}). ^{13}C (100 MHz, DMSO-d_6) δ ppm: 26.29, 34.46, 41.97, 114.42, 116.65, 124.92, 125.23, 125.89, 126.59, 127.22, 127.37, 127.46, 127.67, 129.14, 131.67, 133.09, 135.16, 137.19, 148.58, 158.41, 163.94. HRMS (ESI): *m/z* calc. for $C_{21}H_{19}BrN_3$ $[M + H]^+$: 392.0757; found, 392.0760.

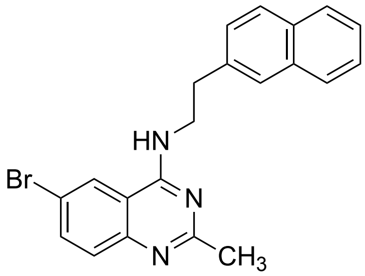

17c

6-bromo-2-methyl-*N*-(3-phenylpropyl)quinazolin-4-amine (18c): column chromatography on silica gel (hexane/ethyl acetate, 6/4), white solid, yield: 96%, m.p. = 143-144 °C. HPLC: 99.73% at 254 nm (t_R = 15.41 min). FT-IR (ATR cm^{-1}): 1344 (C-N), 1566-1610 (C=$N_{quinazoline}$), 2927 (-CH_2-), 3025 (C-H_{arom}), 3235 (N-H). ^{1}H NMR (400 MHz, $CDCl_3$) δ ppm: 2.08 (p, *J* = 7.1 Hz, 2H, CH_2), 2.60 (s, 3H, CH_3), 2.79 (t, *J* = 7.3 Hz, 2H, CH_2), 3.72 (q, *J* = 6.4 Hz, 2H, CH_2), 5.51 (s, 1H, NH), 7.23 – 7.34 (m, 5H, CH_{arom}), 7.44 (d, *J* = 2.1 Hz, 1H, CH_{arom}), 7.59 (d, *J* = 8.9 Hz, 1H, CH_{arom}), 7.70 (dd, *J* = 2.0, 8.9 Hz, 1H, CH_{arom}). ^{13}C (100 MHz, $CDCl_3$) δ ppm: 26.62, 30.44, 33.91, 41.30, 114.18, 117.83, 123.12, 126.49, 128.46, 128.75, 129.52, 135.64, 141.57, 148.73, 158.34, 164.87. HRMS (ESI): *m/z* calc. for $C_{18}H_{19}BrN_3$ $[M + H]^+$: 356.0757; found, 356.0764.

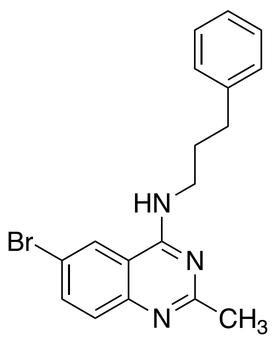

18c

6-iodo-2-methyl-*N*-phenethylquinazolin-4-amine (9d): column chromatography on silica gel (hexane/ethyl acetate, 7/3), white solid, yield: 97%, m.p. = 168-169 °C. HPLC: 99.51% at 254 nm (t_R = 14.97 min). FT-IR (ATR cm^{-1}): 1337 (C-N), 1586-1608 ($C{=}N_{quinazoline}$), 2952 ($-CH_2-$), 3036-3081 (C-H_{arom}), 3215 (N-H). ^1H NMR (400 MHz, $CDCl_3$) δ ppm: 2.63 (s, 3H, CH_3), 3.02 (t, *J* = 7.1 Hz, 2H, CH_2), 3.91 (q, *J* = 6.8 Hz, 2H, CH_2), 5.77 (s, 1H, NH), 7.22 – 7.31 (m, 3H), 7.35 (t, *J* = 7.4 Hz, 2H), 7.47 (d, *J* = 9.2 Hz, 1H, CH_{arom}), 7.86 – 7.91 (m, 2H). ^{13}C (100 MHz, $CDCl_3$) δ ppm: 26.71, 35.35, 42.38, 88.64, 114.99, 126.72, 128.79, 128.86, 129.58, 129.70, 138.91, 141.10, 149.30, 158.06, 165.07. HRMS (ESI): *m/z* calc. for $C_{17}H_{17}IN_3$ $[M + H]^+$: 390.0462; found, 390.0473.

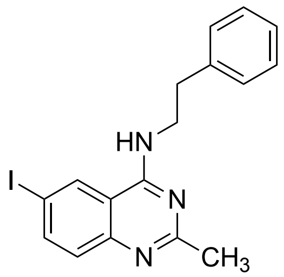

9d

6-iodo-*N*-(4-methoxyphenethyl)-2-methylquinazolin-4-amine (10d): column chromatography on silica gel (hexane/ethyl acetate, 45/55), yellowish white solid, yield: 95%, m.p. = 146-147 °C. HPLC: 99.38% at 254 nm (t_R = 14.88 min). FT-IR (ATR cm^{-1}): 1244 (C-O), 1346 (C-N), 1561-1606 ($C{=}N_{quinazoline}$), 2954 ($-CH_2-$), 3098 (C-H_{arom}), 3221 (N-H). ^1H NMR (400 MHz, $CDCl_3$) δ ppm: 2.62 (s, 3H, CH_3), 2.95 (t, *J* = 7.0 Hz, 2H, CH_2), 3.81 (s, 3H, OCH_3), 3.87 (q, *J* = 6.6 Hz, 2H, CH_2), 5.76 (s, 1H, NH), 6.88 (d, *J* = 8.2 Hz, 2H, CH_{arom}), 7.16 (d, *J* = 8.2 Hz, 2H, CH_{arom}), 7.47 (d, *J* = 9.2 Hz, 1H, CH_{arom}), 7.86 – 7.90 (m, 2H, CH_{arom}). ^{13}C (100 MHz, $CDCl_3$) δ ppm: 26.71, 34.42, 42.55, 55.35, 88.63, 114.28, 115.01, 129.60, 129.67, 129.80, 130.85, 141.08, 149.27, 158.07, 158.52, 165.07. HRMS (ESI): *m/z* calc. for $C_{18}H_{19}IN_3O$ $[M + H]^+$: 420.0567; found, 420.0586.

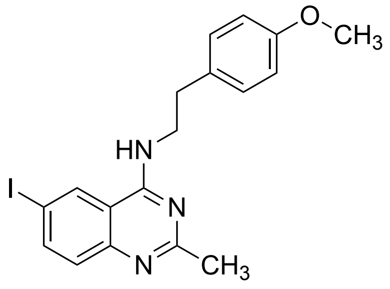

10d

***N*-(4-chlorophenethyl)-6-iodo-2-methylquinazolin-4-amine (11d):** column chromatography on silica gel (hexane/ethyl acetate, 65/35), yellowish white solid, yield: 92%, m.p. = 184-185 °C. HPLC: 99.70% at 254 nm (t_R = 15.50 min). FT-IR (ATR cm^{-1}): 1344 (C-N), 1561-1605 (C=N$_{quinazoline}$), 2924 (-CH$_2$-), 3092 (C-H$_{arom}$), 3224 (N-H). ^{1}H NMR (400 MHz, CDCl$_3$) δ ppm: 2.62 (s, 3H, CH$_3$), 2.99 (t, *J* = 7.2 Hz, 2H, CH$_2$), 3.89 (q, *J* = 6.7 Hz, 2H, CH$_2$), 5.85 (s, 1H, NH), 7.17 (d, *J* = 8.1 Hz, 2H, CH$_{arom}$), 7.29 (d, *J* = 8.0 Hz, 2H, CH$_{arom}$), 7.47 (d, *J* = 8.8 Hz, 1H, CH$_{arom}$), 7.89 (d, *J* = 8.8 Hz, 1H, CH$_{arom}$), 7.93 (s, 1H, CH$_{arom}$). ^{13}C (100 MHz, CDCl$_3$) δ ppm: 26.67, 34.69, 42.35, 88.82, 114.89, 128.84, 129.58, 130.17, 132.50, 137.39, 141.20, 149.15, 158.01, 164.96. HRMS (ESI): *m/z* calc. for $C_{17}H_{16}ClIN_3$ [M + H]$^+$: 424.0072; found, 424.0084.

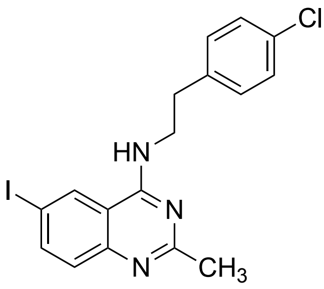

11d

***N*-(2,2-diphenylethyl)-6-iodo-2-methylquinazolin-4-amine (12d):** column chromatography on silica gel (hexane/ethyl acetate, 6/4), white solid, yield: 76%, m.p. = 206-207 °C. HPLC: 99.21% at 254 nm (t_R = 15.89 min). FT-IR (ATR cm^{-1}): 1349 (C-N), 1561-1607 (C=N$_{quinazoline}$), 2945 (-CH$_2$-), 3027-3059 (C-H$_{arom}$), 3249 (N-H). ^{1}H NMR (400 MHz, CDCl$_3$) δ ppm: 2.66 (s, 3H, CH$_3$), 4.32 (dd, *J* = 5.4, 7.8 Hz, 2H, CH$_2$), 4.48 (t, *J* = 7.7 Hz, 1H, CH), 5.75 (s, 1H, NH), 7.27 – 7.33 (m, 3H, CH$_{arom}$), 7.34 – 7.40 (m, 7H, CH$_{arom}$), 7.48 (d, *J* = 8.8 Hz, 1H, CH$_{arom}$), 7.75 (d, *J* = 1.9 Hz, 1H, CH$_{arom}$), 7.88 (dd, *J* = 1.8, 8.8 Hz, 1H, CH$_{arom}$). ^{13}C (100 MHz, CDCl$_3$) δ ppm: 26.64, 45.56, 50.09, 88.76, 114.88, 127.02, 128.20, 128.84, 129.46, 129.54, 141.18, 141.85, 149.05, 157.98, 164.96. HRMS (ESI): *m/z* calc. for $C_{23}H_{21}IN_3$ [M + H]$^+$: 466.0775; found, 466.0786.

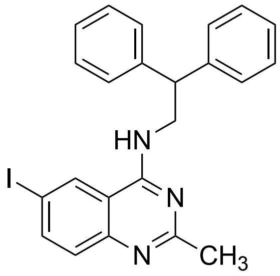

12d

6-iodo-2-methyl-*N*-(4-(trifluoromethoxy)phenethyl)quinazolin-4-amine (13d): column chromatography on silica gel (hexane/ethyl acetate, 6/4) followed by crystallization in hexane*, white solid, yield: 94% (*80%), m.p. = 150-151 °C. HPLC: 99.97% at 254 nm (t_R = 15.38 min). FT-IR (ATR cm^{-1}): 1153-1254 (C-F), 1349 (C-N), 1564-1607 ($C{=}N_{quinazoline}$), 2937 ($-CH_2-$), 3098 ($C{-}H_{arom}$), 3215 (N-H). ^1H NMR (400 MHz, DMSO-d_6) δ ppm: 2.46 (s, 3H, CH_3), 3.00 (t, *J* = 7.4 Hz, 2H, CH_2), 3.76 (q, *J* = 6.8 Hz, 2H, CH_2), 7.27 (d, *J* = 8.2 Hz, 2H, CH_{arom}), 7.38 – 7.43 (m, 3H, CH_{arom}), 8.00 (dd, *J* = 1.8, 8.7 Hz, 1H, CH_{arom}), 8.67 (s, 1H, CH_{arom}), 8.77 (s, 1H, NH). ^{13}C (100 MHz, DMSO-d_6) δ ppm: 25.40, 33.44, 42.04, 89.67, 114.54, 120.01 (q, *J* = 255.3 Hz, OCF_3), 120.77, 127.12, 130.41, 131.28, 138.90, 141.16, 146.75, 158.18, 163.27. HRMS (ESI): *m/z* calc. for $C_{18}H_{16}F_3IN_3O$ $[M + H]^+$: 474.0285; found, 474.0303.

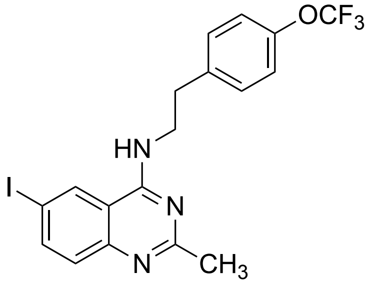

13d

4-(2-((6-iodo-2-methylquinazolin-4-yl)amino)ethyl)benzenesulfonamide (14d): column chromatography on silica gel (hexane/ethyl acetate, 2/8) followed by crystallization in ethanol*, white solid, yield: 98% (79%, 2 crops), m.p. = 250 °C. HPLC: 99.99% at 254 nm (t_R = 13.08 min). FT-IR (ATR cm^{-1}): 1155 (S=O), 1335 (S=O), 1357 (C-N), 1557-1602 ($C{=}N_{quinazoline}$), 2928 ($-CH_2-$), 3294-3398 (N-H). ^1H NMR (400 MHz, DMSO-d_6) δ ppm: 2.45 (s, 3H, CH_3), 3.04 (t, *J* = 7.3 Hz, 2H, CH_2), 3.75 (q, *J* = 6.7 Hz, 2H, CH_2), 7.29 (s, 2H, NH_2), 7.37 (d, *J* = 8.7 Hz, 1H, CH_{arom}), 7.46 (d, *J* = 8.0 Hz, 2H, CH_{arom}), 7.76 (d, *J* = 8.0 Hz, 2H, CH_{arom}), 7.94 (dd, *J* = 1.9, 8.7 Hz, 1H, CH_{arom}), 8.35 (t, *J* = 5.6 Hz, 1H, NH), 8.59 (s, 1H, CH_{arom}). ^{13}C (100 MHz, DMSO-d_6) δ ppm: 26.31, 33.99, 41.67, 88.92, 114.94, 125.63, 129.00,

129.04, 130.97, 140.53, 142.01, 143.78, 148.83, 158.04, 163.86. HRMS (ESI): *m/z* calc. for $C_{17}H_{18}IN_4O_2S$ $[M + H]^+$: 469.0190; found, 469.0198.

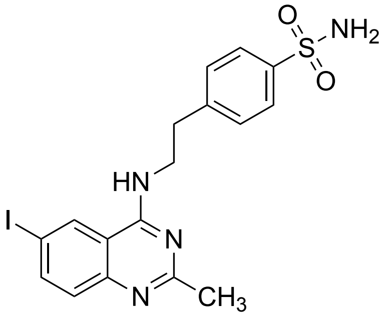

14d

6-iodo-2-methyl-*N*-(4-methylphenethyl)quinazolin-4-amine (15d): column chromatography on silica gel (hexane/ethyl acetate, 7/3) followed by crystallization in ethanol:water* (9:1) at 0 °C, yellow solid, yield: 97% (*80%), m.p. = 156-157 °C. HPLC: 99.99% at 254 nm (t_R = 16.00 min). FT-IR (ATR cm^{-1}): 1347 (C-N), 1560-1605 ($C{=}N_{quinazoline}$), 2917 ($-CH_2-$), 3098 ($C{-}H_{arom}$), 3223 (N-H). ^1H NMR (400 MHz, $CDCl_3$) δ ppm: 2.35 (s, 3H, CH_3), 2.62 (s, 3H, CH_3), 2.97 (t, J = 7.0 Hz, 2H, CH_2), 3.88 (q, J = 6.7 Hz, 2H, CH_2), 5.75 (s, 1H, NH), 7.15 (s, 4H, CH_{arom}), 7.47 (d, J = 9.1 Hz, 1H, CH_{arom}), 7.79 – 7.93 (m, 2H, CH_{arom}). ^{13}C (100 MHz, $CDCl_3$) δ ppm: 21.05, 26.70, 34.87, 42.45, 88.62, 115.00, 128.73, 129.48, 129.60, 129.67, 135.76, 136.29, 141.07, 149.28, 158.06, 165.08. HRMS (ESI): *m/z* calc. for $C_{18}H_{19}IN_3$ $[M + H]^+$: 404.0618; found, 404.0634.

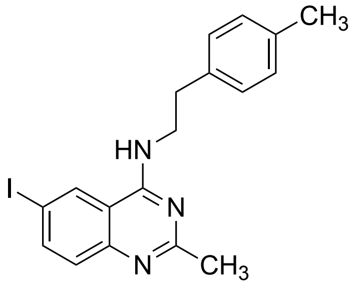

15d

***N*-(4-ethylphenethyl)-6-iodo-2-methylquinazolin-4-amine (16d):** column chromatography on silica gel (hexane/ethyl acetate, 6/4) followed by washing with hexane*, white solid, yield: 91% (*62%), m.p. = 143-144 °C. HPLC: 99.87% at 254 nm (t_R = 16.12 min). FT-IR (ATR cm^{-1}): 1347 (C-N), 1562-1605 ($C{=}N_{quinazoline}$), 2963 ($-CH_2-$), 3098 ($C{-}H_{arom}$), 3225 (N-H). ^1H NMR (400 MHz, $CDCl_3$) δ ppm: 1.25 (t, J = 7.6 Hz, 3H, CH_3), 2.61 – 2.68 (m, 5H), 2.98 (t, J = 7.0 Hz, 2H, CH_2), 3.89 (q, J = 6.7 Hz, 2H, CH_2), 5.73 (s, 1H, NH), 7.17 (s, 4H, CH_{arom}), 7.47 (d, J = 8.7 Hz, 1H, CH_{arom}), 7.84 – 7.92 (m, 2H, CH_{arom}). ^{13}C (100

MHz, $CDCl_3$) δ ppm: 15.60, 26.71, 28.50, 34.89, 42.40, 88.60, 115.01, 128.28, 128.81, 129.61, 129.68, 135.99, 141.06, 142.73, 149.29, 158.06, 165.08. HRMS (ESI): m/z calc. for $C_{19}H_{21}IN_3$ [M + H]+: 418.0775; found, 418.0791.

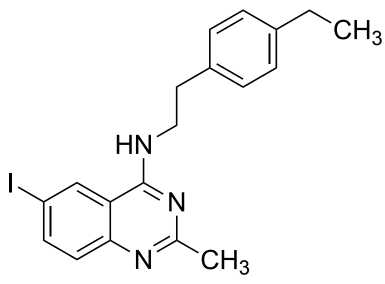

16d

6-iodo-2-methyl-*N*-(2-(naphthalen-2-yl)ethyl)quinazolin-4-amine (17d): column chromatography on silica gel (hexane/ethyl acetate, 4/6) followed by crystallization in acetone and washing in hexane*, white solid, yield: 81% (*60%), m.p. = 148-149 °C. HPLC: 99.72% at 254 nm (t_R = 15.62 min). FT-IR (ATR cm^{-1}): 1345 (C-N), 1562-1606 ($C{=}N_{quinazoline}$), 2961 ($-CH_2-$), 3042 ($C\text{-}H_{arom}$), 3234 (N-H). ^1H NMR (400 MHz, DMSO-d_6) δ ppm: 2.45 (s, 3H, CH_3), 3.13 (t, J = 7.5 Hz, 2H, CH_2), 3.83 (q, J = 6.8 Hz, 2H, CH_2), 7.37 (d, J = 8.7 Hz, 1H, CH_{arom}), 7.43 – 7.48 (m, 3H, CH_{arom}), 7.77 (s, 1H, CH_{arom}), 7.82 – 7.87 (m, 3H, CH_{arom}), 7.94 (dd, J = 1.9, 8.8 Hz, 1H, CH_{arom}), 8.38 (s, 1H, NH), 8.58 – 8.63 (m, 1H, CH_{arom}). ^{13}C (100 MHz, DMSO-d_6) δ ppm: 26.33, 34.47, 41.95, 88.85, 114.99, 125.21, 125.88, 126.57, 127.21, 127.35, 127.45, 127.66, 128.97, 131.00, 131.65, 133.08, 137.19, 140.48, 148.85, 158.07, 163.87. HRMS (ESI): *m/z* calc. for $C_{21}H_{19}IN_3$ $[M + H]^+$: 440.0618; found, 440.0628.

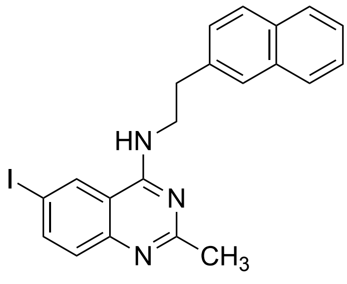

17d

6-iodo-2-methyl-*N*-(3-phenylpropyl)quinazolin-4-amine (18d): column chromatography on silica gel (hexane/ethyl acetate, 6/4), white solid, yield: 94%, m.p. = 153-154 °C. HPLC: 99.89% at 254 nm (t_R = 15.58 min). FT-IR (ATR cm^{-1}): 1341 (C-N), 1561-1605 ($C{=}N_{quinazoline}$), 2933 ($-CH_2-$), 3023 ($C\text{-}H_{arom}$), 3236 (N-H). ^1H NMR (400 MHz, $CDCl_3$) δ ppm: 2.08 (p, J = 7.1 Hz, 2H, CH_2), 2.59 (s, 3H, CH_3), 2.79 (t, J = 7.3 Hz, 2H, CH_2), 3.72 (q, J = 6.5 Hz, 2H, CH_2), 5.51 (s, 1H, NH), 7.25 – 7.38 (m, 5H, CH_{arom}), 7.45

(d, *J* = 8.8 Hz, 1H, CH_{arom}), 7.64 (d, *J* = 1.9 Hz, 1H, CH_{arom}), 7.87 (dd, *J* = 1.8, 8.8 Hz, 1H, CH_{arom}). ^{13}C (100 MHz, $CDCl_3$) δ ppm: 26.69, 30.45, 33.93, 41.29, 88.49, 114.88, 126.53, 128.45, 128.74, 129.44, 129.59, 141.02, 141.56, 149.20, 157.99, 165.00. HRMS (ESI): *m/z* calc. for $C_{18}H_{19}IN_3$ [M + H]$^+$: 404.0618; found, 404.0636.

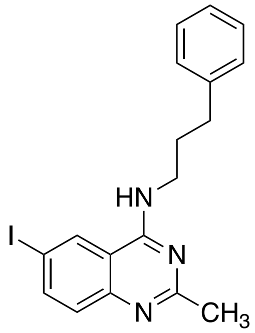

18d

***N*-(4-(trifluoromethoxy)phenethyl)quinazolin-4-amine (13e):** column chromatography on silica gel (hexane/ethyl acetate, 2/8), white solid, yield: 90%, m.p. = 132-133 °C. HPLC: 99.25% at 254 nm (t_R = 16.00 min). FT-IR (ATR cm^{-1}): 1124-1251 (C-F), 1354 (C-N), 1578-1621 (C=$N_{quinazoline}$), 2927-2966 (-CH_2-), 3072 (C-H_{arom}), 3045 (N-H). ^1H NMR (400 MHz, $CDCl_3$) δ ppm: 3.11 (t, *J* = 7.4 Hz, 2H, CH_2), 4.00 (s, 2H, CH_2), 4.57 (s, 1H, NH), 7.13 (d, *J* = 8.1 Hz, 2H, CH_{arom}), 7.29 (d, *J* = 8.6 Hz, 2H, CH_{arom}), 7.48 (t, *J* = 7.7 Hz, 1H, CH_{arom}), 7.74 (t, *J* = 7.8 Hz, 1H, CH_{arom}), 7.91 (d, *J* = 8.4 Hz, 1H, CH_{arom}), 8.16 (d, *J* = 8.3 Hz, 1H, CH_{arom}), 8.61 (s, 1H, $CH_{pyrimidine}$).. ^{13}C (100 MHz, $CDCl_3$) δ ppm: 34.47, 42.82, 114.41, 120.53 (q, *J* = 256.9 Hz, OCF_3), 121.18, 122.39, 125.11, 127.14, 130.20, 133.81, 137.51, 144.98, 148.06, 153.18, 160.09. HRMS (ESI): *m/z* calc. for $C_{17}H_{15}F_3N_3O$ [M + H]$^+$: 334.1162; found, 334.1174.

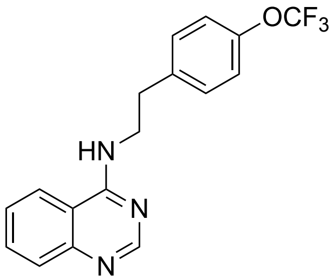

13e

4-(2-(quinazolin-4-ylamino)ethyl)benzenesulfonamide (14e): column chromatography on silica gel (ethyl acetate/methanol, 97/3), white solid, yield: 98%, m.p. = 225-226 °C. HPLC: 99.75% at 254 nm (t_R = 12.31 min). FT-IR (ATR cm^{-1}): 1157 (S=O), 1341 (S=O), 1357 (C-N), 1584-1615 (C=$N_{quinazoline}$), 2923 (-CH_2-), 3025 (C-H_{arom}), 3237-3366 (N-H). ^1H NMR (400 MHz, DMSO-d_6) δ ppm: 3.06 (t, *J* = 7.4

Hz, 2H, CH_2), 3.79 (q, *J* = 6.8 Hz, 2H, CH_2), 7.30 (s, 2H, NH_2), 7.44 – 7.52 (m, 3H, CH_{arom}), 7.68 (d, *J* = 8.2 Hz, 1H, CH_{arom}), 7.73 – 7.77 (m, 3H, CH_{arom}), 8.20 (d, *J* = 8.3 Hz, 1H, CH_{arom}), 8.39 (t, *J* = 5.7 Hz, 1H, NH), 8.50 (s, 1H, $CH_{pyrimidine}$). ^{13}C (100 MHz, DMSO-d_6) δ ppm: 34.09, 41.59, 114.90, 122.47, 125.49, 125.65, 127.44, 129.06, 132.38, 142.02, 143.73, 149.02, 155.02, 159.25. HRMS (ESI): *m/z* calc. for $C_{16}H_{17}N_4O_2S$ $[M + H]^+$: 329.1067; found, 329.1080.

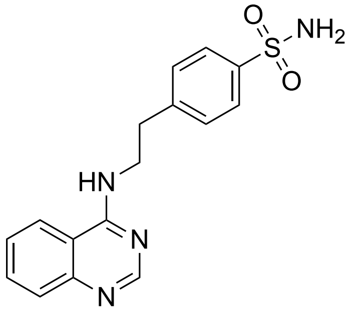

14e

***N*-(4-methylphenethyl)quinazolin-4-amine (15e):** column chromatography on silica gel (hexane/ethyl acetate, 3/7) followed by crystallization in methanol* at 0 °C, white solid, yield: 96% (*80%), m.p. = 182 °C. HPLC: 99.93% at 254 nm (t_R = 14.01 min). FT-IR (ATR cm^{-1}): 1350 (C-N), 1573-1618 (C=$N_{quinazoline}$), 2918 (-CH_2-), 3018-3048 (C-H_{arom}), 3217 (N-H). ^1H NMR (400 MHz, DMSO-d_6) δ ppm: 2.25 (s, 3H, CH_3), 2.93 (t, *J* = 7.6 Hz, 2H, CH_2), 3.74 (q, *J* = 7.1 Hz, 2H, CH_2), 7.09 (d, *J* = 7.8 Hz, 2H, CH_{arom}), 7.15 (d, *J* = 7.8 Hz, 2H, CH_{arom}), 7.49 (t, *J* = 7.5 Hz, 1H, CH_{arom}), 7.68 (d, *J* = 8.2 Hz, 1H, CH_{arom}), 7.70 – 7.79 (m, 1H, CH_{arom}), 8.21 (d, *J* = 8.3 Hz, 1H), 8.33 (s, 1H, NH), 8.49 (s, 1H, $CH_{pyrimidine}$). ^{13}C (100 MHz, DMSO-d_6) δ ppm: 20.50, 34.00, 42.09, 114.92, 122.48, 125.38, 127.40, 128.43, 128.81, 132.28, 134.88, 136.34, 149.03, 155.06, 159.22. HRMS (ESI): *m/z* calc. for $C_{17}H_{18}N_3$ $[M + H]^+$: 264.1495; found, 264.1503.

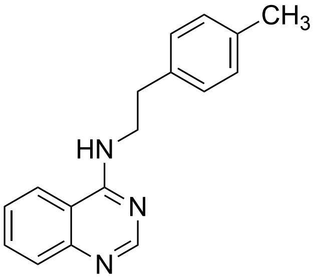

15e

***N*-(4-ethylphenethyl)quinazolin-4-amine (16e):** column chromatography on silica gel (ethyl acetate, 100%) followed by crystallization in 2-propanol:water* (1:1), grey solid, yield: 98% (*72%),

m.p. = 160-161 °C. HPLC: 99.53% at 254 nm (t_R = 14.56 min). FT-IR (ATR cm^{-1}): 1350 (C-N), 1573-1618 (C=N$_{quinazoline}$), 2922-2960 (-CH_2-), 3222 (N-H). ^{1}H NMR (400 MHz, $CDCl_3$) δ ppm: 1.24 (t, J = 7.7 Hz, 3H, CH_3), 2.64 (q, J = 7.7 Hz, 2H, CH_2), 3.00 (t, J = 6.9 Hz, 2H, CH_2), 3.92 (q, J = 6.5 Hz, 2H, CH_2), 5.92 (s, 1H, NH), 7.17 (s, 4H, CH$_{arom}$), 7.41 (t, J = 7.7 Hz, 1H, CH$_{arom}$), 7.56 (d, J = 8.3 Hz, 1H, CH$_{arom}$), 7.70 (t, J = 7.7 Hz, 1H, CH$_{arom}$), 7.82 (d, J = 8.4 Hz, 1H, CH$_{arom}$), 8.67 (s, 1H, CH$_{pyrimidine}$). ^{13}C (100 MHz, $CDCl_3$) δ ppm: 15.57, 28.48, 34.87, 42.36, 115.07, 120.40, 125.93, 128.28, 128.63, 128.81, 132.49, 136.03, 142.68, 149.50, 155.50, 159.43. HRMS (ESI): *m/z* calc. for $C_{18}H_{20}N_3$ [M + H]$^+$: 278.1652; found, 278.1663.

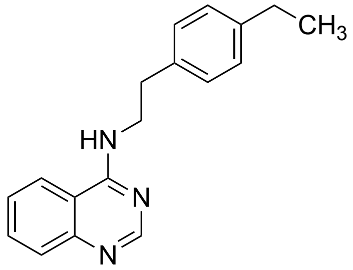

16e

***N*-(2-(naphthalen-2-yl)ethyl)quinazolin-4-amine (17e):** column chromatography on silica gel (hexane/ethyl acetate, 2/8) followed by crystallization in 2-propanol*, white solid, yield: 86% (*78%), m.p. = 168 °C. HPLC: 99.90% at 254 nm (t_R = 14.68 min). FT-IR (ATR cm^{-1}): 1347 (C-N), 1575-1617 (C=N$_{quinazoline}$), 2931-2961 (-CH_2-), 3054 (C-H$_{arom}$), 3227 (N-H). ^{1}H NMR (400 MHz, DMSO-d_6) δ ppm: 3.15 (t, J = 7.5 Hz, 2H, CH_2), 3.88 (q, J = 6.4 Hz, 2H, CH_2), 7.42 – 7.51 (m, 4H, CH$_{arom}$), 7.69 (d, J = 8.2 Hz, 1H, CH$_{arom}$), 7.73 – 7.78 (m, 2H, CH$_{arom}$), 7.82 – 7.88 (m, 3H, CH$_{arom}$), 8.21 (d, J = 8.3 Hz, 1H, CH$_{arom}$), 8.42 (s, 1H, NH), 8.52 (s, 1H, CH$_{pyrimidine}$). ^{13}C (100 MHz, DMSO-d_6) δ ppm: 34.56, 41.87, 114.93, 122.48, 125.20, 125.41, 125.86, 126.56, 127.22, 127.34, 127.42, 127.44, 127.66, 131.64, 132.31, 133.09, 137.14, 149.05, 155.06, 159.27. HRMS (ESI): *m/z* calc. for $C_{20}H_{18}N_3$ [M + H]$^+$: 300.1495; found, 300.1497.

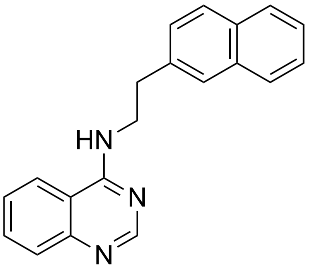

17e

***N*-(3-phenylpropyl)quinazolin-4-amine (18e):** column chromatography on silica gel (ethyl acetate, 100%), white solid, yield: 96%, m.p. = 112 °C. HPLC: 99.90% at 254 nm (t_R = 13.97 min). FT-IR (ATR cm^{-1}): 1352 (C-N), 1574-1617 ($C{=}N_{quinazoline}$), 2931 ($-CH_2-$), 3019 ($C-H_{arom}$), 3217 (N-H). ^1H NMR (400 MHz, $CDCl_3$) δ ppm: 2.09 (p, *J* = 7.3 Hz, 2H, CH_2), 2.78 (t, *J* = 7.5 Hz, 2H, CH_2), 3.72 (q, *J* = 6.6 Hz, 2H, CH_2), 5.81 (s, 1H, NH), 7.18 – 7.31 (m, 5H, CH_{arom}), 7.39 (t, *J* = 7.6 Hz, 1H, CH_{arom}), 7.48 (d, *J* = 8.3 Hz, 1H, CH_{arom}), 7.69 (t, *J* = 7.8 Hz, 1H, CH_{arom}), 7.81 (d, *J* = 8.4 Hz, 1H, CH_{arom}), 8.66 (s, 1H, $CH_{pyrimidine}$). ^{13}C (100 MHz, $CDCl_3$) δ ppm: 30.65, 33.69, 41.23, 114.98, 120.46, 125.82, 126.15, 128.43, 128.57, 128.65, 132.46, 141.52, 149.44, 155.45, 159.45. HRMS (ESI): *m/z* calc. for $C_{17}H_{18}N_3$ $[M + H]^+$: 264.1495; found, 264.1493.

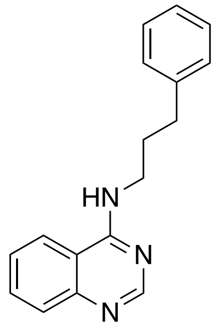

18e

1.3. Biological assays

1.3.1. Determination of minimum inhibitory concentration (ATCC 27294)

The synthesized compounds were evaluated for their ability to inhibit the growth of *M. tuberculosis* H37Rv strain (ATCC 27294) using the colorimetric resazurin reduction microplate assay (REMA) to determine the minimum inhibitory concentration (MIC).[3] The MIC represents the lowest concentration of the compound that completely prevents visible growth of the bacillus. Isoniazid **19** (INH) and rifampicin **20** (RIF) were the drug controls in this assay. The stock solutions for all compounds were prepared at 2 mg/mL in DMSO (Sigma-Aldrich) and stored at −20 °C. Working solutions were freshly prepared at the time of the experiment, diluting the compounds in Difco™ Middlebrook 7H9 broth (Becton, Dickinson and Company – BD) supplemented with 10% BBL™ Middlebrook ADC enrichment (albumin, dextrose, and catalase – BD) with DMSO at a final concentration of 5% at the solubility limit of each compound. The tested concentrations (µg/mL) were determined based on solubility tests: visual inspection (precipitates) followed by microscopic analysis (crystal formation observation). For each compound, we performed 10-point 2-fold serial dilutions in 100 µL of Middlebrook 7H9 medium with 10% Middlebrook ADC enrichment directly in 96-well plates. Mtb was cultured in Middlebrook 7H9 broth containing 10% BD Difco™ BBL™ Middlebrook OADC enrichment (oleic acid, albumin, dextrose, catalase), 0.05% Tween 80 (Sigma-Aldrich), and 0.2% glycerol (Sigma-Aldrich) at 37 °C to an optical density at 600 nm (OD_{600}) of 0.6 to 0.8. Then, cultures were suspended by vortexing with sterile glass beads (1 mm) for 3 min and left to rest for 15 min. The supernatant was measured at OD_{600}, and aliquots were stored at −80 °C. Bacterial cultures were then diluted to a theoretical OD_{600} of 0.006 in Middlebrook 7H9 broth supplemented with 10% Middlebrook ADC enrichment and transferred to 96-well plates (100 µL/well). The final DMSO concentration in the assay was 2.5%. Growth controls without drugs and sterility controls without mycobacteria were included on each plate. The plates were covered, sealed with parafilm, and incubated at 37 °C for 7 days. A 60 µL solution of 0.01% resazurin (Sigma-Aldrich) was added to each well of the plate before a final incubation step at 37 °C for 48 h. The MIC values were obtained by determining the lowest compound concentration at which no color change from blue (growth inhibition) to pink (indicating growth) was observed. Three independent experiments were performed, and MIC values were considered the most frequent compound concentration that inhibited the visible growth of bacilli.

1.3.2. Determination of minimum inhibitory concentration (ATCC 25618)

The *M. tuberculosis* H37Rv-LP (ATCC 25618) constitutively expressing codon-optimized DsRed (DREAM8)[4] was cultured at 37 °C in Middlebrook 7H9 medium (Difco™) supplemented with 10% v/v oleic acid, albumin, dextrose, and catalase (OADC) (BD), and 0.05% w/v Tween 80 (7H9-Tw-OADC). Assay plates were prepared using a Multidrop Combi (model: 5840300) for medium addition and a VIAFLO Assist Plus (Integra) for adding controls and compounds. The compounds were tested in 10-point 2-fold serial dilutions starting at 100 µM. The final DMSO concentration in the assay was 1%. Growth was measured by optical density (OD) at 590 nm and relative fluorescence intensity (RFU) after 5 days of culture.[5] Plates were read using a Synergy H4 plate reader (BioTek). The MIC was defined as the compound concentration required to inhibit 90% of bacillary growth. Rifampicin **20** (maximum inhibition) controls and a rifampicin **20** dose-response curve were included on each plate. Bacterial viability was measured in logarithmic phase cells (OD_{590} = 0.4 – 0.8).

1.3.3. Determination of minimum inhibitory concentration in the mutant strains LP-0106898-RM1 (QcrB T313I) and LP-0497754-RM301 (MmpL3) of *M. tuberculosis*

The *M. tuberculosis* LP-0106898-RM1 (QcrB T313I) and LP-0497754-RM301 (MmpL3) mutant strains were cultured at 37 °C in Middlebrook 7H9 medium (Difco™) supplemented with 10% v/v oleic acid, albumin, dextrose, and catalase (OADC) (BD), and 0.05% w/v Tween 80 (7H9-Tw-OADC). H37Rv-LP (ATCC 25618) is the parental strain. Assay plates were prepared using a Multidrop Combi (model: 5840300) for medium addition and a VIAFLO Assist Plus (Integra) for adding controls and compounds. The compounds were tested in 10-point 2-fold serial dilutions starting at 100 µM. The final DMSO concentration in the assay was 1%. Growth was measured by optical density at 590 nm after 5 days of culture. Plates were read using a Synergy H4 microplate reader (BioTek). The MIC was defined as the compound concentration required to inhibit 90% of bacillary growth. Rifampicin **20** (maximum inhibition) controls and a rifampicin **20** dose-response curve were included on each plate. Bacterial viability was measured in logarithmic phase cells (OD_{590} = 0.4 – 0.8).

1.3.4. Intracellular activity

THP-1 cells (ATCC TIB-202) were propagated in RPMI 1640 medium, diluted to 106106 cells/mL, and treated with 80 nM PMA overnight. The cells were infected with *M. tuberculosis* H37Rv-LP expressing DsRed[4] (DREAM8) at a multiplicity of infection (MOI) of 0.1 for 24 hours, followed by the removal of extracellular bacteria through washing. The infected cells were

collected using Accutase/EDTA, dispensed into plates at 2.5×1032.5×103 cells/well, and incubated with the compounds for 72 hours. THP-1 nuclei were stained with SYBR Green I, and the plates were imaged using an ImageXpress Micro High Content Screening System (Molecular Devices) with a 4x objective and FITC and Texas Red channels. Image analysis was performed using MetaXpress software. The integrated intensity of Mtb or macrophages was calculated for each well. Growth inhibition was determined for each test well by normalizing to the mean integrated intensity of DMSO control wells. Isoniazid **19** (INH) and staurosporine **21** (STA) were included as controls for antitubercular activity and cytotoxicity, respectively. The TC_{50} was defined as the compound concentration required to reduce macrophage viability by 50%. The IC_{50} and IC_{90} were defined as the compound concentration required to reduce bacterial growth by 50% and 90%, respectively.[6]

1.3.5. ATP depletion assay

The *M. tuberculosis* H37Rv-LP (ATCC 25618) strain was cultured in duplicate plates (one for OD and one for RLU) at 37 °C in Middlebrook 7H9 medium (Difco™) supplemented with 10% v/v oleic acid, albumin, dextrose, and catalase (OADC) (BD) and 0.05% w/v Tween 80 (7H9-Tw-OADC). ATP levels (RLU) were measured using the BacTiter-Glo® Assay Kit (Promega), following the manufacturer's instructions. The OD_{590} and RLU of the plates were recorded using a Synergy H4 microplate reader (BioTek). ATP/OD was calculated by dividing the relative luminescence units (RLU) from the BacTiter-Glo® plate by the OD_{590} readings. Q203 **22** (telacebec) was used as positive control.

1.3.6. Reactive Oxygen Species (ROS) assay

An H37Rv-LP (ATCC 25618) culture at OD_{590} = 1.0 was harvested, washed, and resuspended in 7H9-Tw containing 40 µM 2',7'-dichlorofluorescein diacetate (DCFDA). The bacteria were incubated at 37 °C for 30 minutes, then harvested, washed, and resuspended in 7H9-Tw. DCFDA-labeled cells were dispensed into black-walled, clear-bottom 96-well plates containing the test compounds in serial dilutions in 7H9-Tw. DMSO was used as the vehicle control. Fluorescence (RFU) (Ex_{485}/Em_{535}) was measured using a Synergy H4 microplate reader (BioTek). Econazole **23** was included as a dose-response control.

1.3.7. Membrane potential ($\Delta\psi$) assay

H37Rv-LP (ATCC 25618) cultures were grown in 7H9-Tw-OADC to an OD_{590} = 1.0 and resuspended in fresh 7H9-Tw. The cells were incubated with 15 µM DiOC2 at room temperature for 20 minutes. The Mtb cultures were washed and added to black-walled 96-well plates containing serial dilutions of the test compounds and incubated for 15 minutes at room temperature. Carbonyl cyanide 3-chlorophenylhydrazone **24** (CCCP) (Sigma-Aldrich) was used as a positive control for membrane depolarization, while DMSO served as a vehicle control. A Synergy H4 microplate reader (BioTek) was used to measure green fluorescence (RFU) (Ex_{488} nm/Em_{530} nm) and shifts to red fluorescence (Ex_{488} nm/Em_{650} nm). Membrane potential ($\Delta\psi$) was calculated as the ratio of red to green fluorescence.

1.3.8. Antimicrobial spectrum assays

Specific media were used for each strain evaluated. Assay plates were prepared using a Multidrop Combi (model: 5840300) for medium addition and a VIAFLO Assist Plus (Integra) for adding controls and compounds. The compounds were tested in 10-point 2-fold serial dilutions starting at 100 µM. The final DMSO concentration in the assay was 1%. MIC determination for *Mycobacterium smegmatis* was performed using the REMA (resazurin reduction microplate assay) method.

1.3.8.1. Minimum inhibitory concentration determination in *Mycobacterium avium* smooth (S) and rough (R) strains

Mycobacterium avium strains (2285S and 2285R) were cultured at 37 °C in Middlebrook 7H9 medium (Difco™) supplemented with 10% v/v oleic acid, albumin, dextrose, catalase supplement (OADC) (BD), and 0.05% w/v Tween 80 (7H9-Tw-OADC). After 5 days of incubation, a 20% alamarBlue® solution was added to all plate wells. Following 1 day of incubation, growth was assessed by measuring relative fluorescence intensity (RFU) (Ex_{590} nm; Em_{560} nm) using a Synergy H1 plate reader (BioTek). MIC was defined as the compound concentration required to inhibit 90% of bacillus growth. Clarithromycin **25** (CLA) (maximum inhibition control) and a clarithromycin **25** dose-response curve were included on each plate. Bacterial viability was measured in log-phase cells (OD_{590} = 0.4 – 0.8).

1.3.8.2. Minimum inhibitory concentration determination in *Mycobacterium abscessus*

The *Mycobacterium abscessus* strain 103R was cultured at 37 °C in Middlebrook 7H9 medium (Difco™) supplemented with 10% v/v oleic acid, albumin, dextrose, catalase supplement (OADC) (BD), and 0.05% w/v Tween 80 (7H9-Tw-OADC). After 3 days of incubation, growth was measured at OD_{590} using a Synergy H1 plate reader (BioTek). MIC was defined as the compound concentration required to inhibit 90% of bacillus growth. Clarithromycin **25** (CLA) (maximum inhibition control) and a clarithromycin **25** dose-response curve were included on each plate. Bacterial viability was measured in log-phase cells (OD_{590} = 0.4 – 0.8).

1.3.8.3. Minimum Inhibitory Concentration (MIC) Determination in *Staphylococcus aureus*

The *Staphylococcus aureus* (ATCC 29213) strain was cultured at 37 °C in Cation-Adjusted Mueller-Hinton Broth (CAMHB). After 1 day of incubation, growth was measured at OD_{590} using a Synergy H1 plate reader (BioTek). MIC was defined as the compound concentration required to inhibit 90% of bacterial growth. Vancomycin **26** (VAN) (maximum inhibition control) and a vancomycin **26** dose-response curve were included on each plate.

1.3.8.4. Minimum Inhibitory Concentration (MIC) Determination in *Escherichia coli*

The *Escherichia coli* strain RFM 795 was cultured at 37 °C in M9 medium. After 1 day of incubation, growth was measured at OD_{590} using a Synergy H1 plate reader (BioTek). MIC was defined as the compound concentration required to inhibit 90% of bacterial growth. Ampicillin **27** (AMP) (maximum inhibition control) and an ampicillin **27** dose-response curve were included on each plate.

1.3.8.5. Minimum Inhibitory Concentration (MIC) Determination in *Mycobacterium smegmatis*

Mycobacterium smegmatis mc^2155 was cultured at 37 °C in Middlebrook 7H9 medium supplemented with 10% v/v ADC and 0.05% w/v Tween 80. MIC values were determined in 96-well plates by 2-fold serial dilution of compounds prepared in 5% DMSO, with a final volume of 200 µL per well. A standardized inoculum (OD_{600} = 0.001) was added to each well, and plates were incubated at 37 °C for 24 h. After incubation, 60 µL of 0.01% resazurin solution was added, and plates were incubated for an additional 24 h. Color change from blue to pink indicated bacterial viability. Rifampicin was used as a positive control for growth inhibition in all experiments. Three independent

experiments were performed, and MIC values were considered the most frequent compound concentration that inhibited the visible growth of bacilli.

1.3.9. Cell viability evaluation

Cell viability assays were performed using three methods: the MTT method (colorimetric assay based on 3-(4,5-dimethylthiazol-2-yl)-2,5-diphenyltetrazolium bromide), the neutral red (NR) incorporation assay, and ATP measurement using the CellTiter-Glo® Luminescent Cell Viability Assay.

1.3.9.1. MTT assay

Compounds were incubated with HepG2 and Vero cells in 96-well plates at three concentrations: 20 µM, 5 µM, and 1 µM. The culture medium was replaced with fresh medium containing the test compounds, and cells were incubated for 72 hours. After incubation, 40 µL of MTT reagent diluted in PBS was added to the cells, followed by a further 3-hour incubation. Then, 100 µL of DMSO was added to dissolve the formazan crystals. Absorbance was measured at 570 nm using a 96-well plate reader (Spectramax M2, Molecular Devices). The measured absorbance was considered linearly proportional to the number of viable cells with functional mitochondria.[7]

1.3.9.2. Neutral Red (NR) assay

Cells were seeded in 96-well plates in complete medium and incubated for 24 hours at 37 °C with 5% CO_2 to allow for growth. The compounds were then incubated with HepG2 and Vero cells at three concentrations: 20 µM, 5 µM, and 1 µM, for 72 hours at 37 °C with 5% CO_2. After incubation, the treatment medium was removed, and 200 µL of neutral red dye (25 µg/mL) in serum-free medium was added. Cells were incubated with the dye for 3 hours at 37 °C with 5% CO_2. Following incubation, cells were washed with PBS, and 100 µL of desorption solution (ethanol:acetic acid:water, 50:1:49) was added. Cells were gently shaken for 30 minutes until complete dissolution. Absorbance was measured at 562 nm using a plate spectrophotometer. Cell viability was expressed as a percentage relative to the untreated control.[8]

1.3.9.3. Cell viability determination by luminescence

The cytotoxicity of the compounds was assessed by measuring the viability of HepG2 cells grown in glucose or galactose medium for 72 hours in the presence of the compounds.[9] The

derivatives were prepared using serial dilutions in DMSO. The highest tested compound concentration was 100 µM, with compounds being soluble in DMSO at 10 mM. HepG2 cells were cultured in complete DMEM (formulation: DMEM, 1X penicillin-streptomycin solution, 2 mM Corning® Glutagro supplement, 1 mM sodium pyruvate, 10% v/v fetal bovine serum), seeded in 384-well assay plates, and incubated for 24 hours at 37°C with 5% CO_2. The compounds were then added using a Digital Dispenser (D300e, Tecan), and cells were incubated for an additional 72 hours. The final concentration of DMSO was 1%. Cell viability was determined using the CellTiter-Glo® Luminescent Cell Viability Assay (Promega) by measuring relative luminescent units (RLU). The dose-response curve was fitted using the Levenberg-Marquardt algorithm. The CC_{50} was defined as the compound concentration that induced a 50% reduction in viable cells. Each experiment included staurosporine **21** (STA) as a control.

1.4. *In silico* methods

1.4.1. Density prediction

The ChemBCPP server (http://chembcpp.scbdd.com/) was used to predict the density of reagents for which this information was not provided by the manufacturer or could not be found in the literature (*e.g.*, **8e**).[10]

1.4.2. Determination of cLog*P*

The calculated Log*P* ($\text{Log}P_{oct}/_{H2O}$) was determined using the ChemDraw® software (version 20.0.0.38). This calculation is based on the fragment method. For further details regarding the algorithm used in the $\text{Log}P_{oct}/_{H2O}$ prediction, refer to reference.[11]

1.5. Microsomal stability

Test compound (6 µM) was incubated at 37 °C with male BALB/c mouse liver microsomes or mixed gender human liver microsomes (Xenotech LLC; 0.5 mg/mL, 0.1 mM potassium phosphate buffer, pH 7.4) and the reaction started with addition of excess NADPH (2 mM, pH 7.4). Samples (35 µL) were removed at time 0, 5, 10, 20, 30, and 60 min and mixed with 35 µL of acetonitrile, methanol, water solution (2:1:1) containing 6 µL internal standard terfenadine and 0.05% *v*/*v* acetic acid. The samples were centrifuged at 4000 rpm for 10 min and the supernatant was analyzed using a 1100 HPLC and Single Quad 6120 (Agilent, USA). Compounds were tested in duplicate, and an exponential decay curve fit was used on the ratio of peak area of test compound to internal standard at each time point to calculate the rate constant (k). The rate of intrinsic clearance (CL_{int}) of each test compound was then calculated using the following equation: CL_{int} (mL/min/mg) = k x V where V was mL/mg protein. Verapamil (6 µM) was used as a positive control. Incubation of each compound with microsomes in the absence of NADPH and analyzed at 0 and 60 min was used as the negative control. The human biological samples were sourced ethically, and their research use was in accord with the terms of the informed consents.

2. Results

2.1. Determination of minimum inhibitory concentration against H37Rv-LP (ATCC 25618) and MmpL3 triple mutant (F255L, V646M, F644I)

Table S1. *In vitro* activity against *M. tuberculosis* H37Rv-LP (ATCC 25618) and MmpL3 triple mutant (F255L, V646M, F644I)

Entry	H37Rv-LP (μM)	MmpL3 (F255L, V646M, F644I) (μM)
**13c**	3.4	5.4
**16c**	2.0	3.4
**17c**	2.5	2.6
**9d**	0.95	0.89
**10d**	0.92	1.5
**11d**	0.84	1.0
**13d**	3.7	8.3
**15d**	0.48	0.89
**16d**	1.9	2.3
**17d**	1.4	1.4
**18a**	1.6	1.2
**18b**	1.5	1.5
**18c**	0.28	<0.19
**18d**	0.58	0.64
**18e**	24.0	39.0
**20 (RIF)**	0.030	0.030

2.2. ATP depletion assay

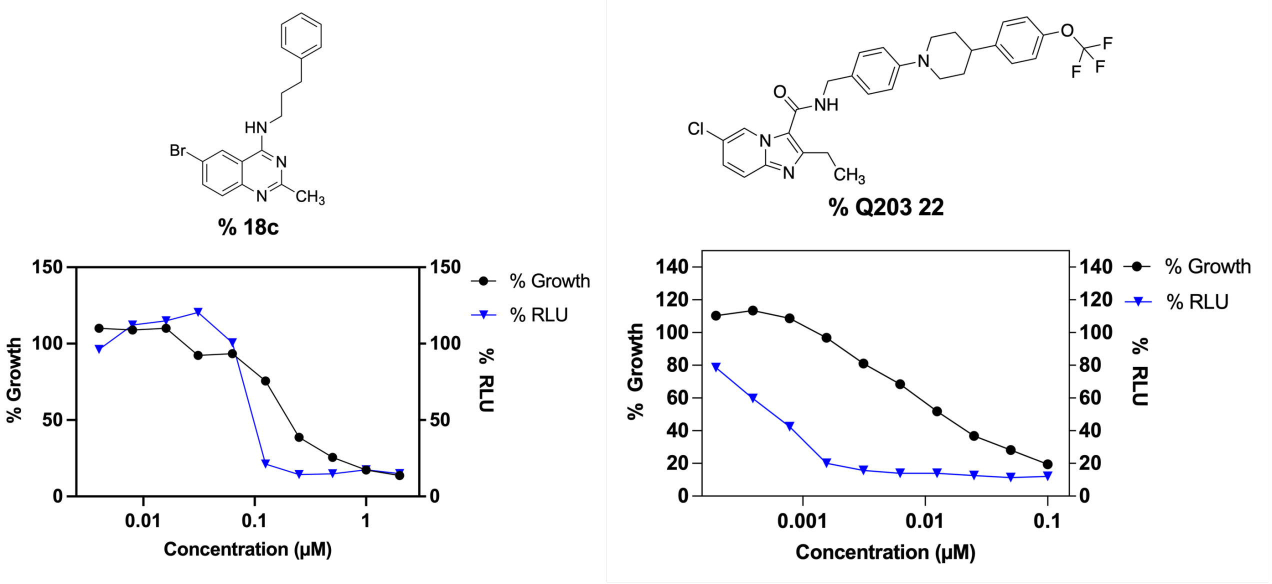

Figure S1. ATP depletion assay with compound **18c** as a representative, showing a similar trend to the other selected molecules.

2.3. Reactive Oxygen Species (ROS) assay

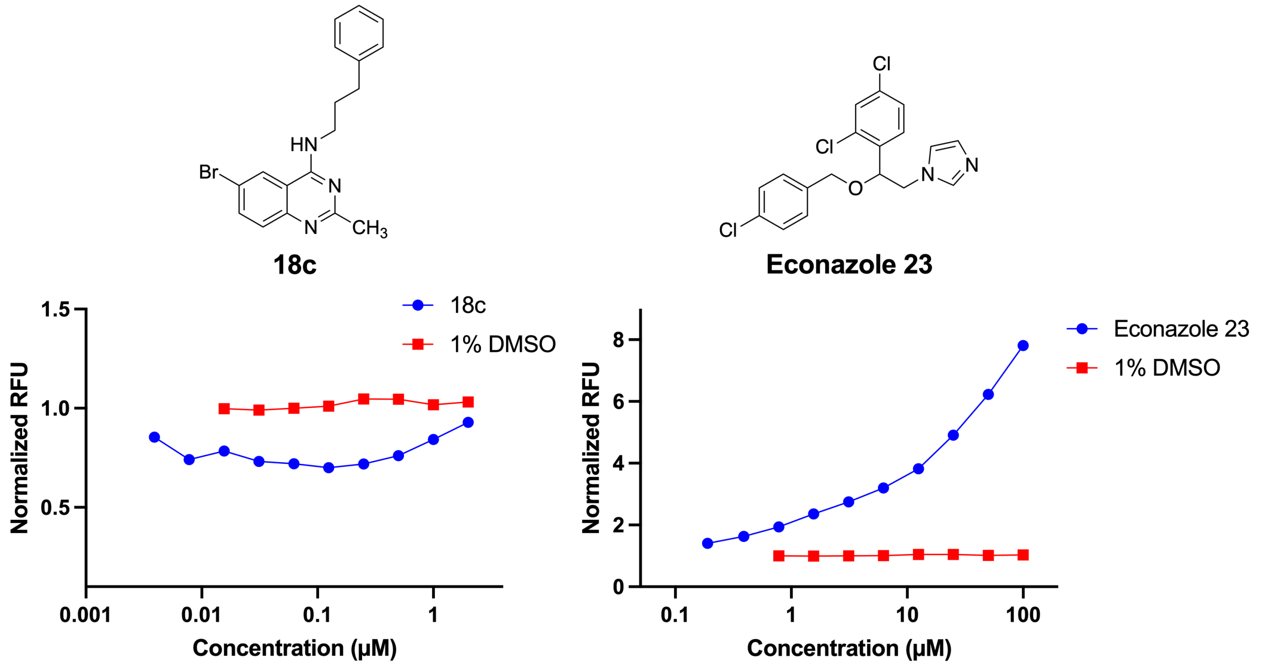

Figure S2. Reactive Oxygen Species (ROS) assay showing compound **18c** as a representative, showing a similar trend to the other selected molecules.

2.4. Membrane potential (Δψ) assay

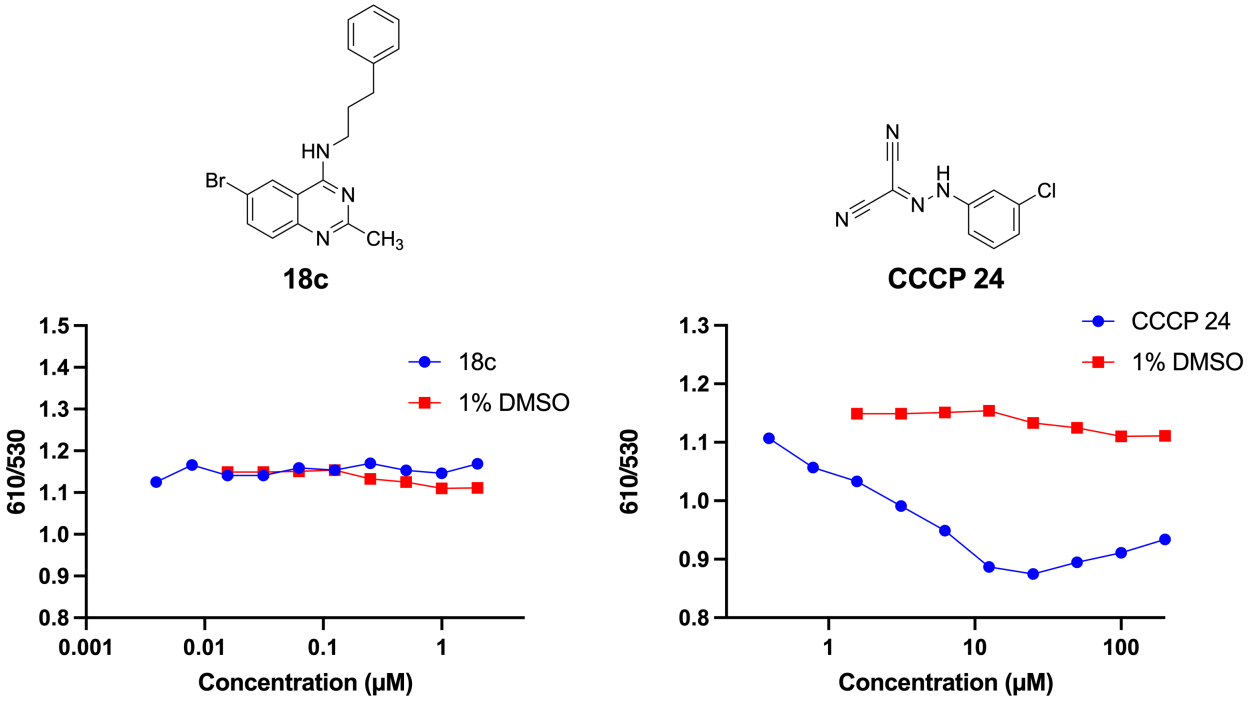

Figure S3. Membrane potential (Δψ) assay showing compound **18c** as a representative, showing a similar trend to the other selected molecules.

2.5. Antimicrobial spectrum assays

Table S2. Antimicrobial spectrum assays

	CIM (μM)					
**Entry**	**29213**[a]	**RFM 795**[b]	**103R**[c]	**2285S**[d]	**2285R**[e]	**mc^{2}155**[f]
**18b**	>100	54	>100	>100	>100	>32.5
**18c**	>100	>100	>100	>100	>100	>56.1
**18d**	>100	>100	>100	>100	>100	>12.4
**20 (RIF)**	-	-	-	-	-	48.6
**25 (CLA)**	-	-	0.5	0.4	1.4	-
**26 (VAN)**	2.9	-	-	-	-	-
**27 (AMP)**	-	12.0	-	-	-	-

[a]*Staphylococcus aureus* (ATCC 29213). [b]*Escherichia coli* RFM 795. [c]*Mycobacterium abscessus* 103R. [d]*Mycobacterium avium* 2285S (smooth). [e]*Mycobacterium avium* 2285R (rough). [f]*Mycobacterium smegmatis* mc^{2}155.

2.6. Microsomal stability

Table S3. Microsomal stability determination using mouse liver microsomes (MLM) and human liver microsomes (HLM).

		**18b**	**18c**	**18d**
**Clearance**	**MLM (µL/min/mg)** **[% remaining]; $t_{1/2}$ (min)**	530 [7][2]; 2.6	354 [2][3]; 3.9	202 [63][1]; 6.9
	**HLM (µL/min/mg)** **[% remaining]; $t_{1/2}$ (min)**	252 [8][3]; 5.5	184 [6][4]; 7.5	243 [9][3]; 5.7

[1] at 5 min; [2] at 10 min; [3] at 20 min; [4] at 30 min. Reference clearance values (µL/min/mg): MLM (low: <13.1; high: >71); HLM (low: <8.6; high: >47).

2.7. Cell viability evaluation

Table S4. Cell viability determination using the CellTiter-Glo® Luminescent Cell Viability Assay.

Entry	MIC (µM)[a]	CC_{50} HepG2+Glu (µM)[b]	CC_{50} HepG2+Gal (µM)[b]	SI HepG2+Glu[c]	SI HepG2+Gal[c]
**13c**	3.4	1.5	1.0	0.4	0.3
**16c**	2.0	4.0	1.6	2.0	0.8
**17c**	2.5	4.6	1.8	1.8	0.7
**9d**	0.95	10.9	5.1	11.5	5.4
**10d**	0.92	10.8	6.4	11.7	7.0
**11d**	0.84	4.2	3.3	5.0	3.9
**13d**	3.7	3.4	2.2	0.9	0.6
**15d**	0.48	11.4	6.6	23.8	13.8
**16d**	1.9	7.4	5.3	3.9	2.8
**17d**	1.4	7.1	5.2	5.1	3.7
**18a**	1.6	7.7	2.6	4.8	1.6
**18b**	1.5	13.9	11.0	9.3	7.3
**18c**	0.28	19.1	9.7	68.2	34.6
**18d**	0.58	20.3	13.0	35.0	22.4
**18e**	24.0	10.9	2.5	0.5	0.1
**21 (STA)**[d]	-	0.030	0.040	-	-

[a]H37Rv-LP (ATCC 25618). [b]The cytotoxicity and selectivity index (SI) of the compounds were evaluated in HepG2 supplemented with glucose (Glu) and galactose (Gal). Cell viability was measured using the CellTiter-Glo® Luminescent Cell Viability Assay, with results expressed as the concentration required to reduce viability by 50% (CC_{50}) compared to untreated control. [c]Selectivity index (SI = $CC_{50}/MIC_{H37Rv\text{-}LP}$). [d]Staurosporine **23** (STA).

3. ^{1}H and ^{13}C NMR spectra of the synthesized compounds

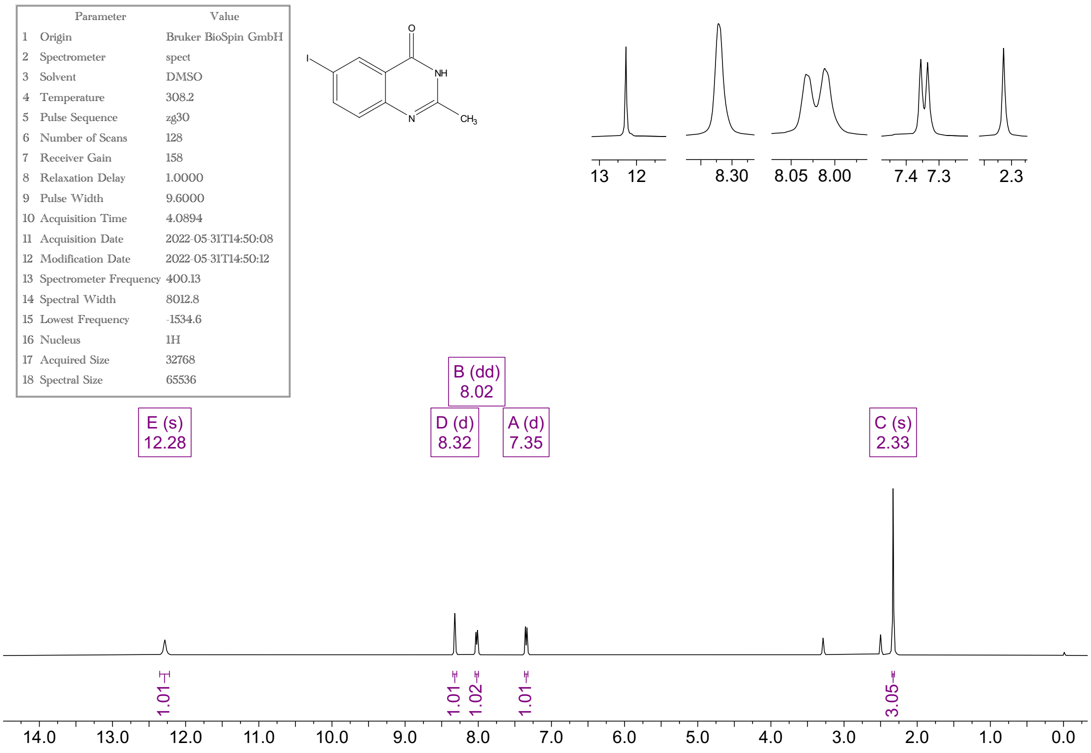

Figure S4. ^{1}H NMR spectrum (400 MHz) of compound **7d** in DMSO-d$_6$.

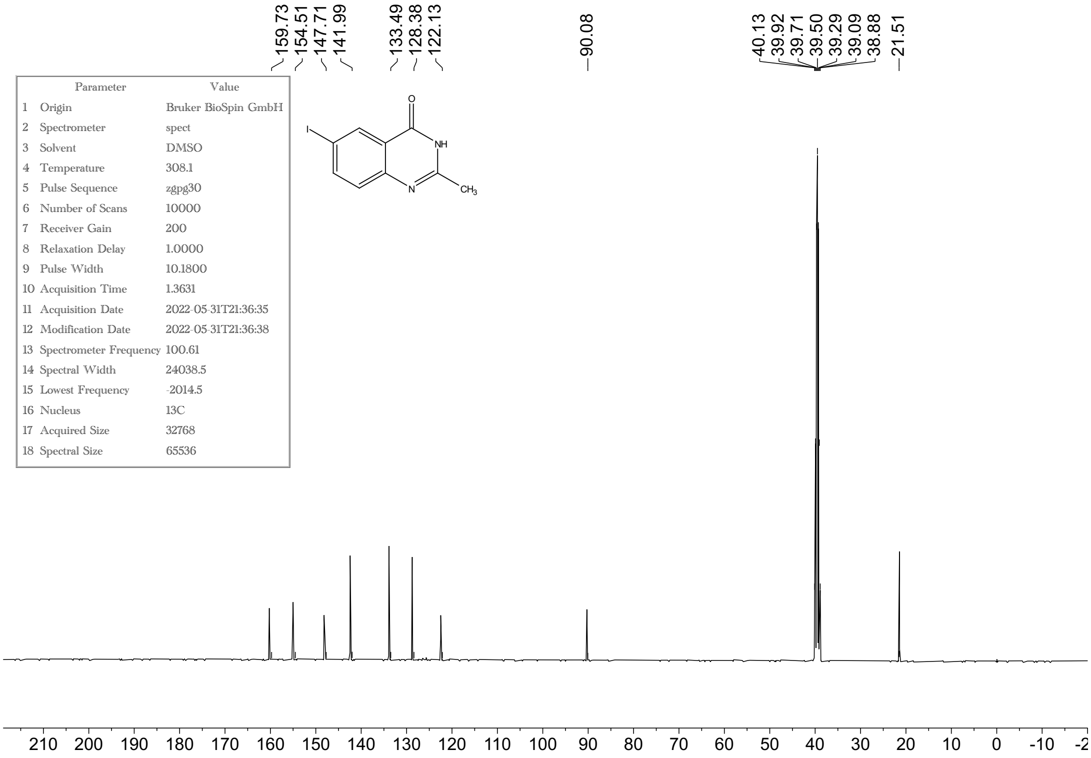

Figure S5. ^{13}C NMR spectrum (100 MHz) of compound **7d** in DMSO-d$_6$.

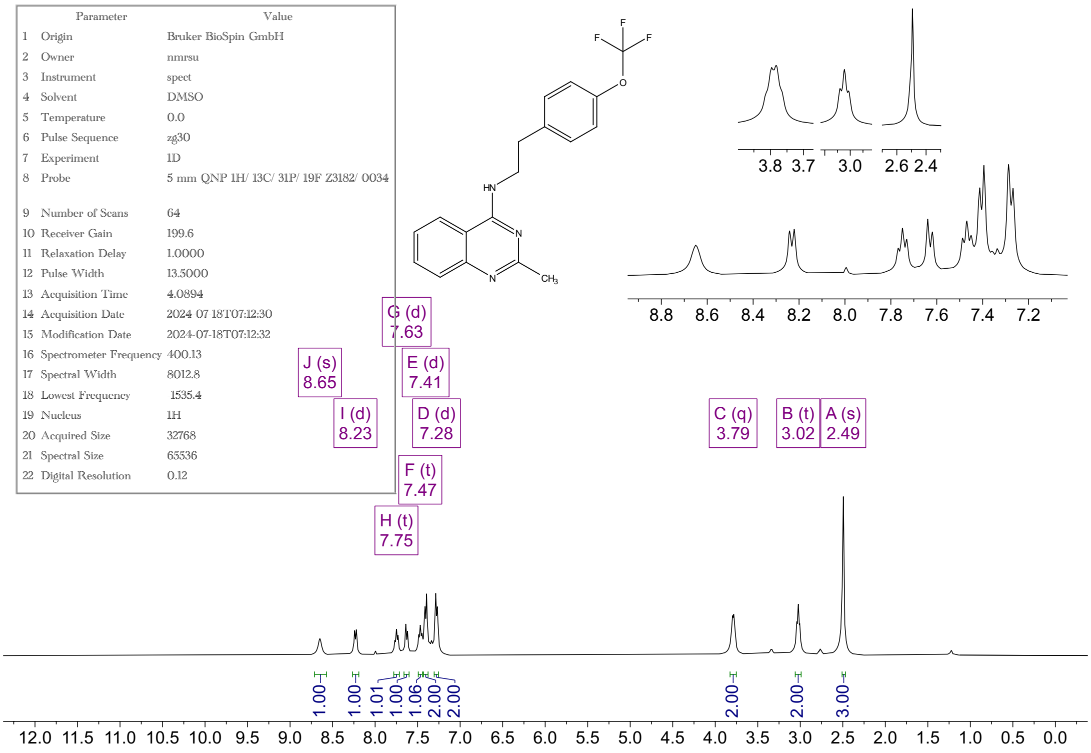

Figure S6. ^{1}H NMR spectrum (400 MHz) of compound **13a** in DMSO-d$_6$.

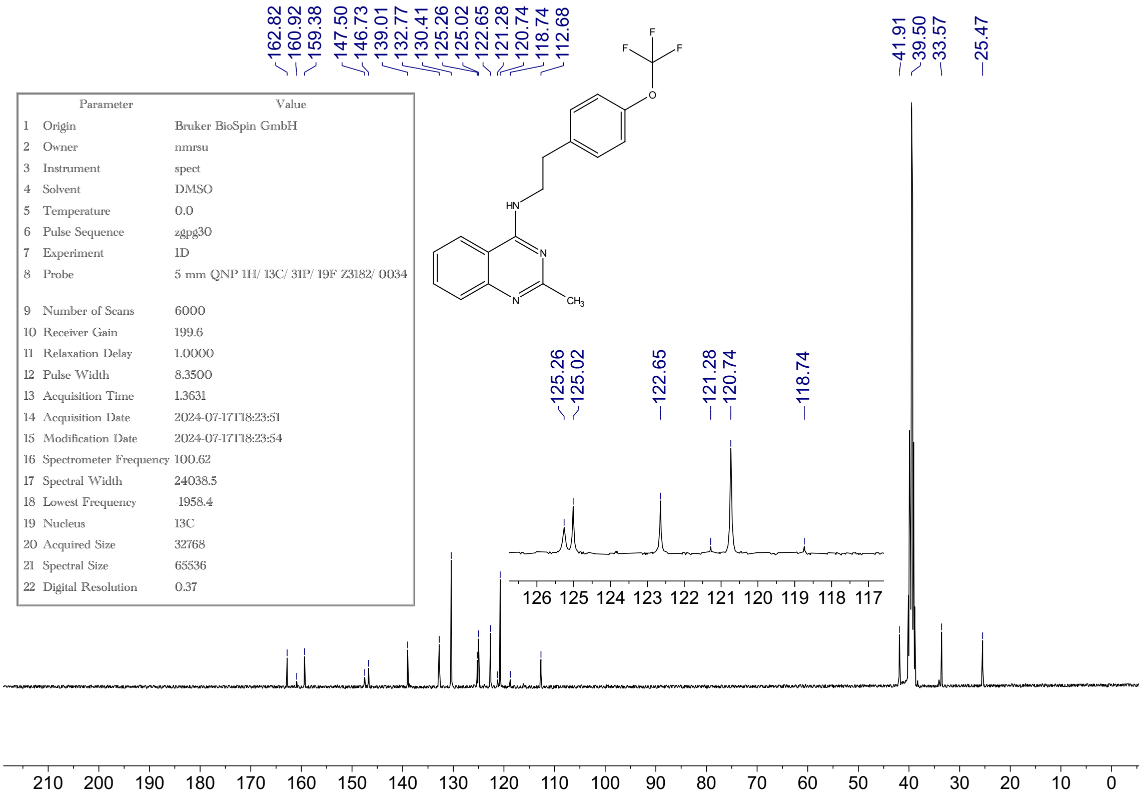

Figure S7. ^{13}C NMR spectrum (100 MHz) of compound **13a** in DMSO-d$_6$.

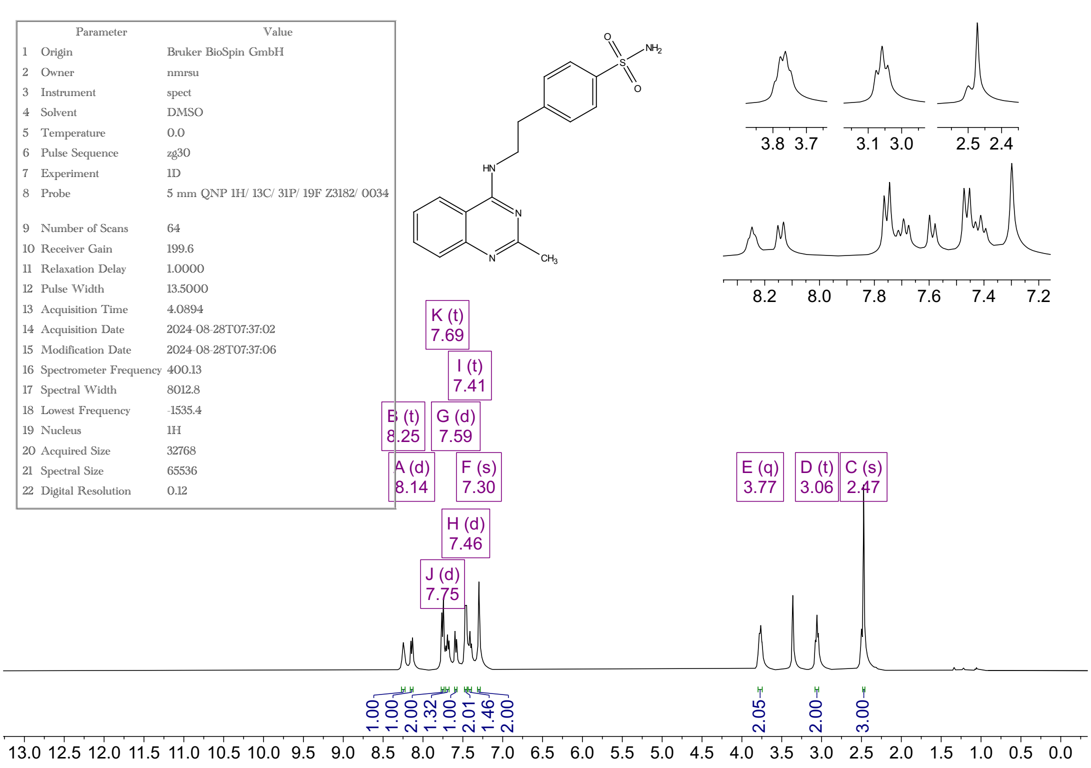

Figure S8. ^{1}H NMR spectrum (400 MHz) of compound **14a** in DMSO-d$_6$.

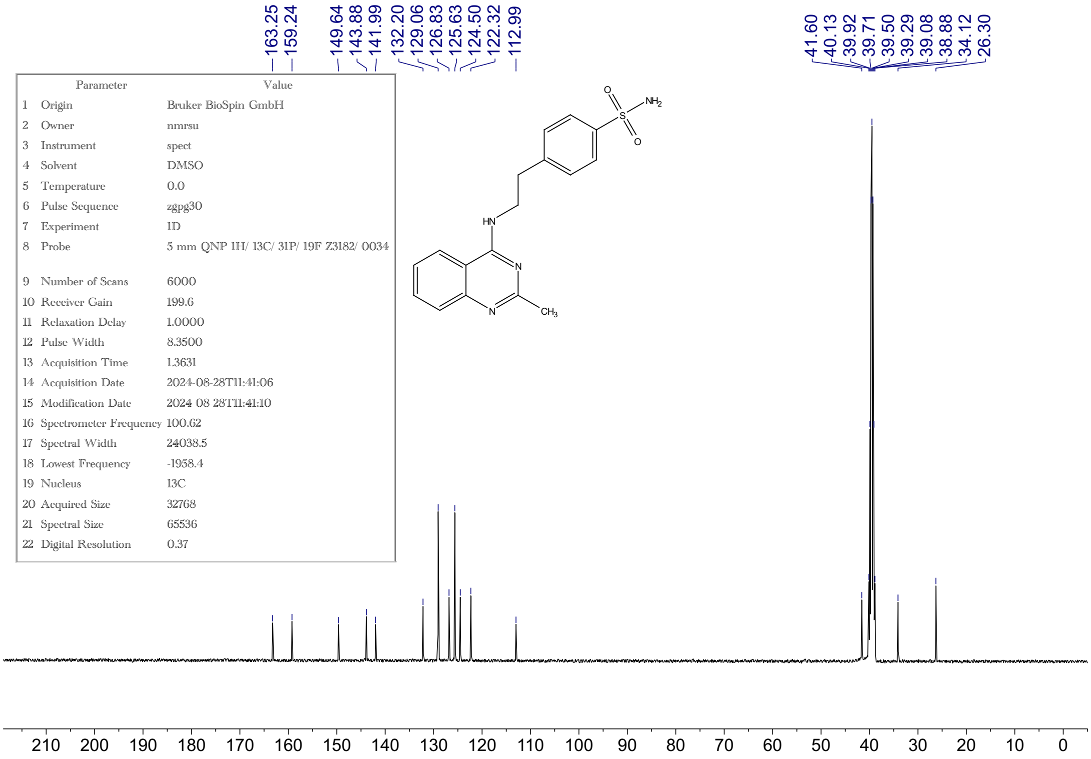

Figure S9. ^{13}C NMR spectrum (100 MHz) of compound **14a** in DMSO-d$_6$.

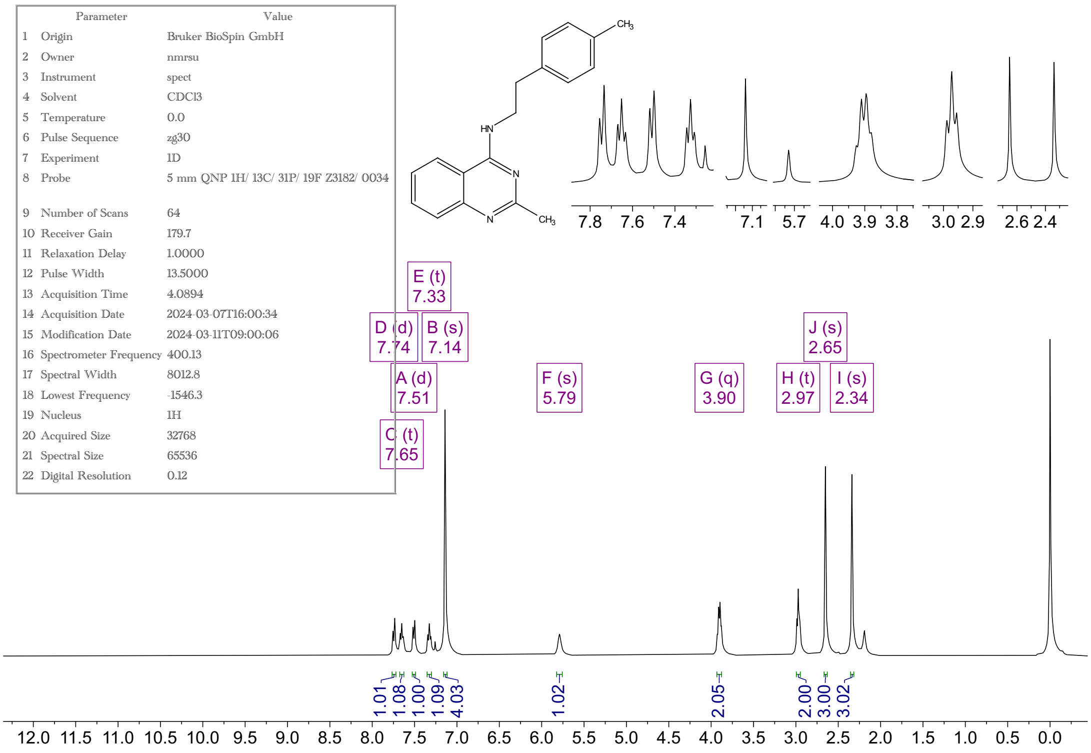

Figure S10. ^{1}H NMR spectrum (400 MHz) of compound **15a** in $CDCl_3$.

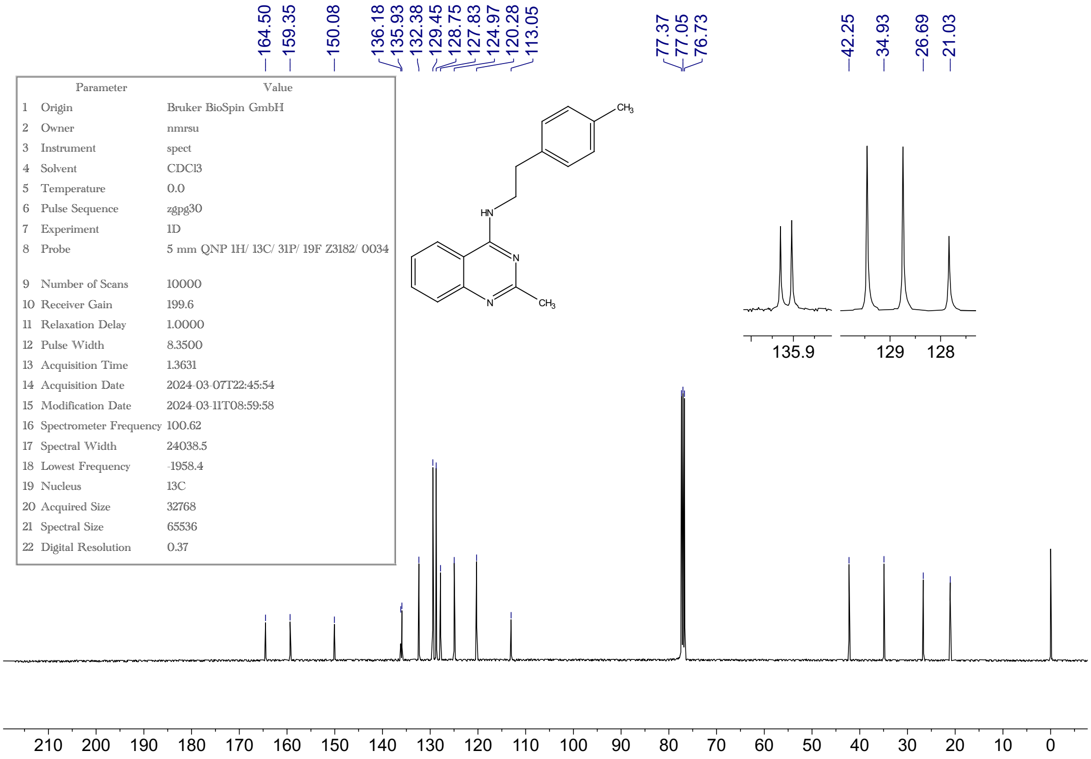

Figure S11. ^{13}C NMR spectrum (100 MHz) of compound **15a** in $CDCl_3$.

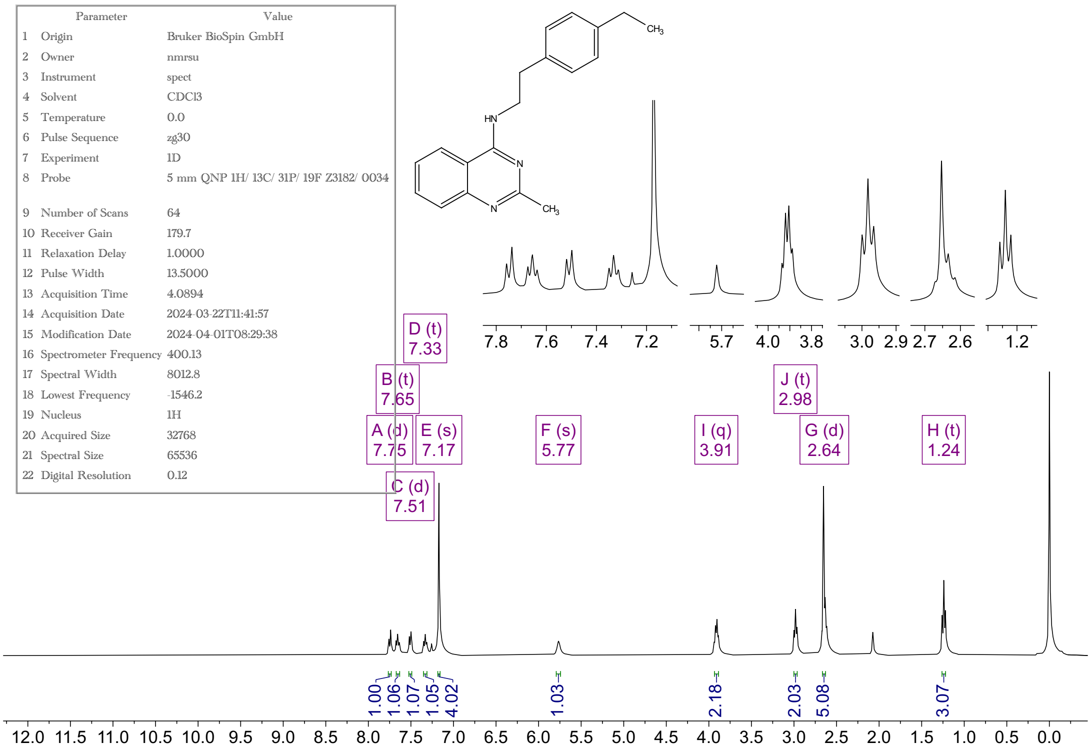

Figure S12. ^{1}H NMR spectrum (400 MHz) of compound **16a** in $CDCl_3$.

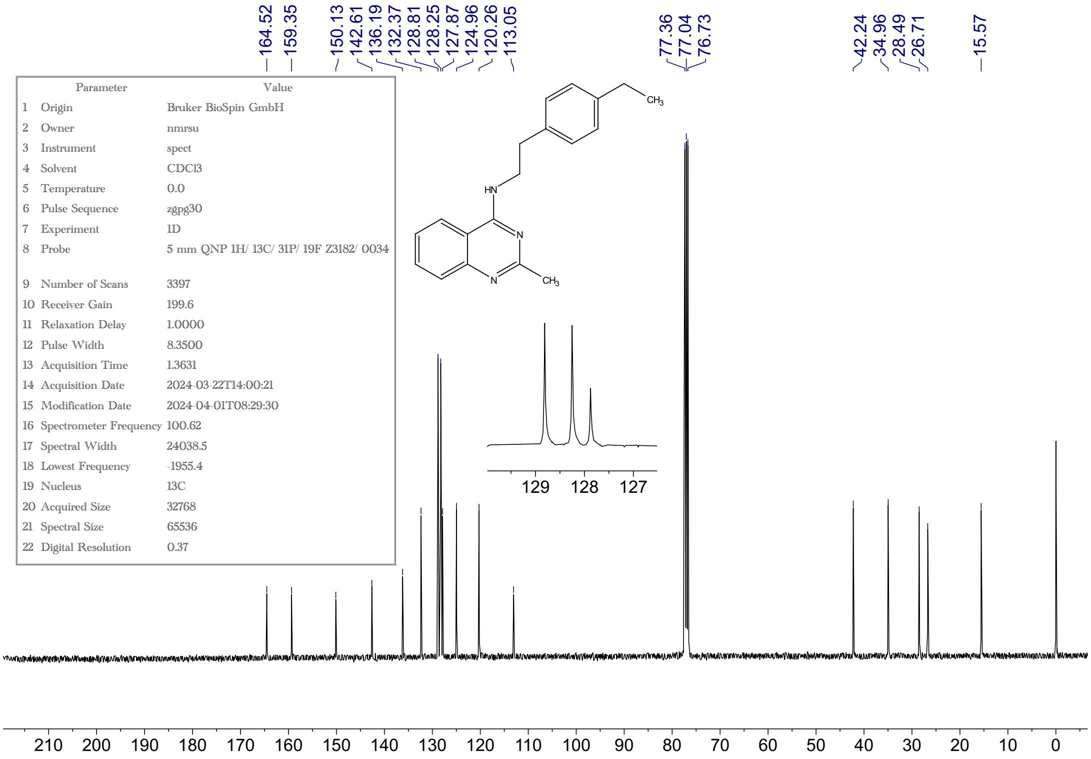

Figure S13. ^{13}C NMR spectrum (100 MHz) of compound **16a** in $CDCl_3$.

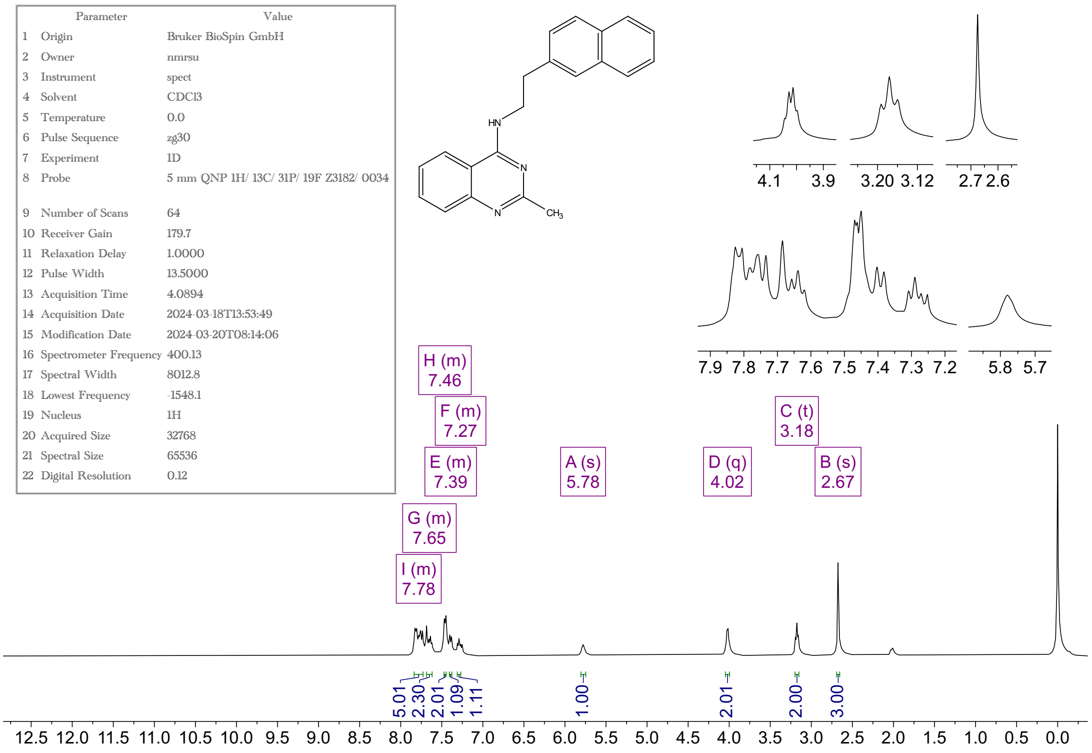

Figure S14. ^{1}H NMR spectrum (400 MHz) of compound **17a** in $CDCl_3$.

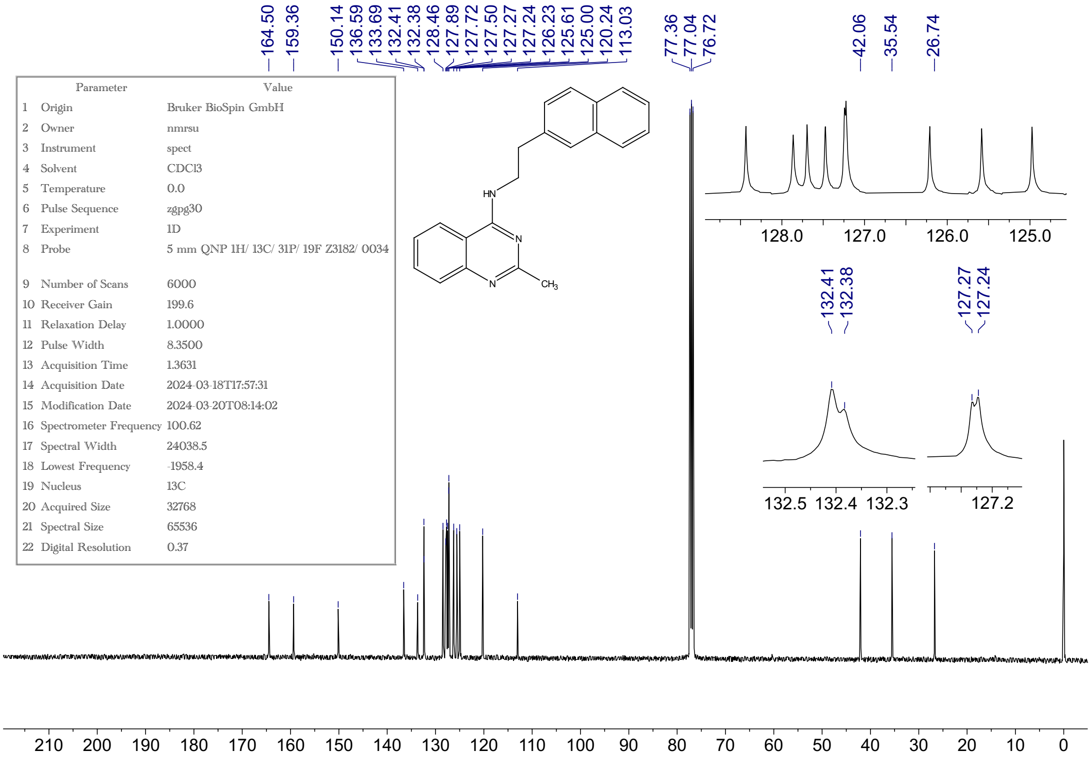

Figure S15. ^{13}C NMR spectrum (100 MHz) of compound **17a** in $CDCl_3$.

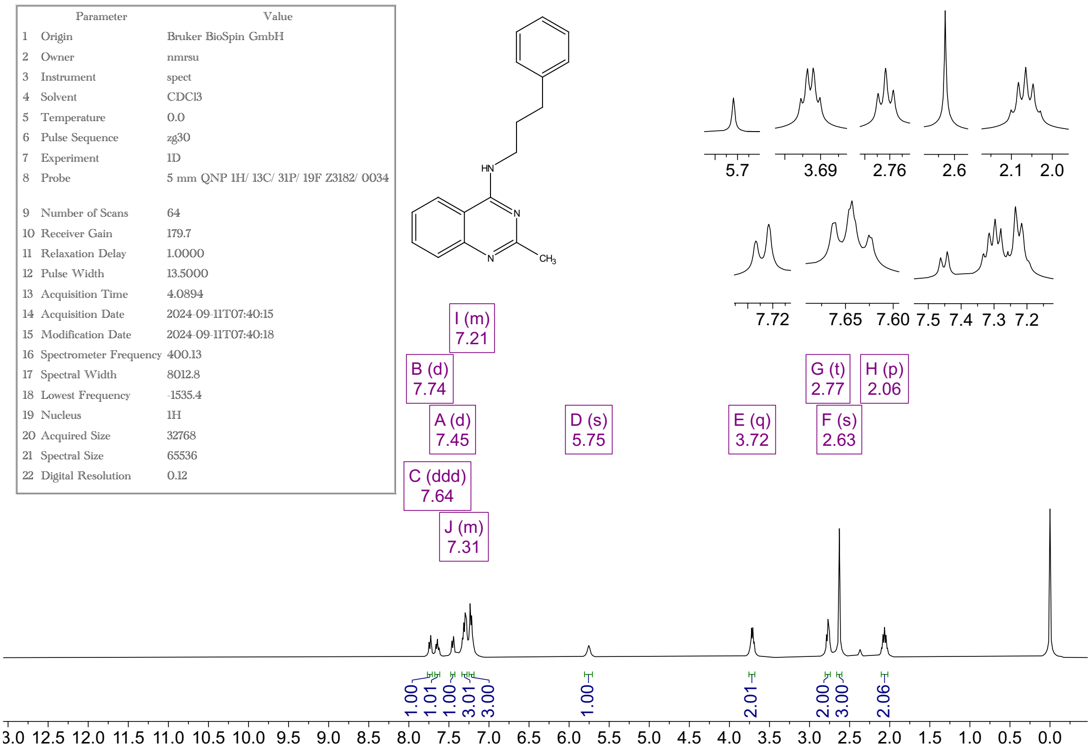

Figure **S16**. ^{1}H NMR spectrum (400 MHz) of compound **18a** in $CDCl_3$.

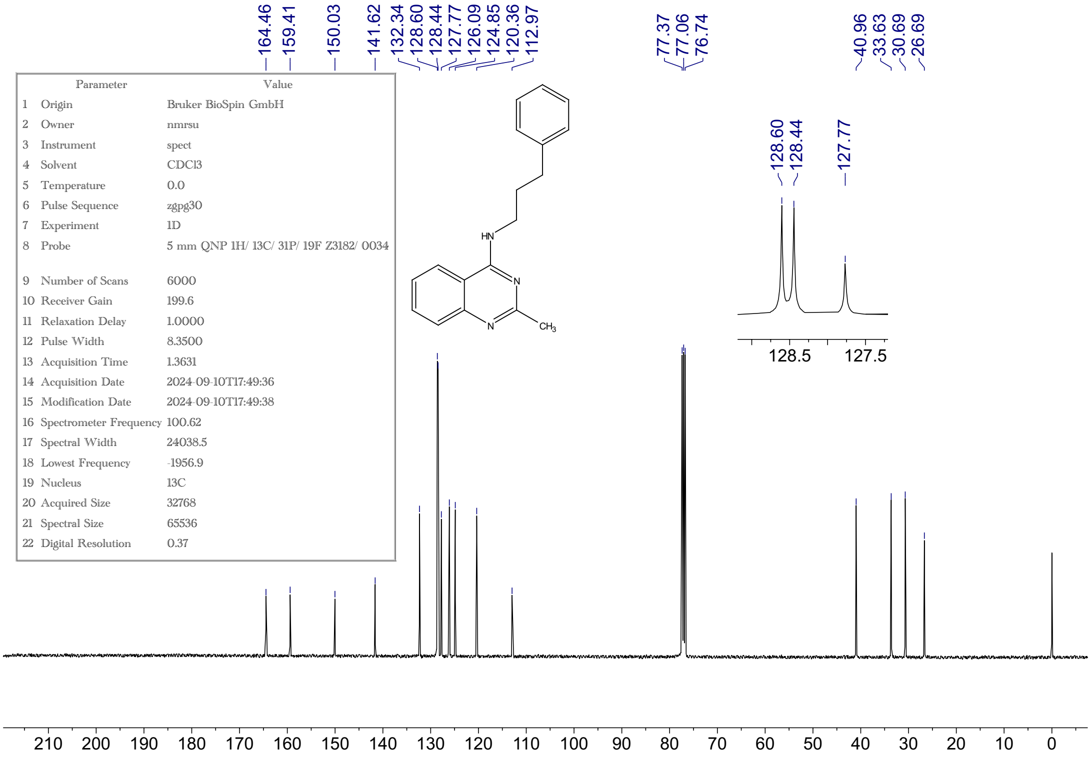

Figure S17. ^{13}C NMR spectrum (100 MHz) of compound **18a** in $CDCl_3$.

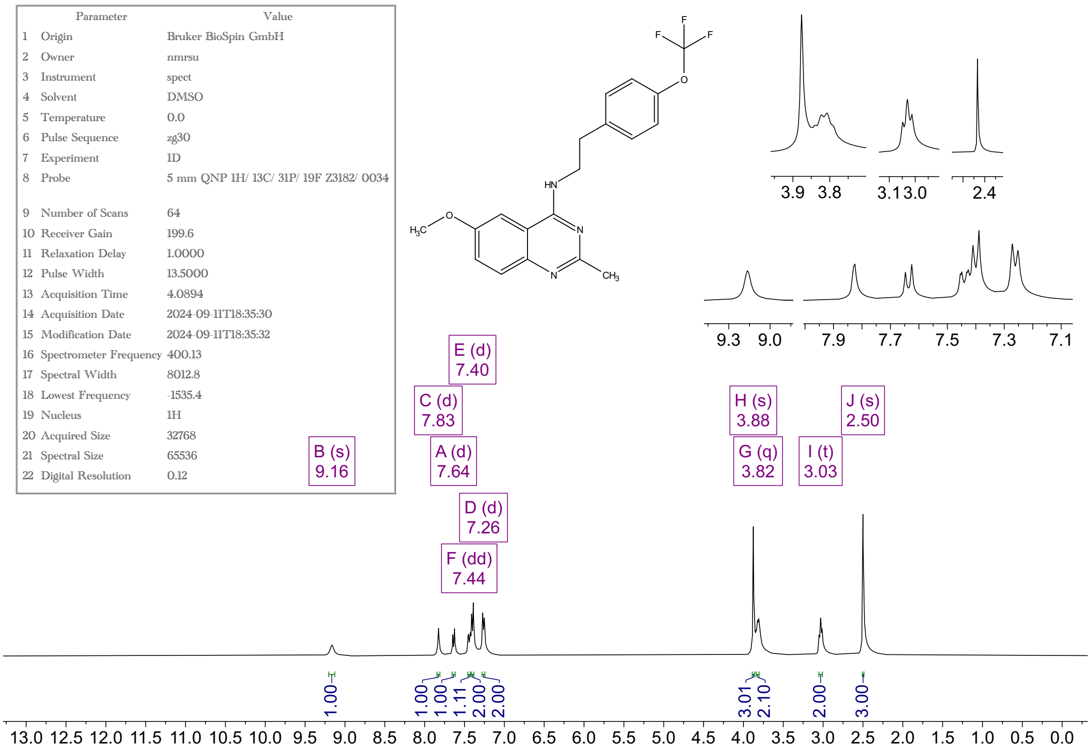

Figure S18. ^{1}H NMR spectrum (400 MHz) of compound **13b** in DMSO-d_6.

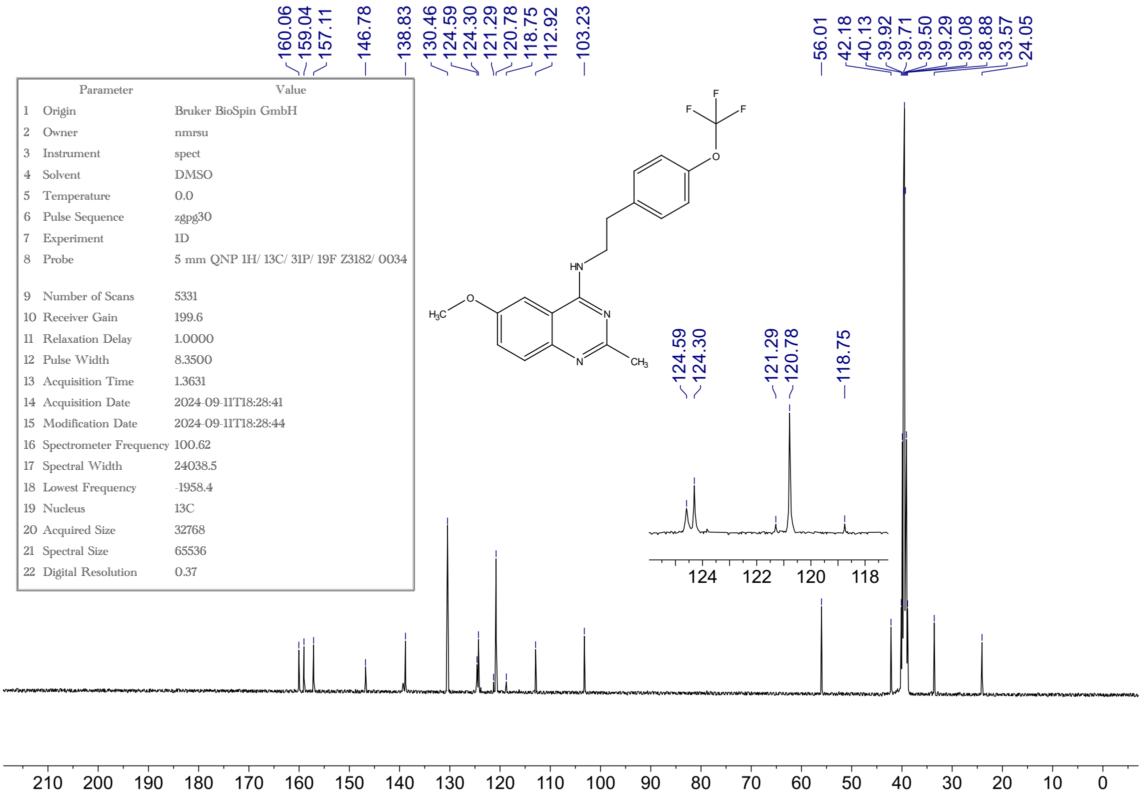

Figure S19. ^{13}C NMR spectrum (100 MHz) of compound **13b** in DMSO-d_6.

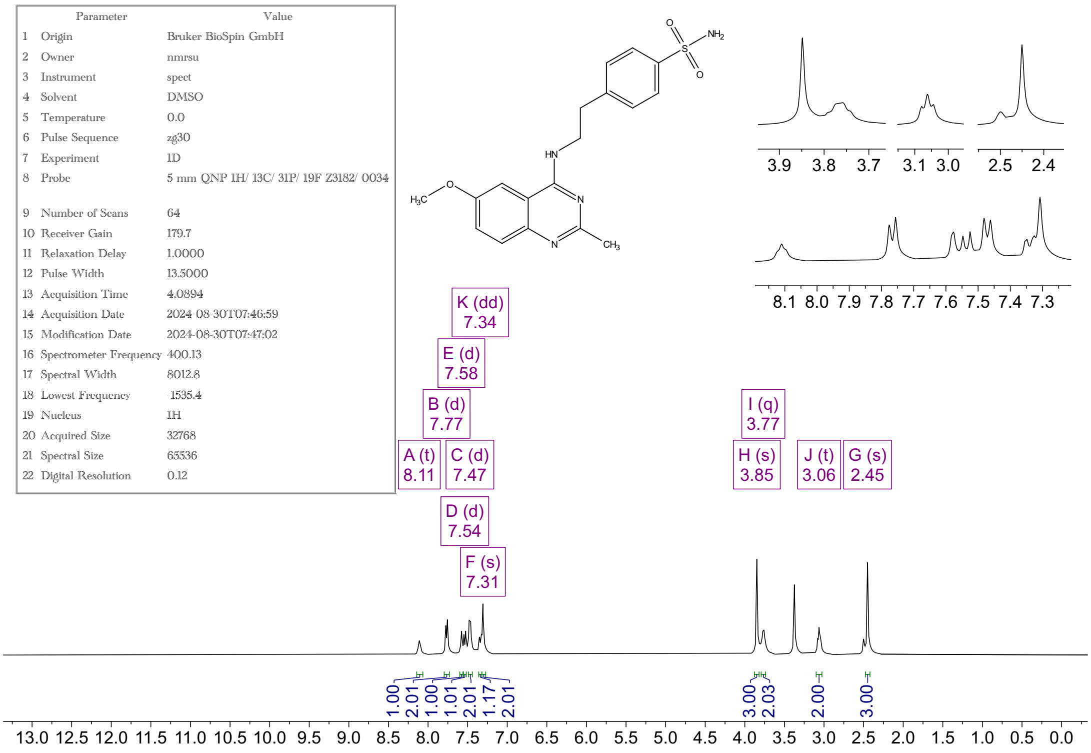

Figure S20. ^{1}H NMR spectrum (400 MHz) of compound **14b** in DMSO-d$_6$.

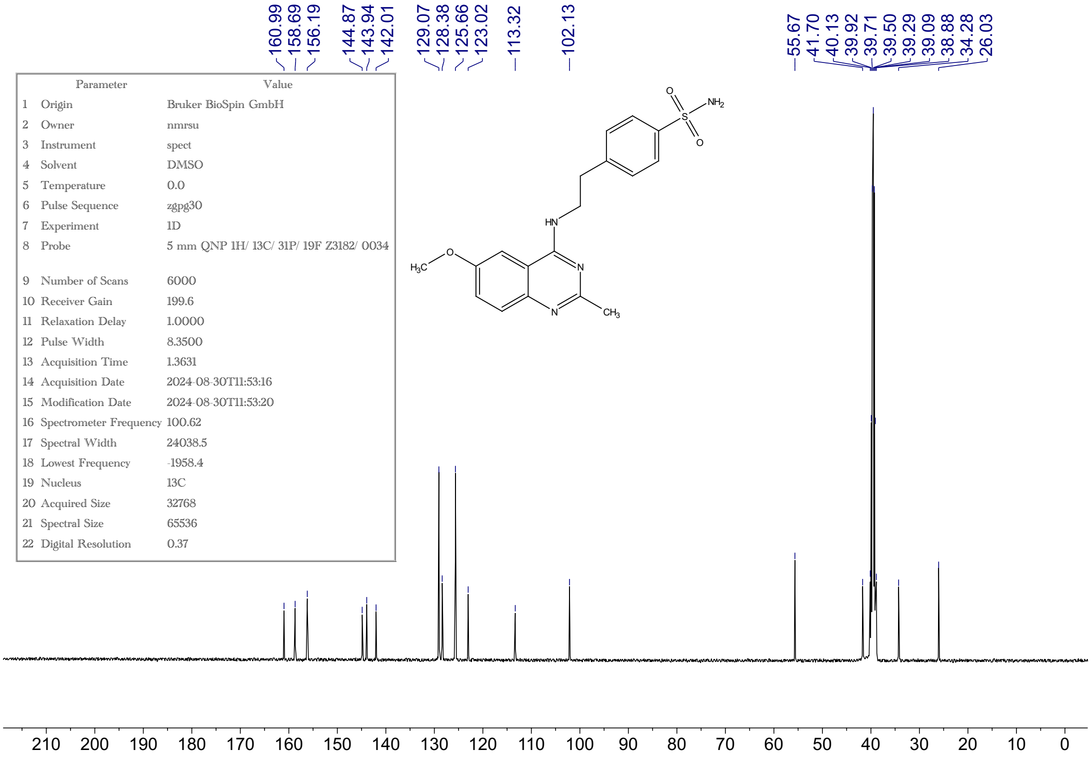

Figure S21. ^{13}C NMR spectrum (100 MHz) of compound **14b** in DMSO-d$_6$.

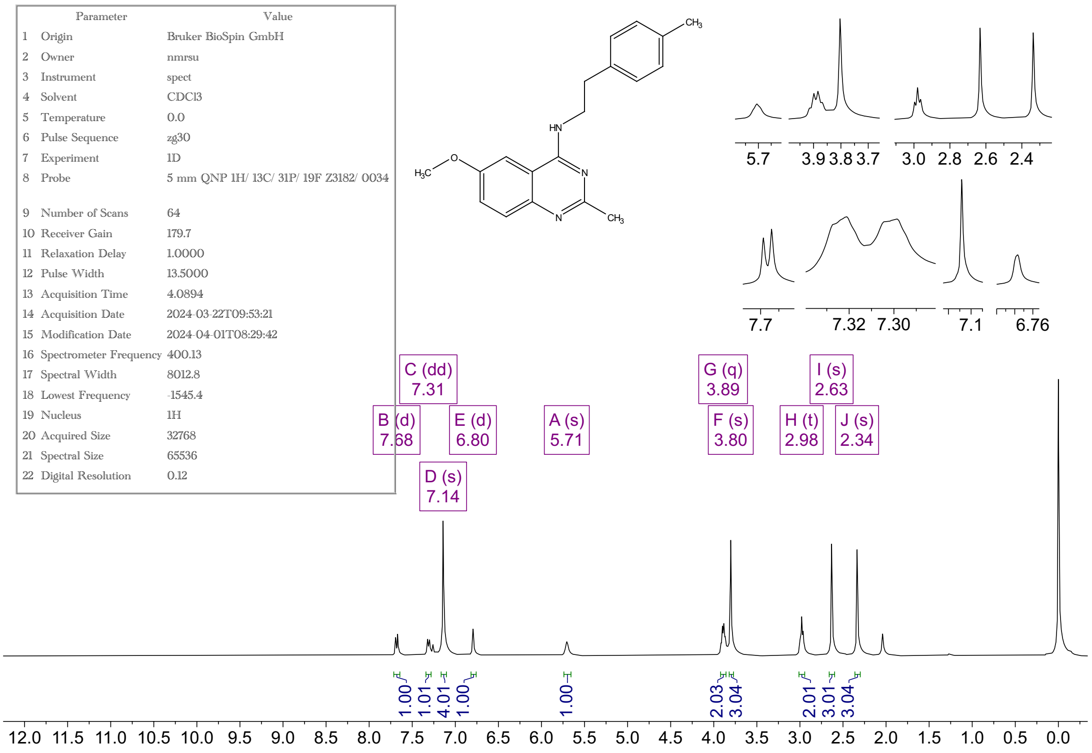

Figure S22. ^{1}H NMR spectrum (400 MHz) of compound **15b** in $CDCl_3$.

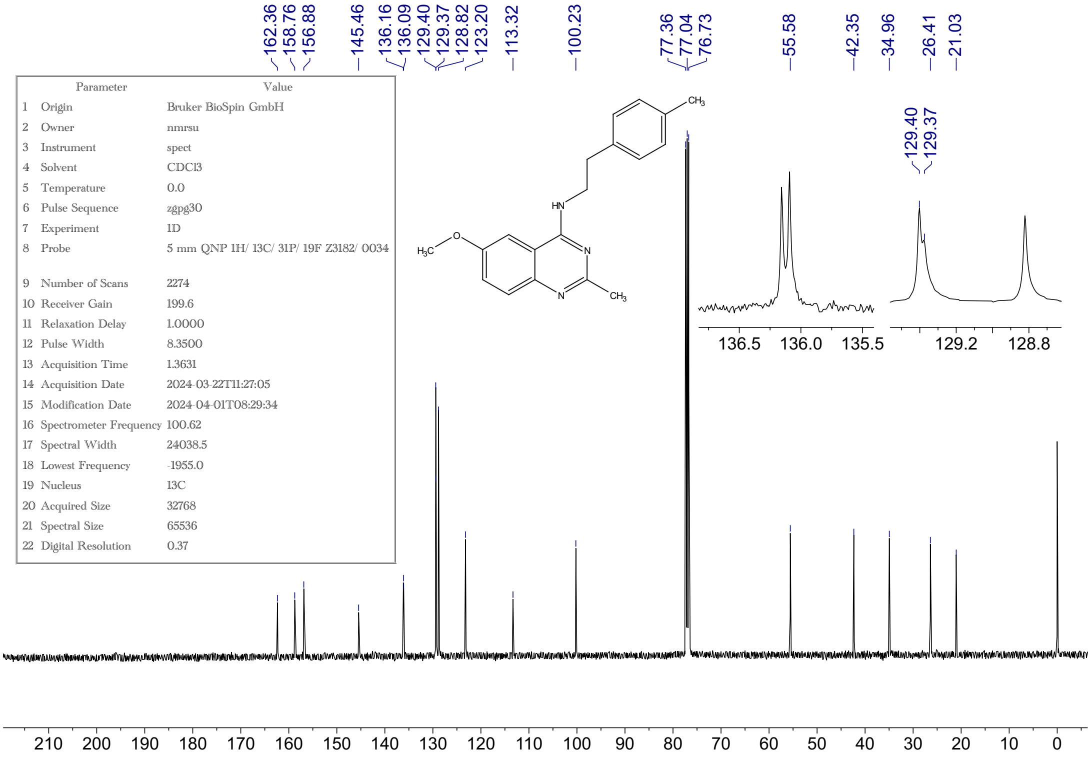

Figure S23. ^{13}C NMR spectrum (100 MHz) of compound **15b** in $CDCl_3$.

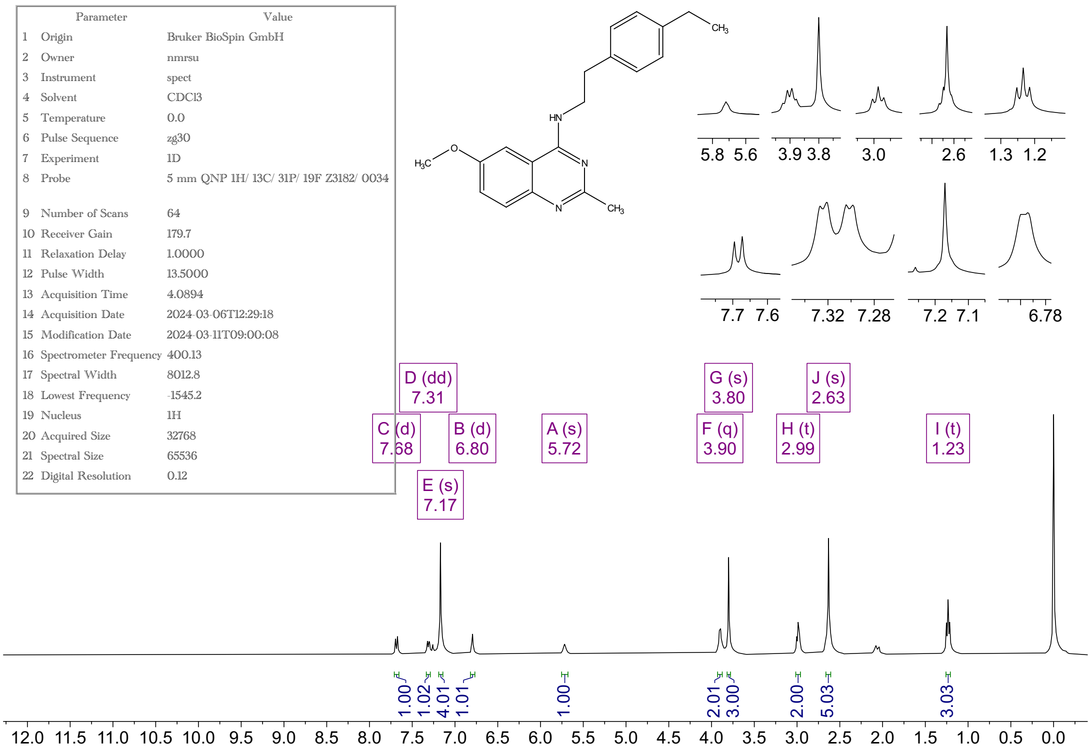

Figure S24. ^{1}H NMR spectrum (400 MHz) of compound **16b** in $CDCl_3$.

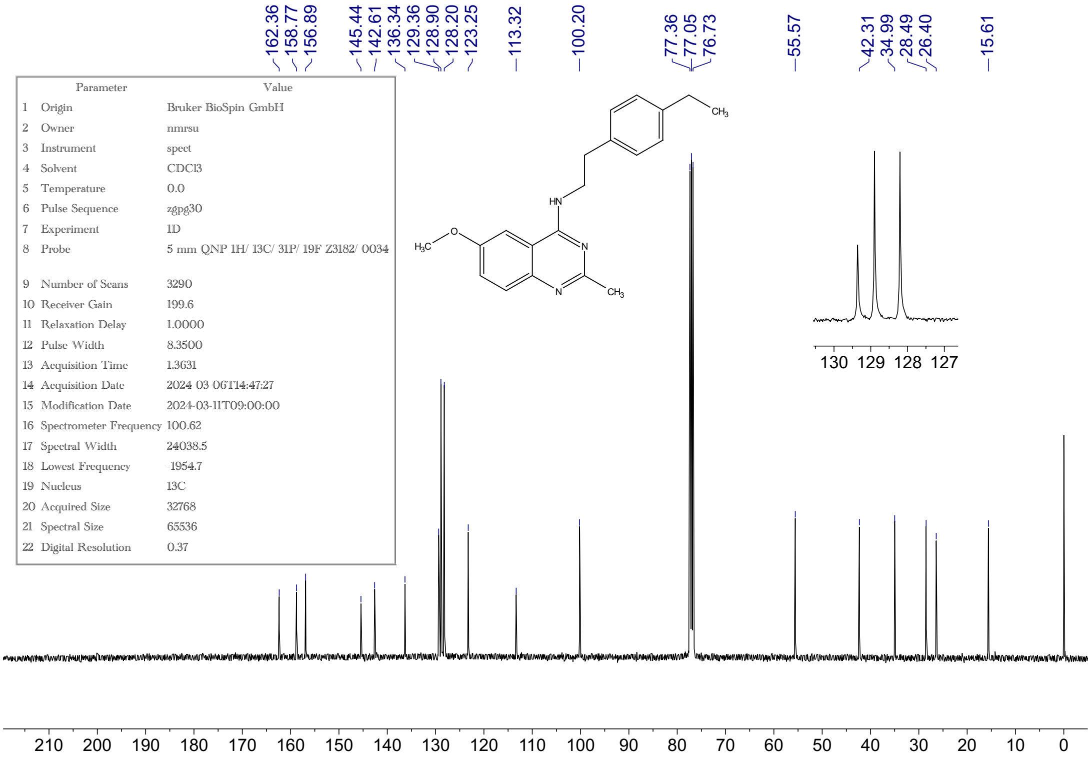

Figure S25. ^{13}C NMR spectrum (100 MHz) of compound **16b** in $CDCl_3$.

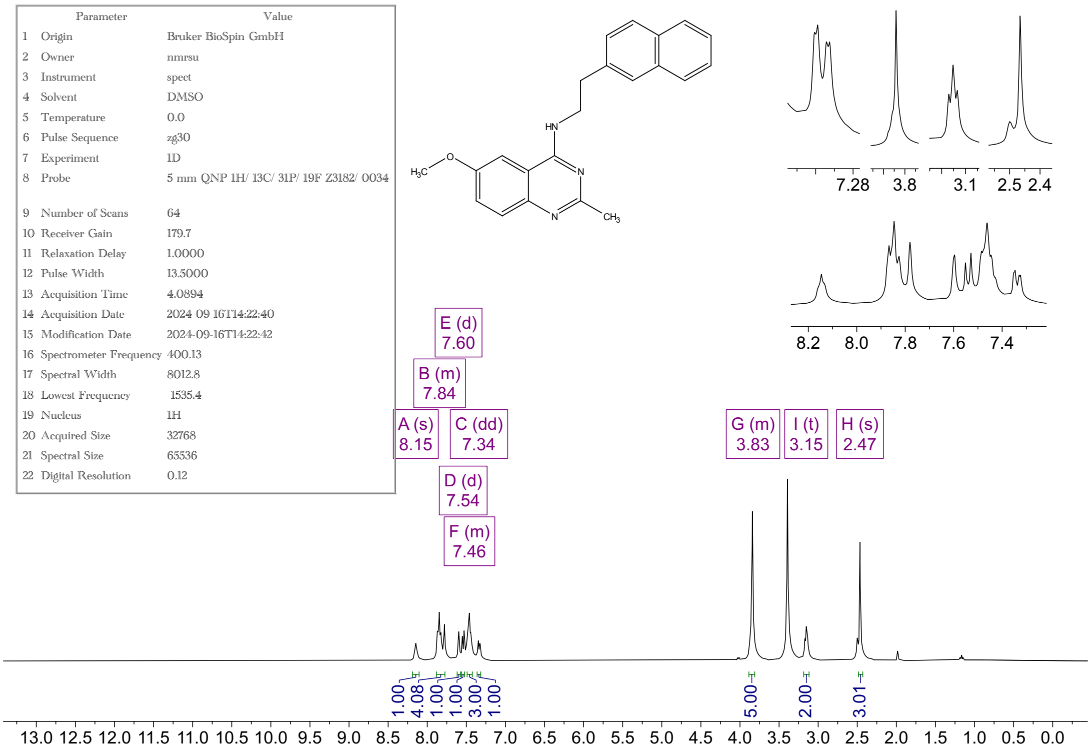

Figure S26. ^{1}H NMR spectrum (400 MHz) of compound **17b** in DMSO-d$_6$.

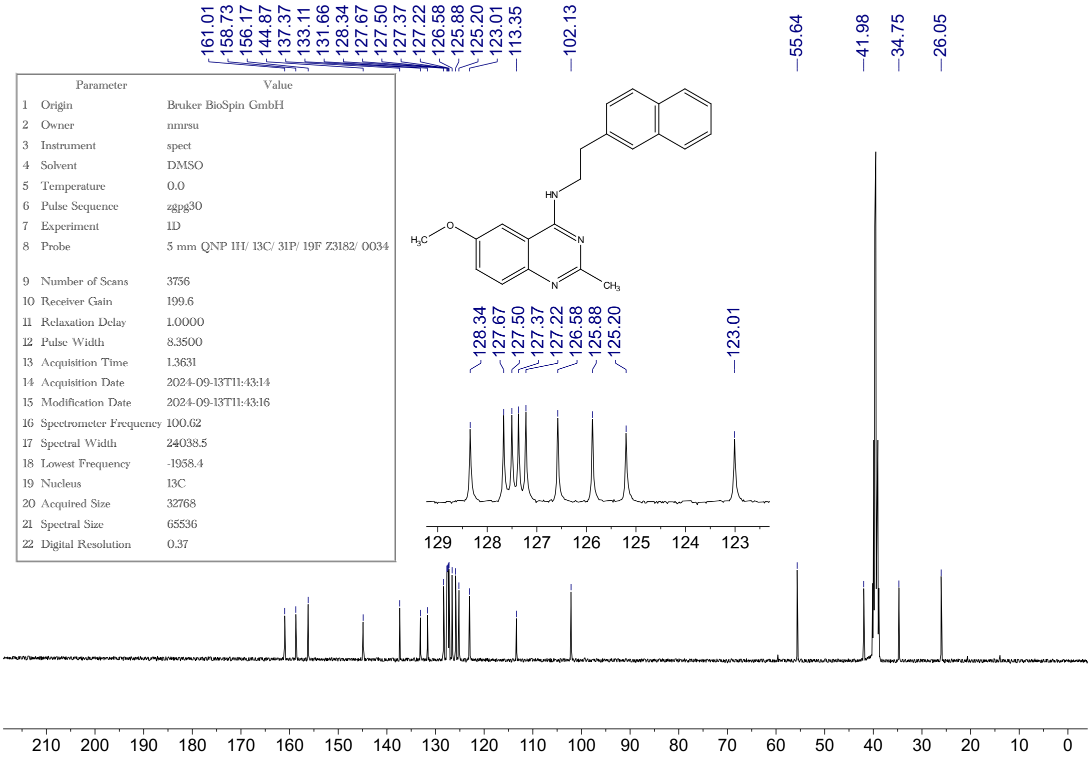

Figure S27. ^{13}C NMR spectrum (100 MHz) of compound **17b** in DMSO-d$_6$.

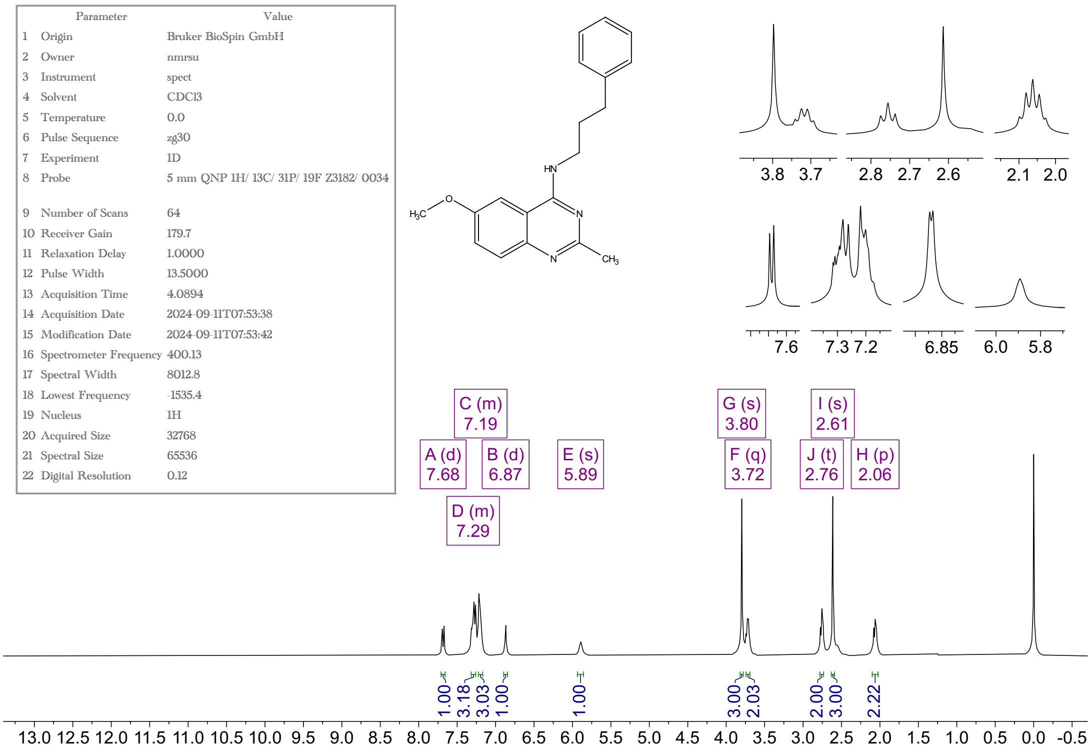

Figure S28. ^{1}H NMR spectrum (400 MHz) of compound **18b** in $CDCl_3$.

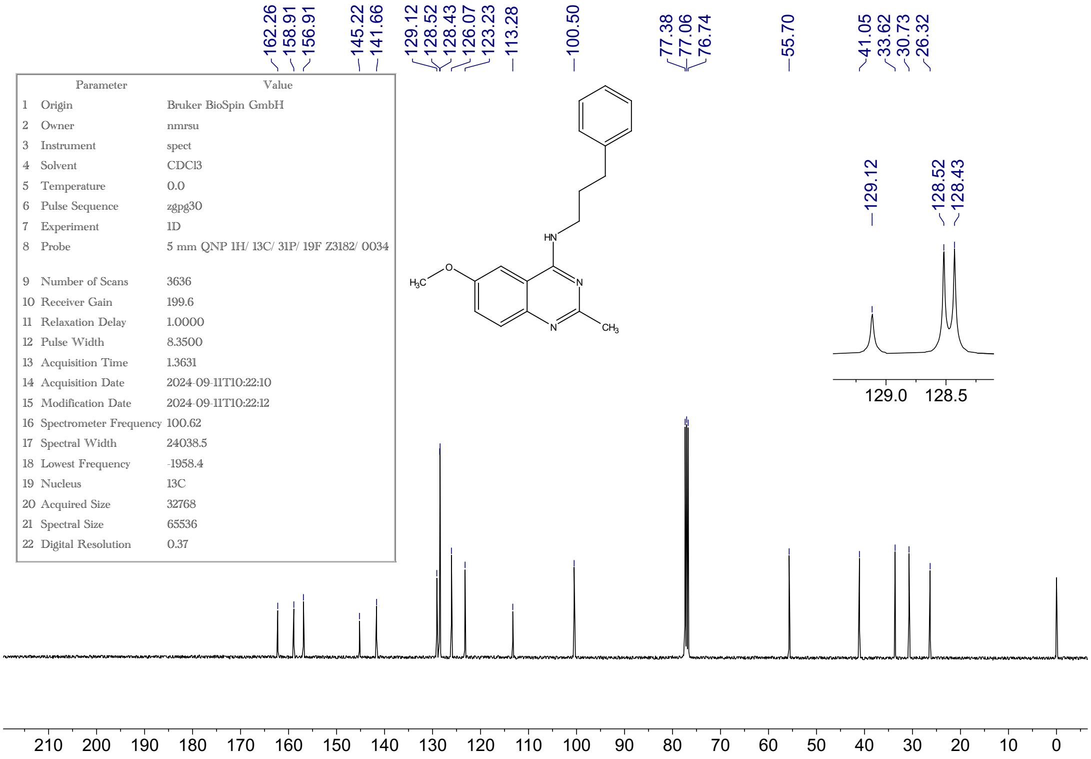

Figure S29. ^{13}C NMR spectrum (100 MHz) of compound **18b** in $CDCl_3$.

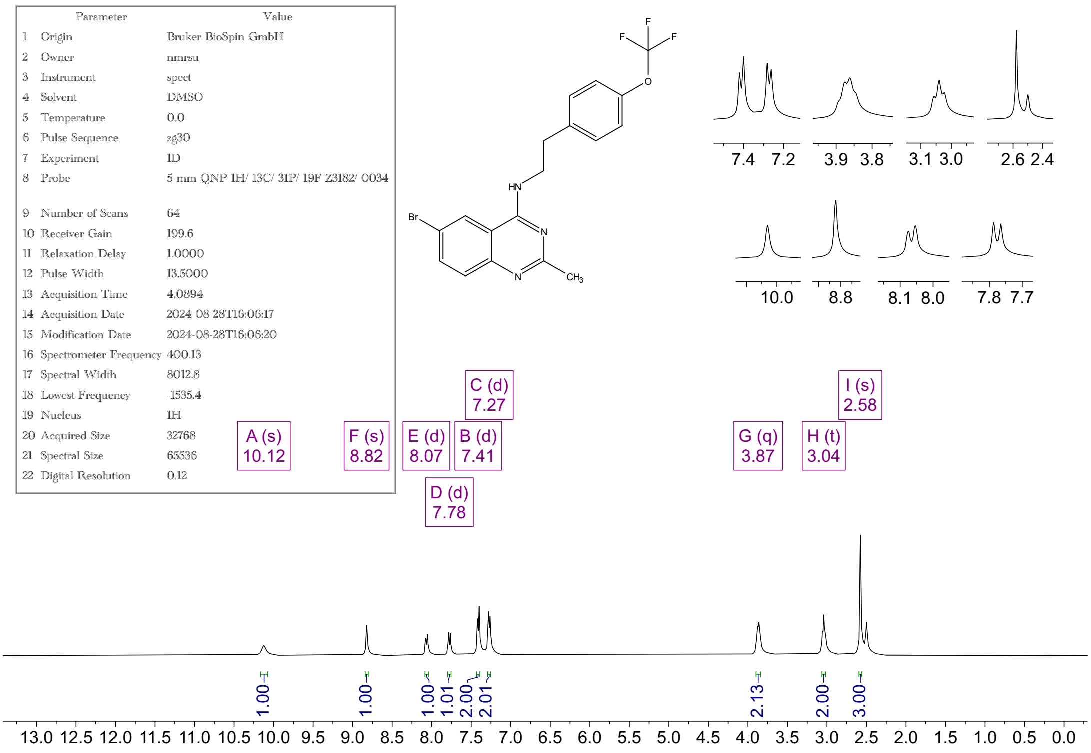

Figure S30. ^{1}H NMR spectrum (400 MHz) of compound **13c** in DMSO-d$_6$.

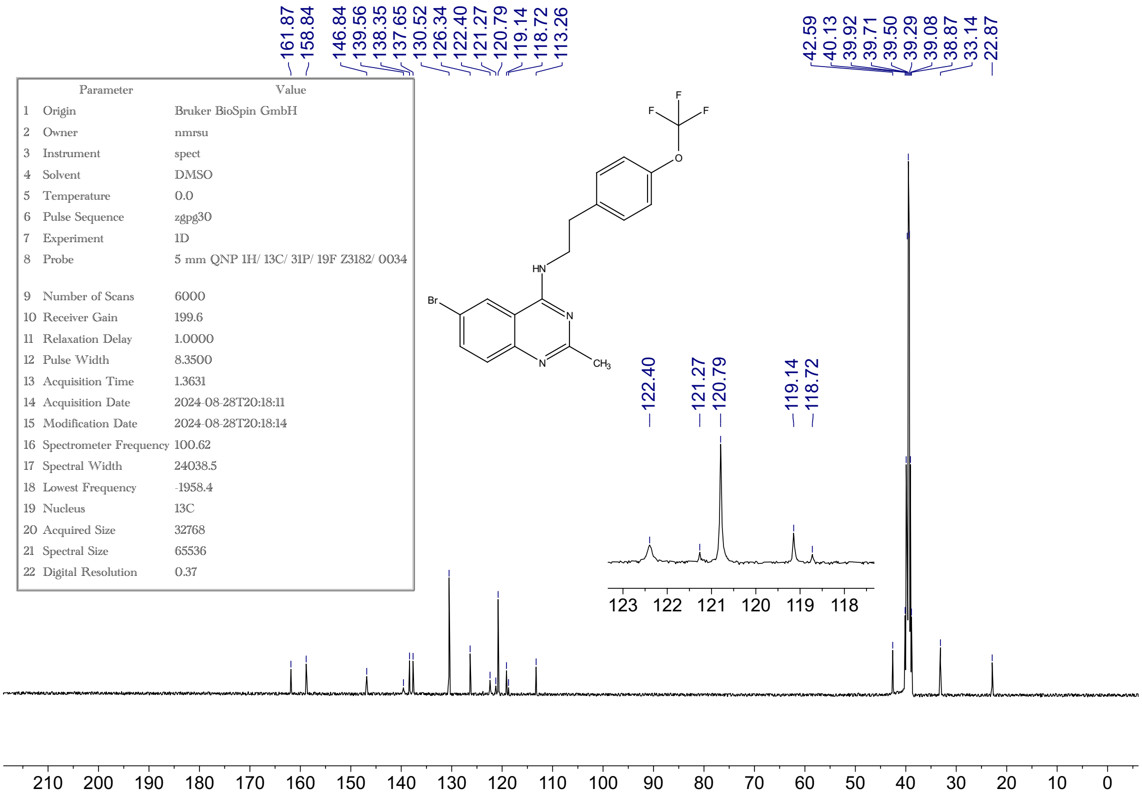

Figure S31. ^{13}C NMR spectrum (100 MHz) of compound **13c** in DMSO-d$_6$.

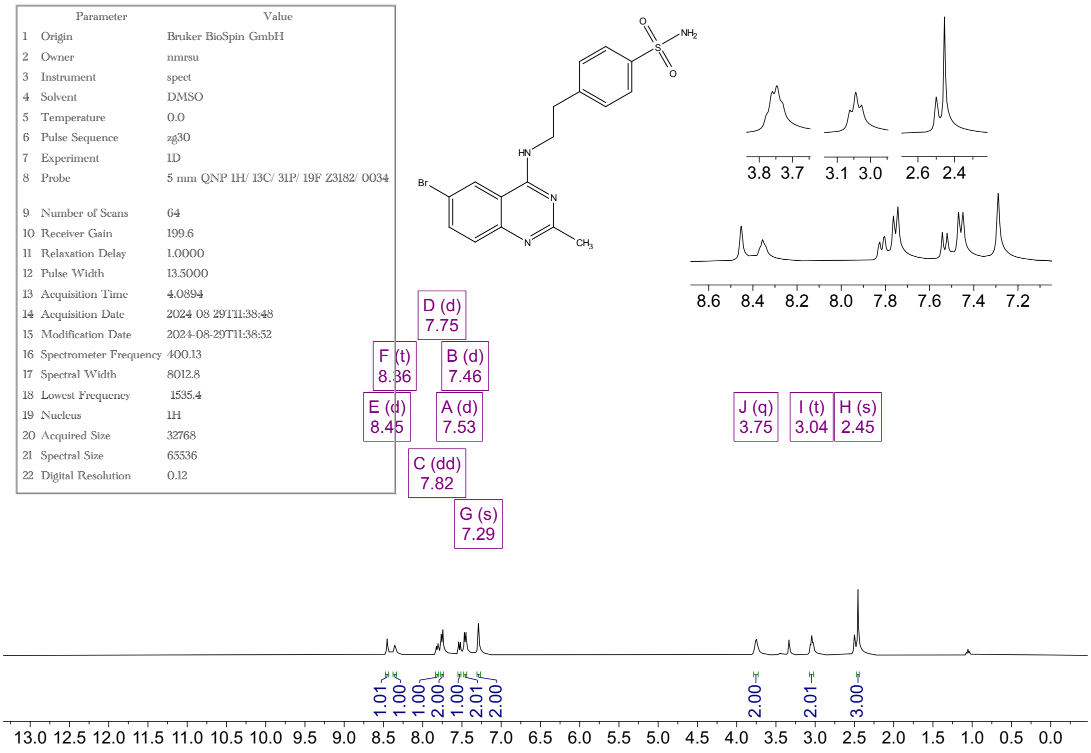

Figure S32. ^{1}H NMR spectrum (400 MHz) of compound **14c** in DMSO-d_6.

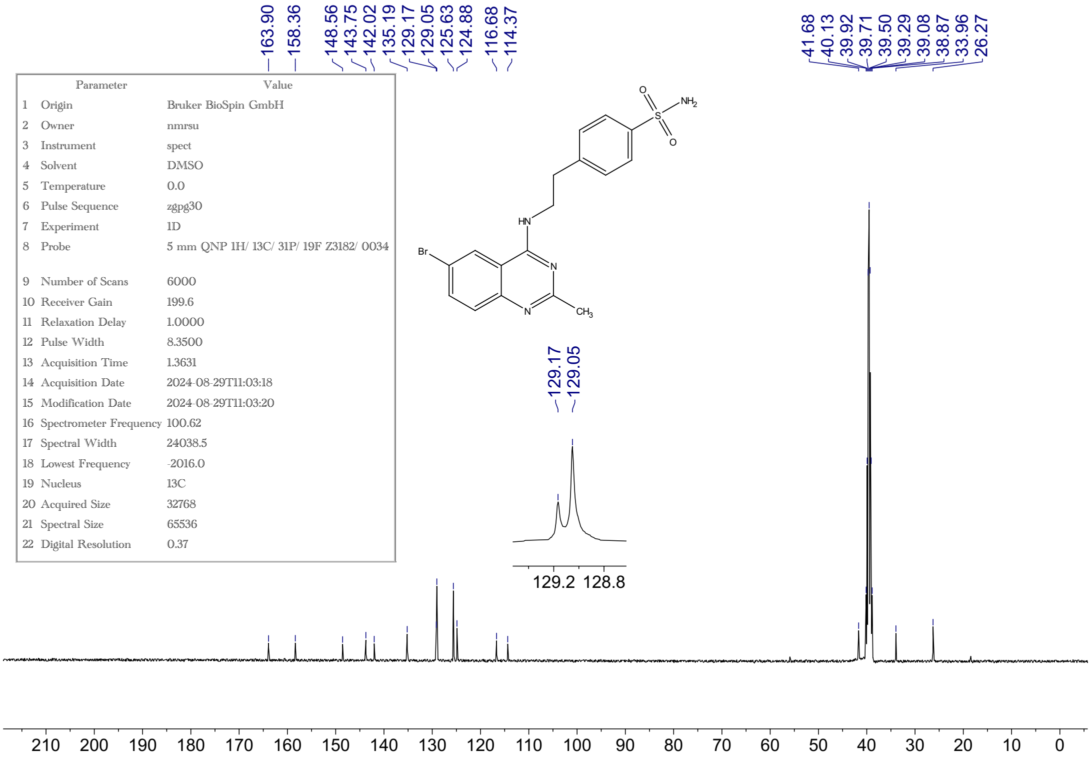

Figure S33. ^{13}C NMR spectrum (100 MHz) of compound **14c** in DMSO-d_6.

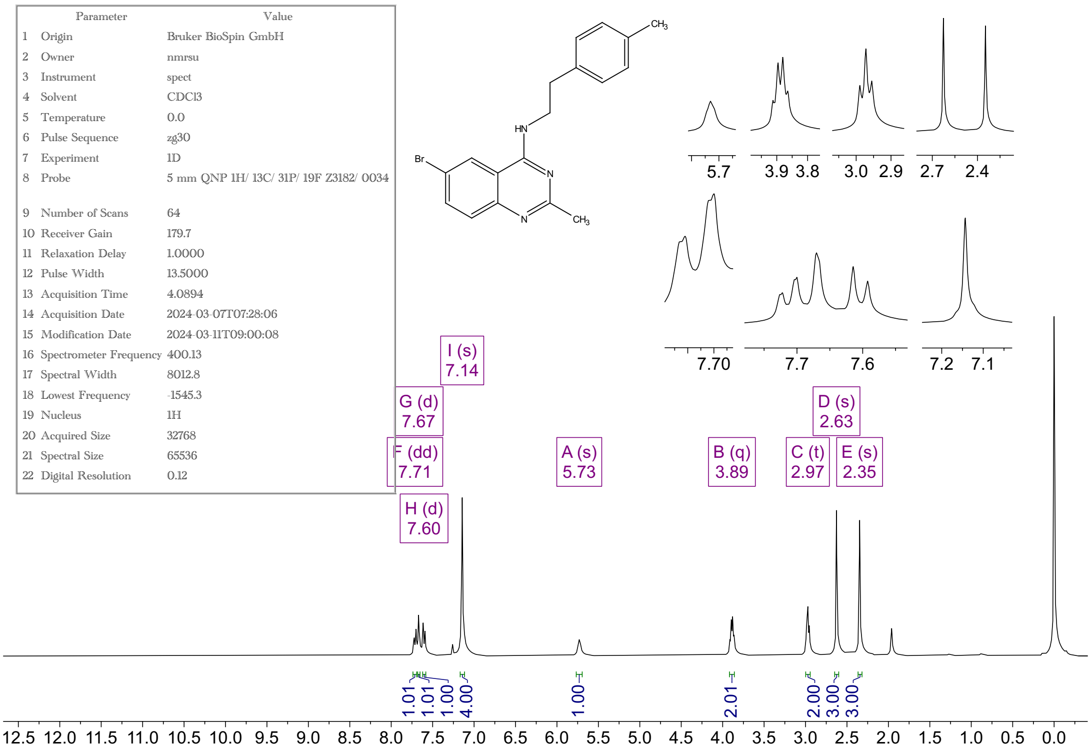

Figure S34. ^{1}H NMR spectrum (400 MHz) of compound **15c** in $CDCl_3$.

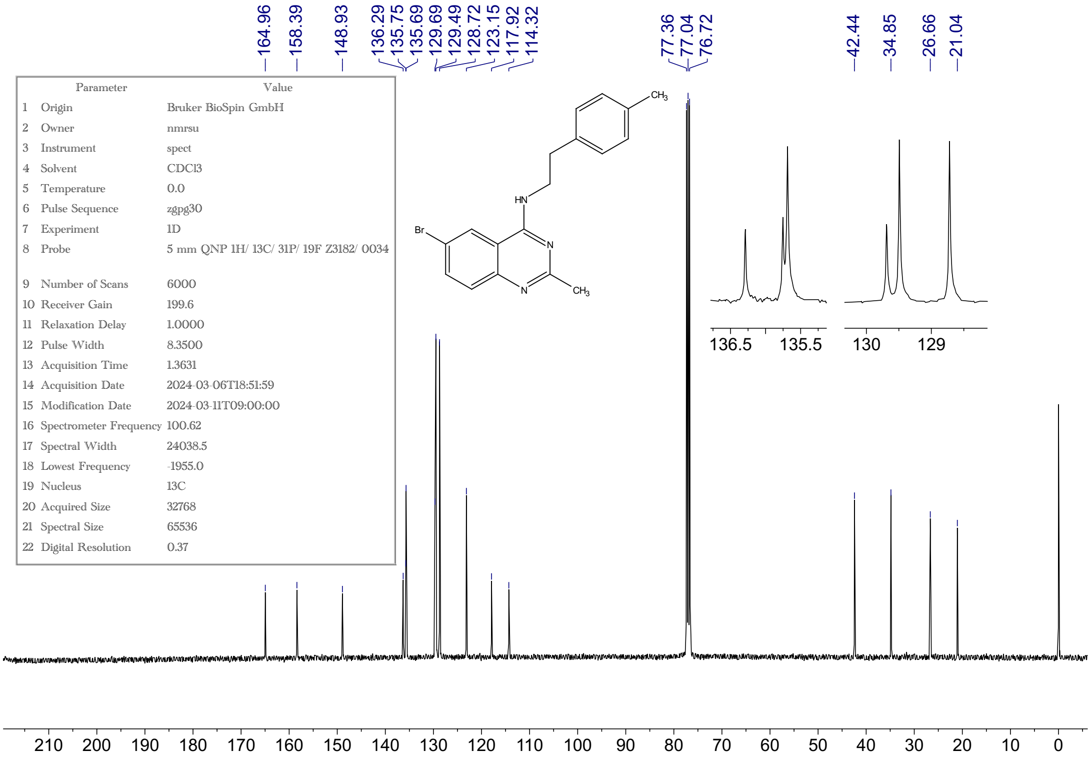

Figure S35. ^{13}C NMR spectrum (100 MHz) of compound **15c** in $CDCl_3$.

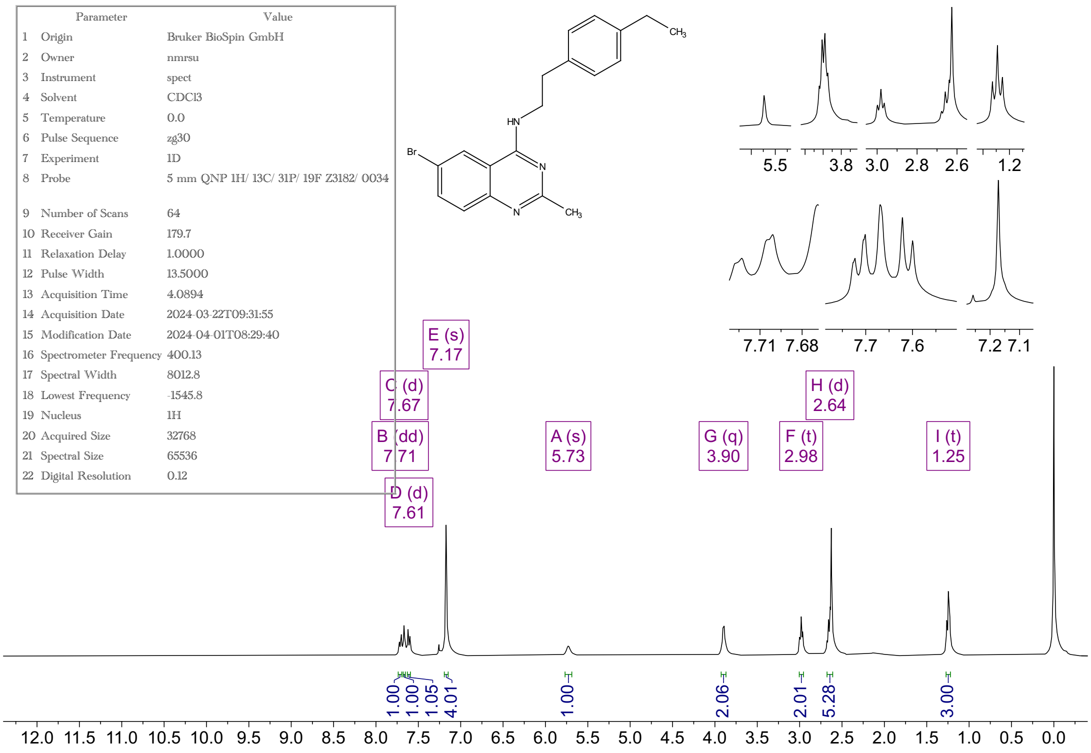

Figure S36. ^1H NMR spectrum (400 MHz) of compound **16c** in $CDCl_3$.

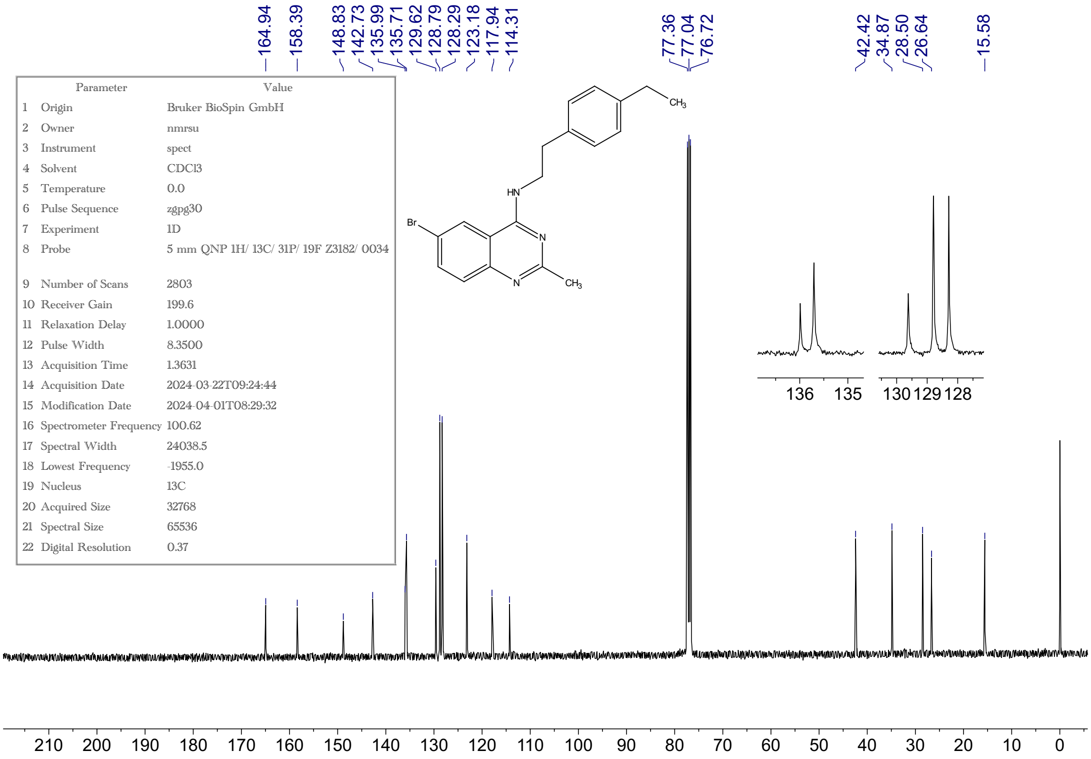

Figure S37. ^{13}C NMR spectrum (100 MHz) of compound **16c** in $CDCl_3$.

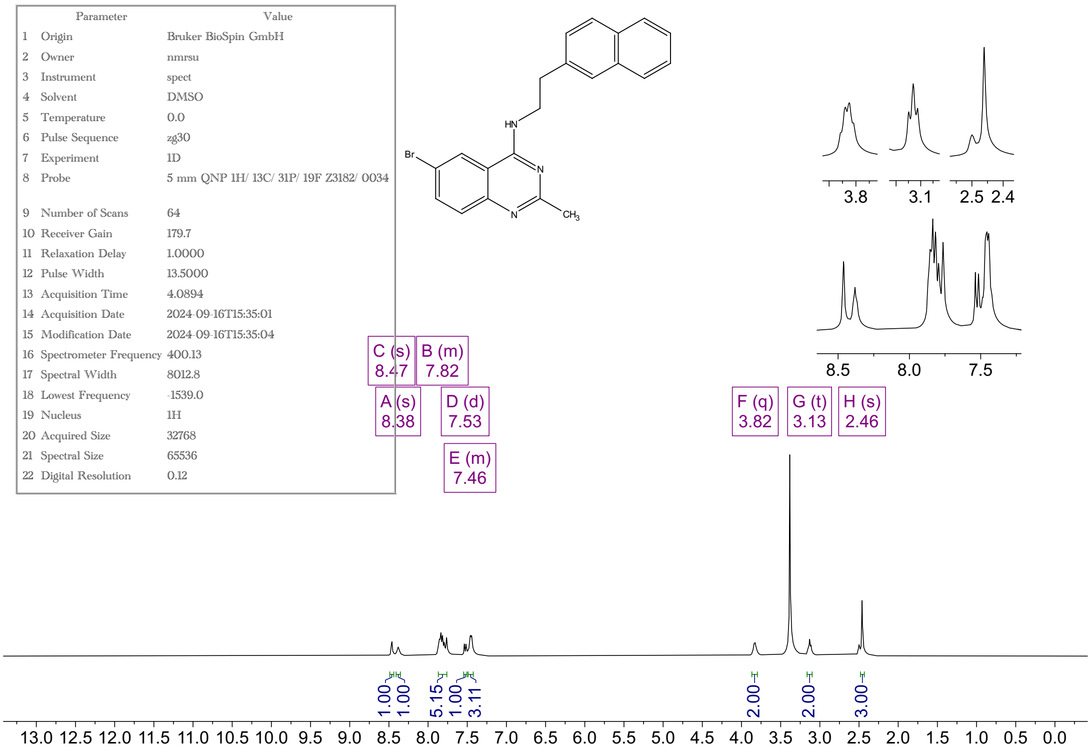

Figure S38. ^{1}H NMR spectrum (400 MHz) of compound **17c** in DMSO-d_6.

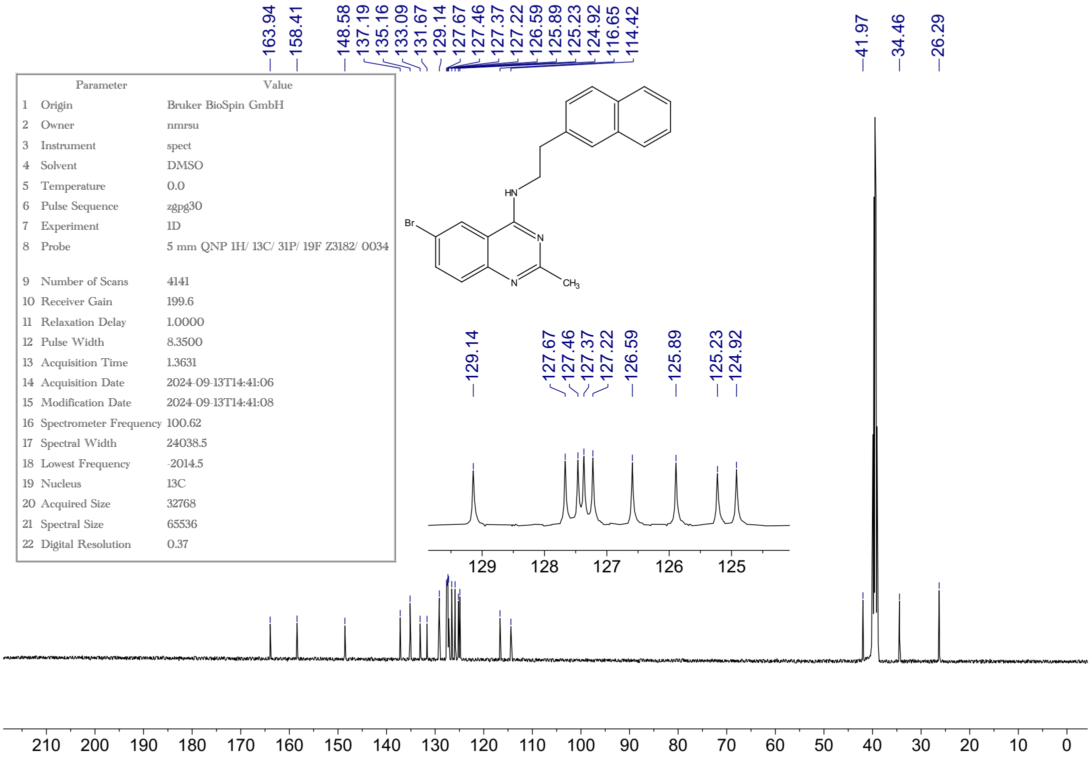

Figure S39. ^{13}C NMR spectrum (100 MHz) of compound **17c** in DMSO-d_6.

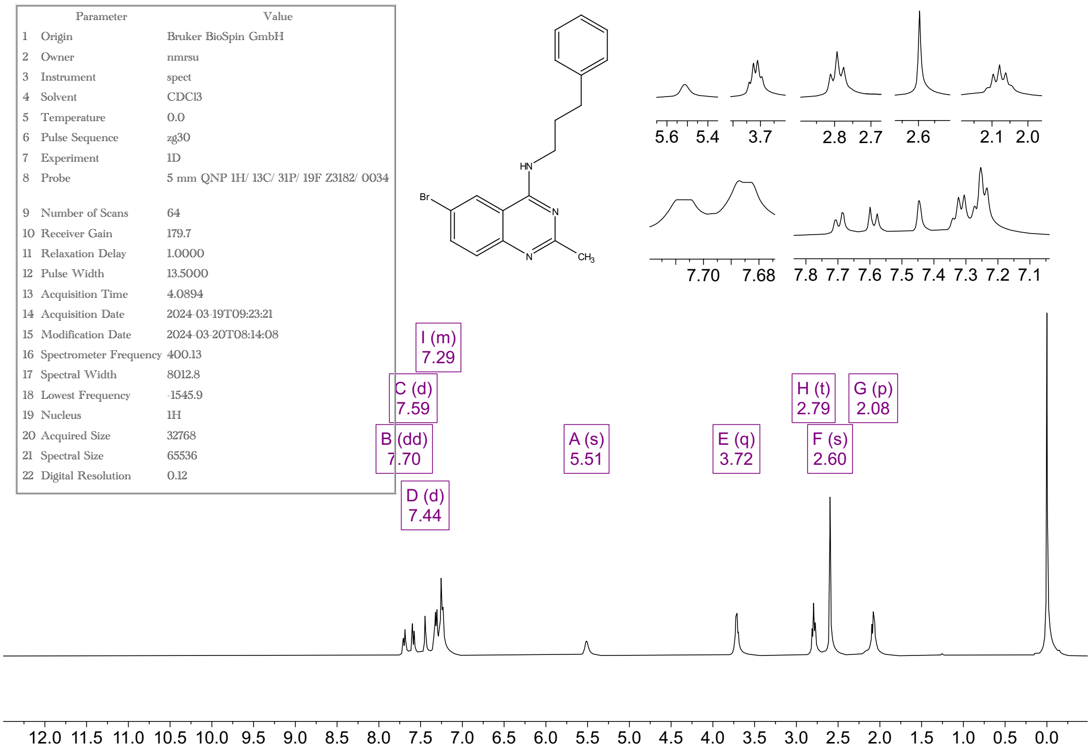

Figure S40. ^{1}H NMR spectrum (400 MHz) of compound **18c** in $CDCl_3$.

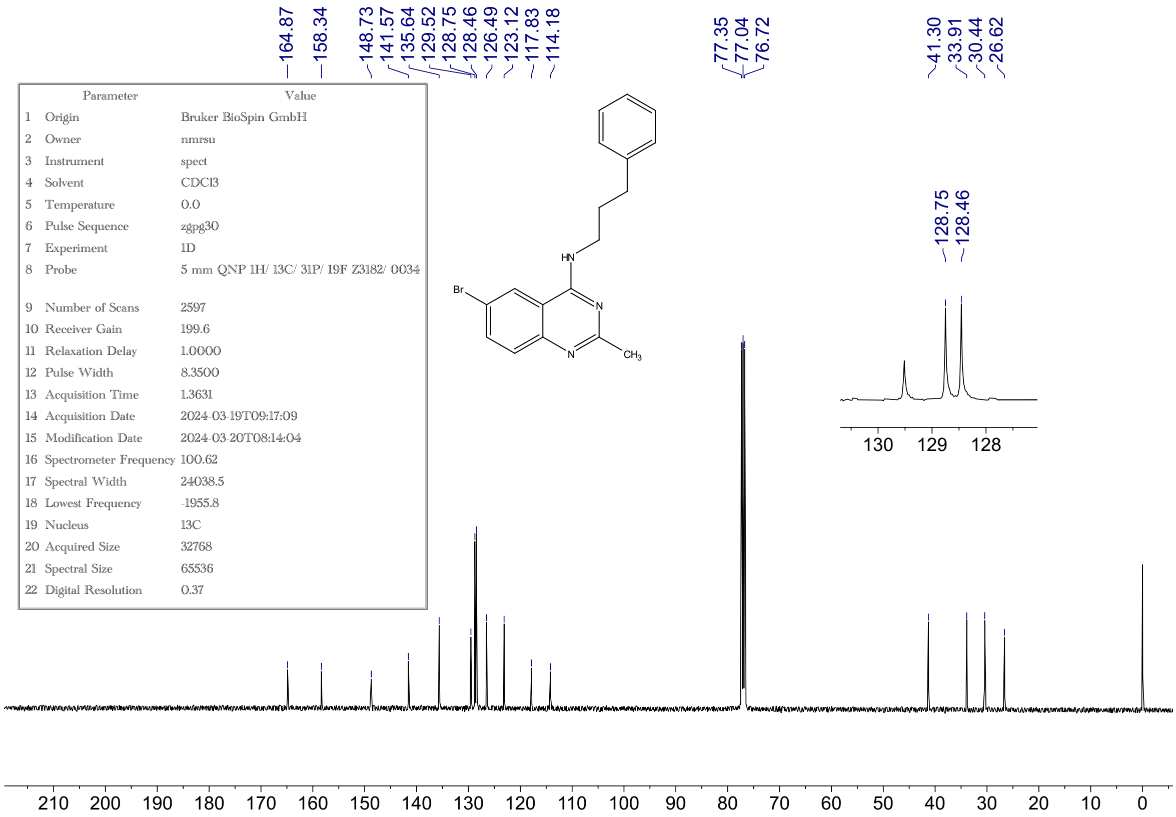

Figure S41. ^{13}C NMR spectrum (100 MHz) of compound **18c** in $CDCl_3$.

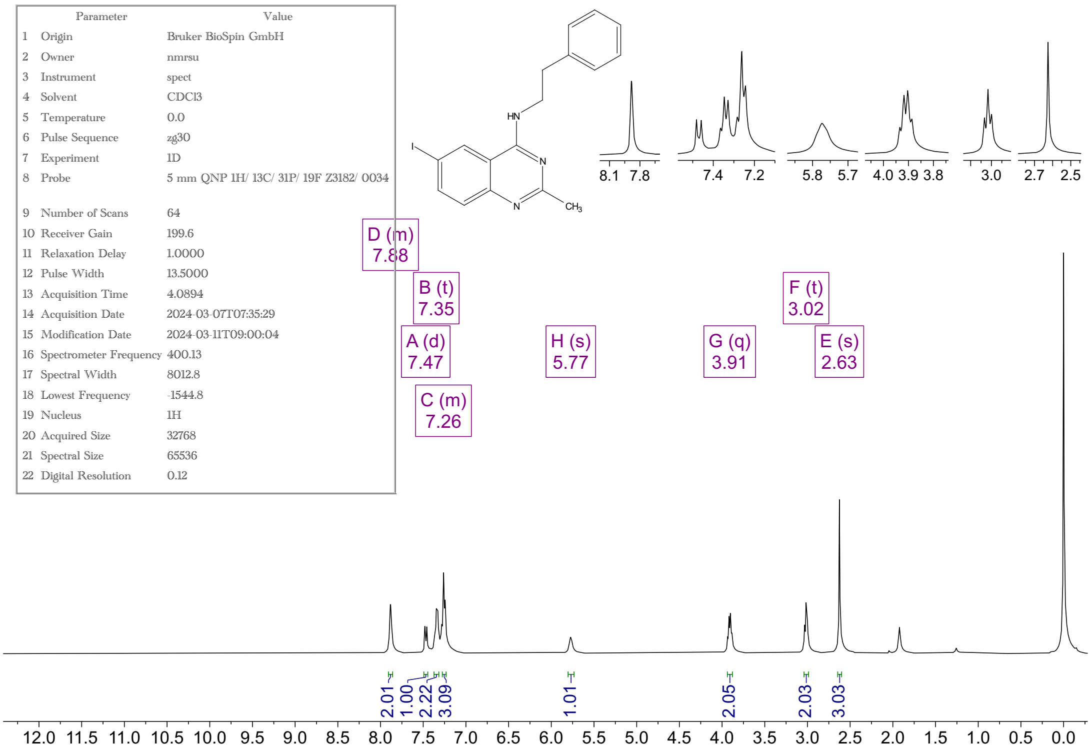

Figure S42. ^{1}H NMR spectrum (400 MHz) of compound **9d** in $CDCl_3$.

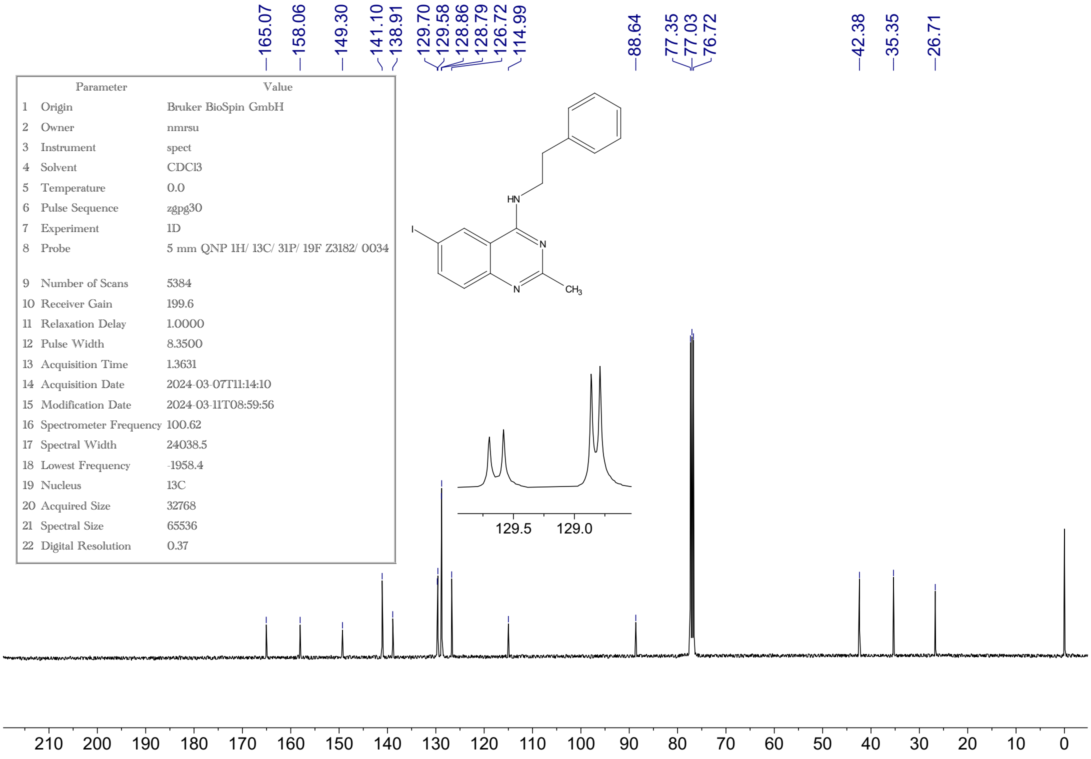

Figure S43. ^{13}C NMR spectrum (100 MHz) of compound **9d** in $CDCl_3$.

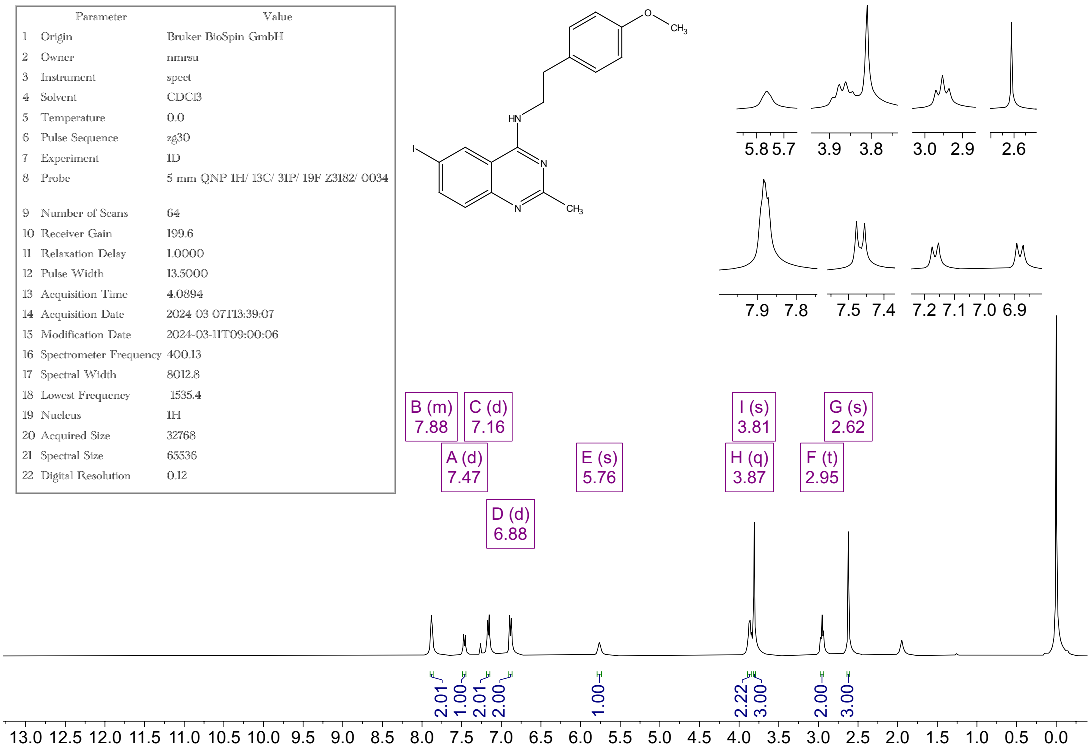

Figure S44. ^{1}H NMR spectrum (400 MHz) of compound **10d** in $CDCl_3$.

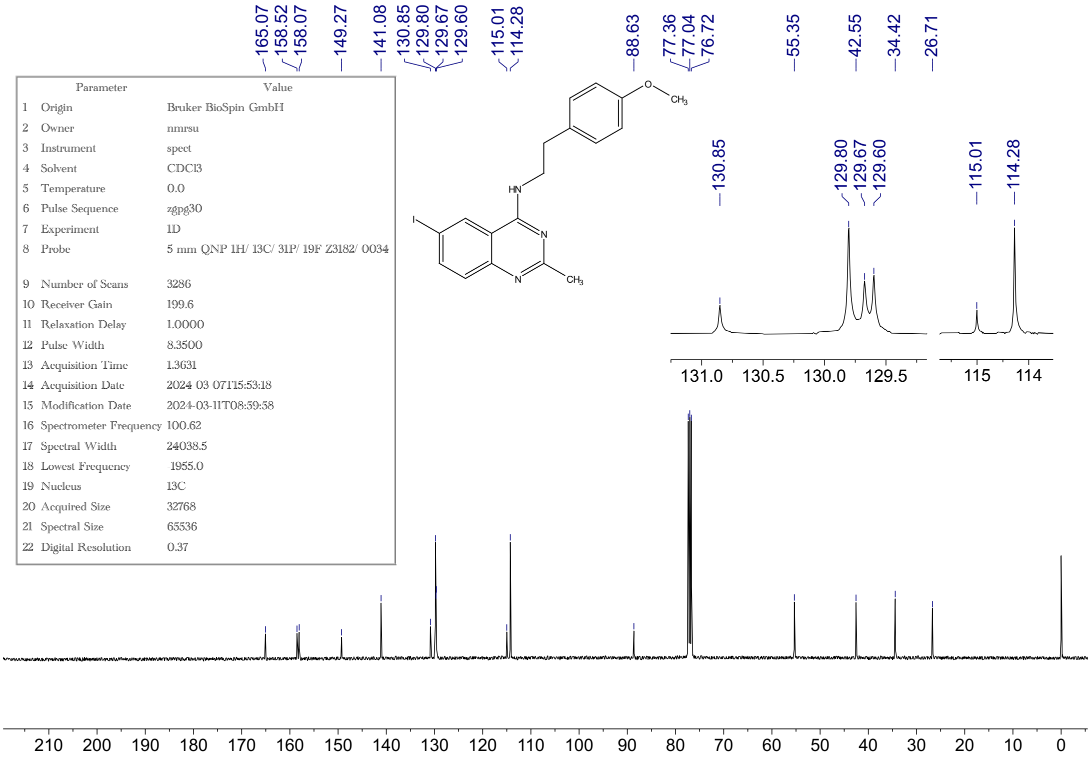

Figure S45. ^{13}C NMR spectrum (100 MHz) of compound **10d** in $CDCl_3$.

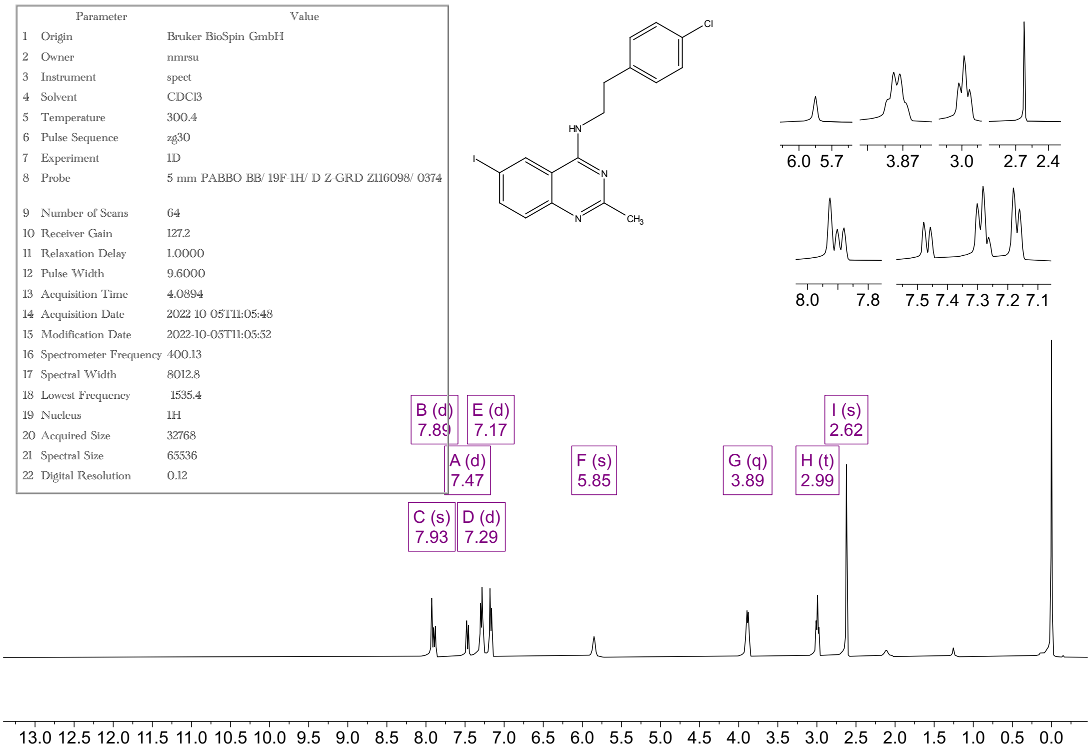

Figure S46. ^{1}H NMR spectrum (400 MHz) of compound **11d** in $CDCl_3$.

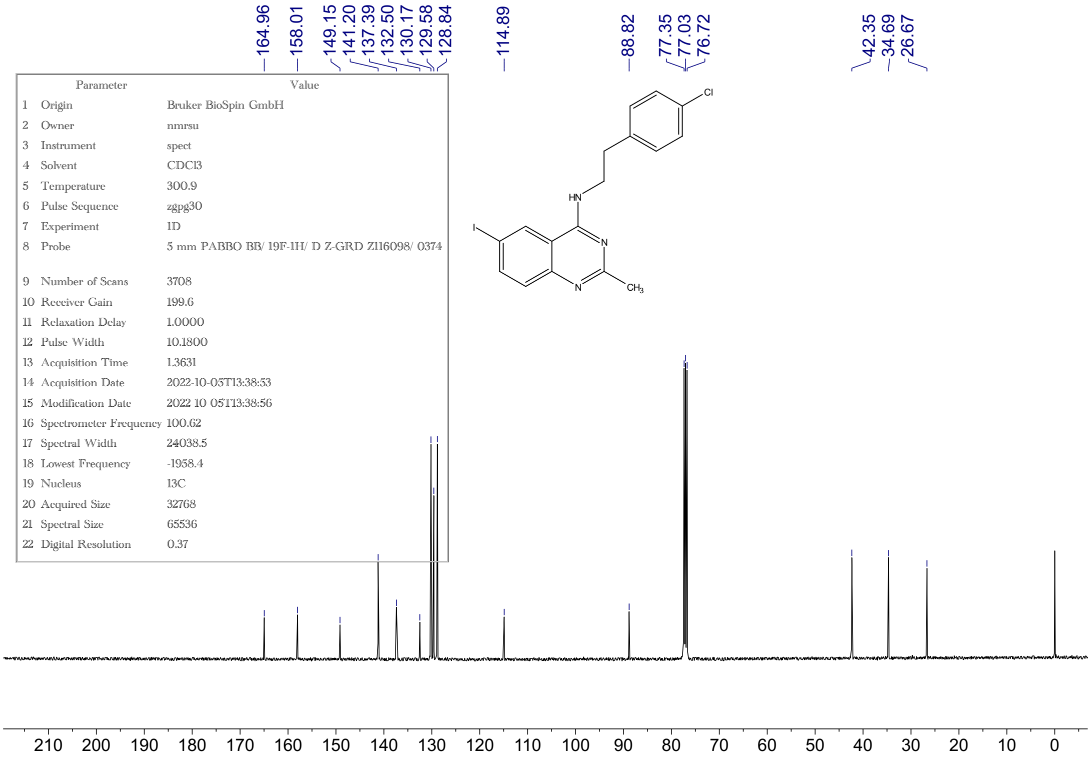

Figure S47. ^{13}C NMR spectrum (100 MHz) of compound **11d** in $CDCl_3$.

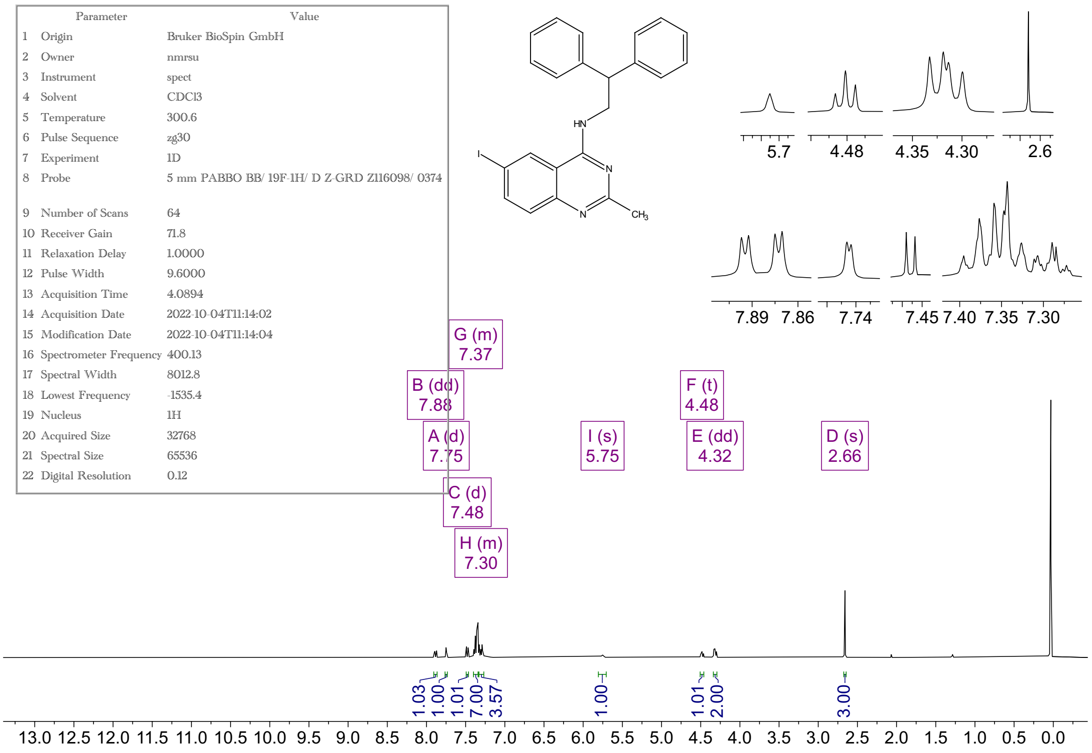

Figure S48. ^{1}H NMR spectrum (400 MHz) of compound **12d** in $CDCl_3$.

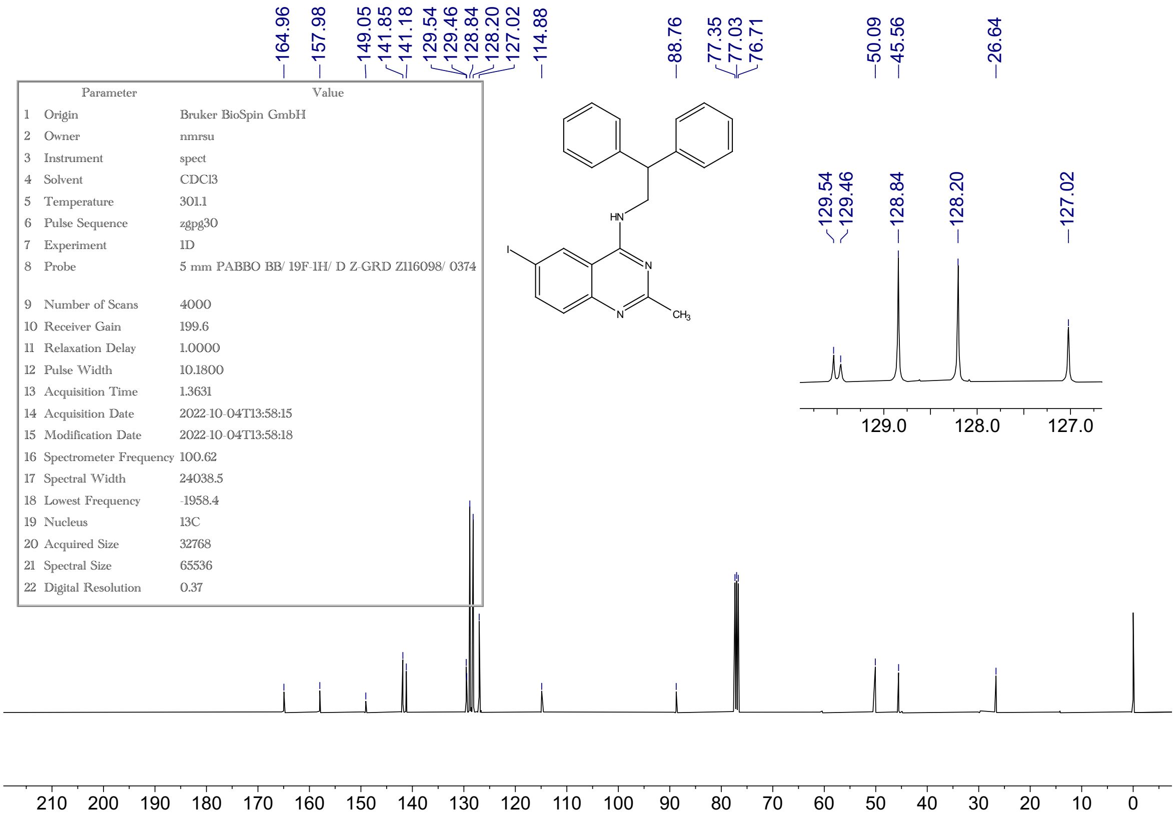

Figure S49. ^{13}C NMR spectrum (100 MHz) of compound **12d** in $CDCl_3$.

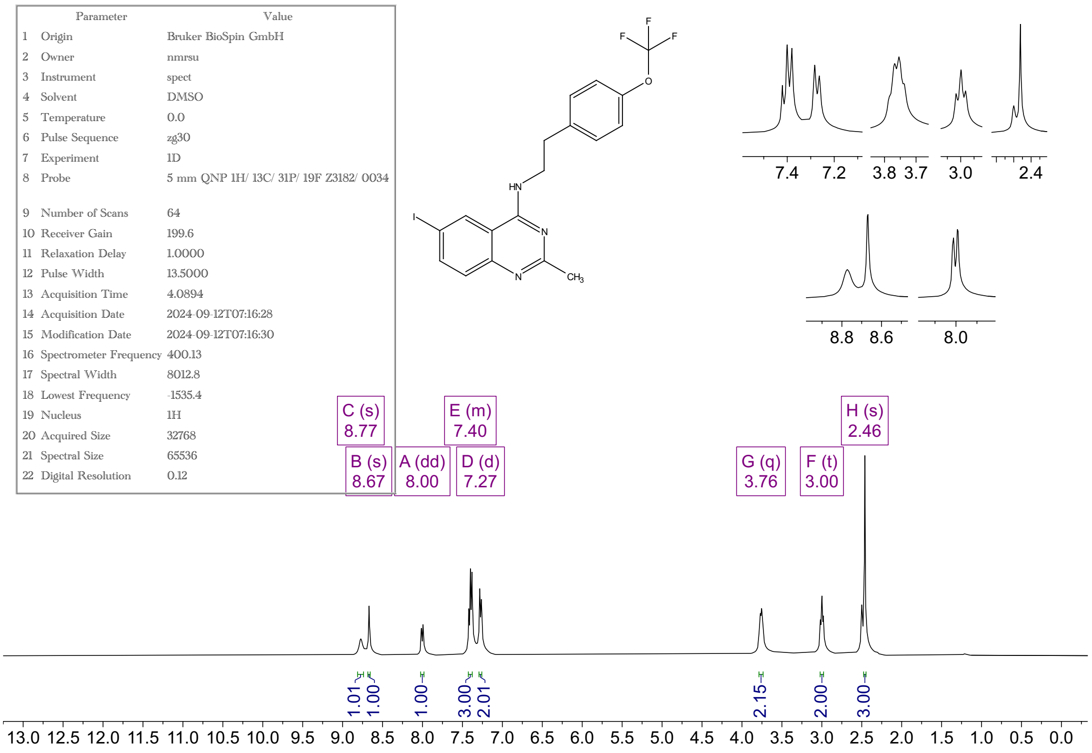

Figure S50. ^{1}H NMR spectrum (400 MHz) of compound **13d** in DMSO-d$_6$.

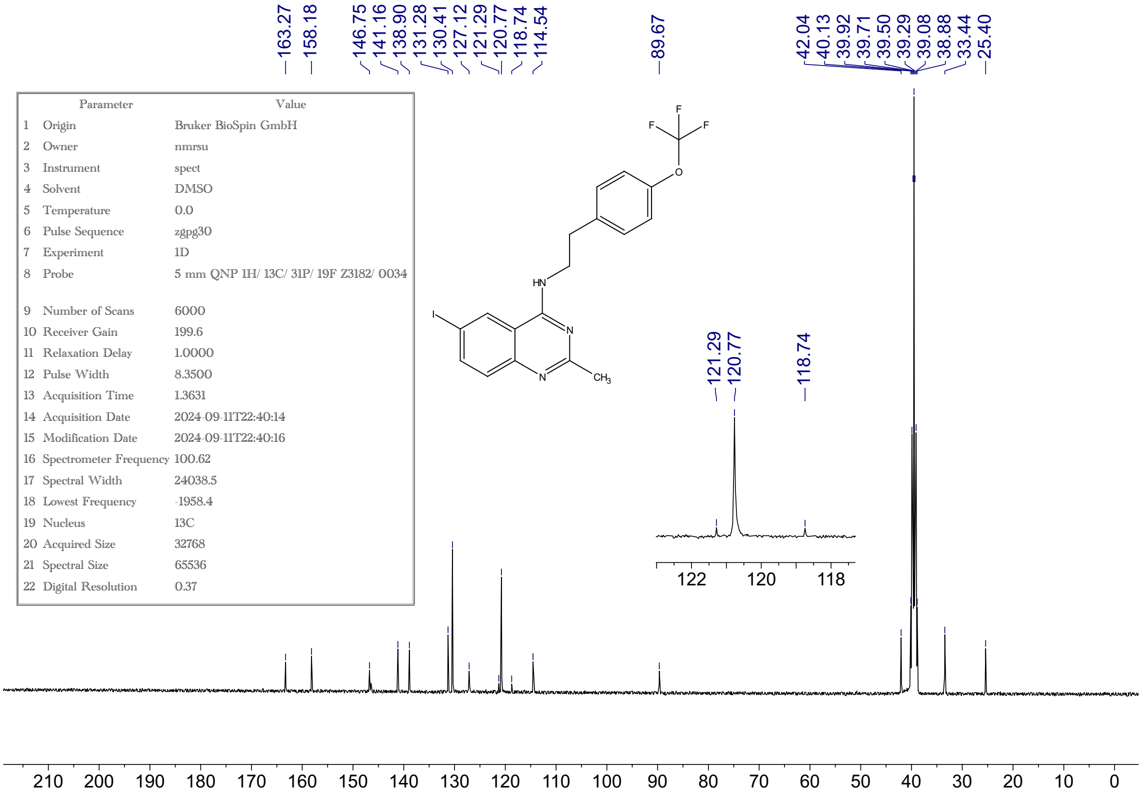

Figure S51. ^{13}C NMR spectrum (100 MHz) of compound **13d** in DMSO-d$_6$.

Parameter	Value
1 Origin	Bruker BioSpin GmbH
2 Owner	nmrsu
3 Instrument	spect
4 Solvent	DMSO
5 Temperature	0.0
6 Pulse Sequence	zg30
7 Experiment	1D
8 Probe	5 mm QNP 1H/ 13C/ 31P/ 19F Z3182/ 0034
9 Number of Scans	64
10 Receiver Gain	199.6
11 Relaxation Delay	1.0000
12 Pulse Width	13.5000
13 Acquisition Time	4.0894
14 Acquisition Date	2024-08-29T11:48:28
15 Modification Date	2024-08-29T11:48:30
16 Spectrometer Frequency	400.13
17 Spectral Width	8012.8
18 Lowest Frequency	-1538.6
19 Nucleus	1H
20 Acquired Size	32768
21 Spectral Size	65536
22 Digital Resolution	0.12

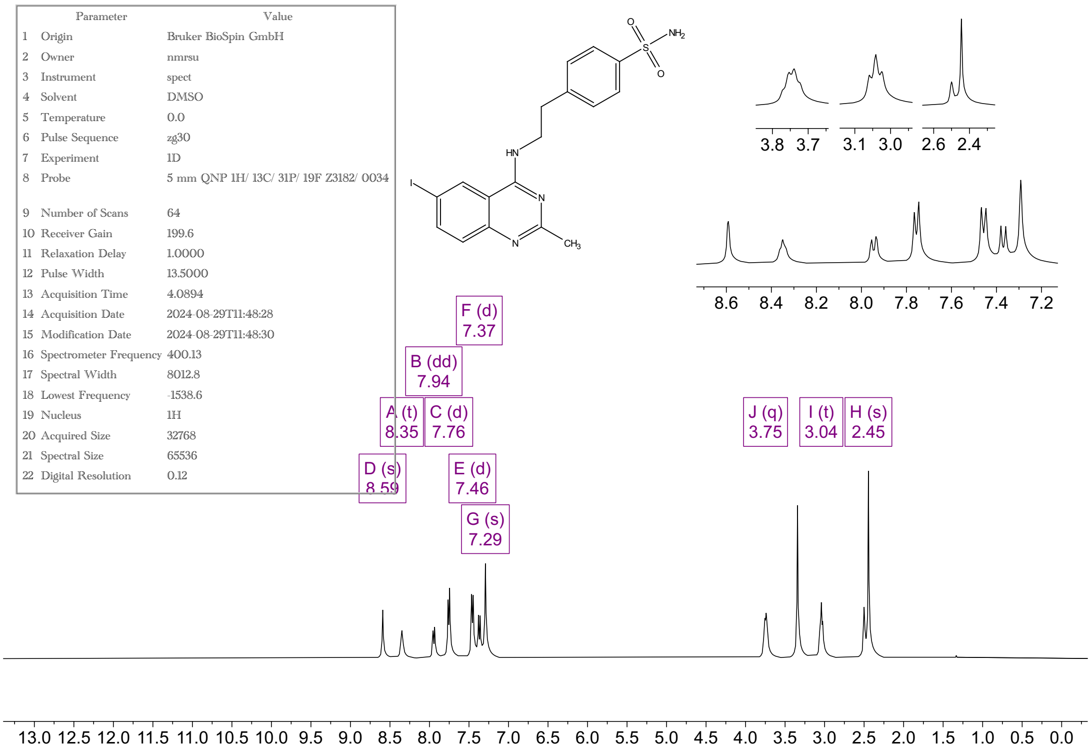

Figure S52. ^{1}H NMR spectrum (400 MHz) of compound **14d** in DMSO-d$_6$.

Parameter	Value
1 Origin	Bruker BioSpin GmbH
2 Owner	nmrsu
3 Instrument	spect
4 Solvent	DMSO
5 Temperature	0.0
6 Pulse Sequence	zgpg30
7 Experiment	1D
8 Probe	5 mm QNP 1H/ 13C/ 31P/ 19F Z3182/ 0034
9 Number of Scans	6000
10 Receiver Gain	199.6
11 Relaxation Delay	1.0000
12 Pulse Width	8.3500
13 Acquisition Time	1.3631
14 Acquisition Date	2024-08-29T15:52:08
15 Modification Date	2024-08-29T15:52:12
16 Spectrometer Frequency	100.62
17 Spectral Width	24038.5
18 Lowest Frequency	-2016.0
19 Nucleus	13C
20 Acquired Size	32768
21 Spectral Size	65536
22 Digital Resolution	0.37

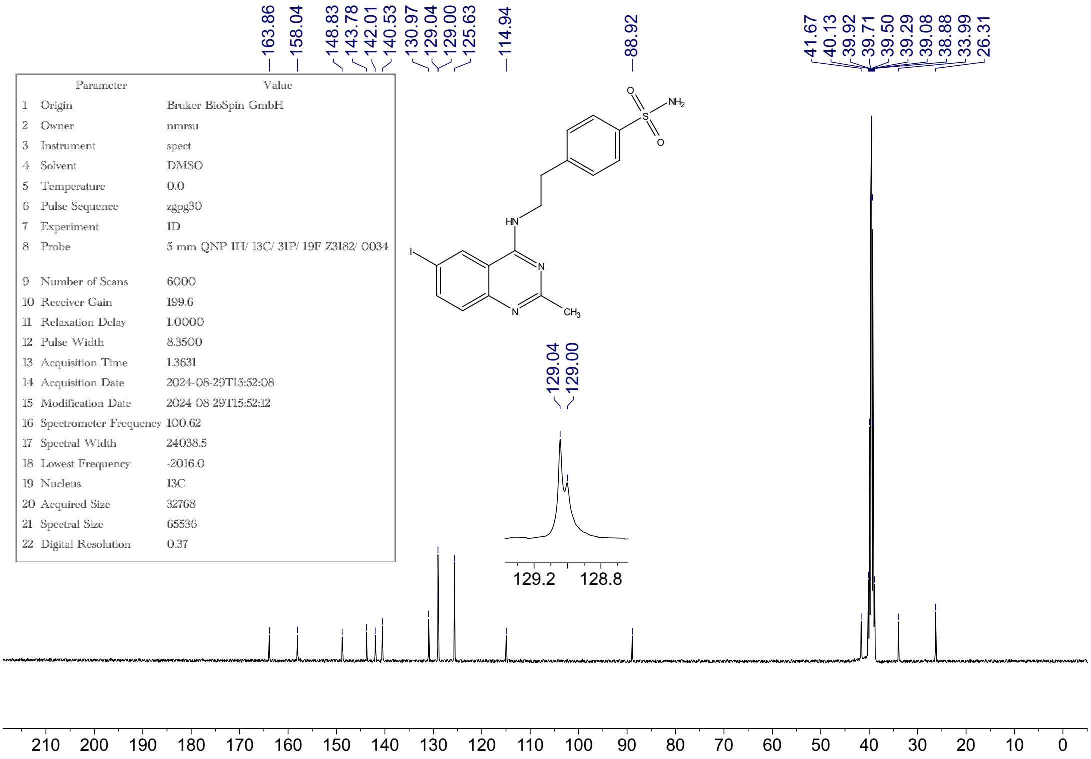

Figure S53. ^{13}C NMR spectrum (100 MHz) of compound **14d** in DMSO-d$_6$.

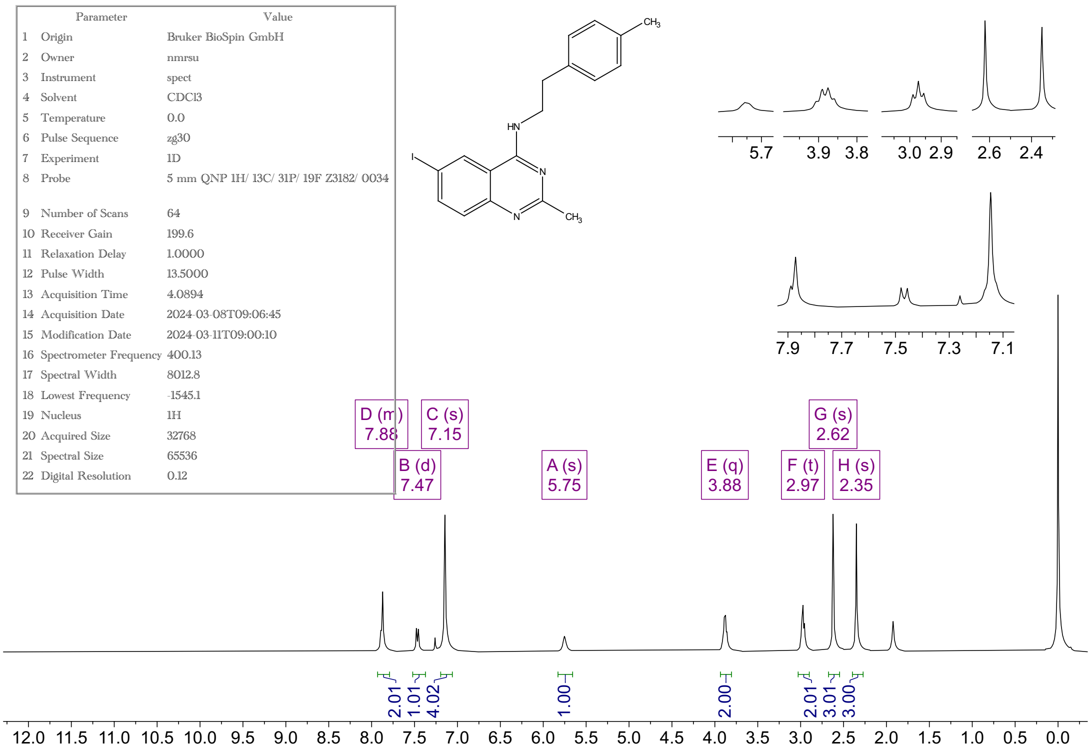

Figure S54. ^{1}H NMR spectrum (400 MHz) of compound **15d** in $CDCl_3$.

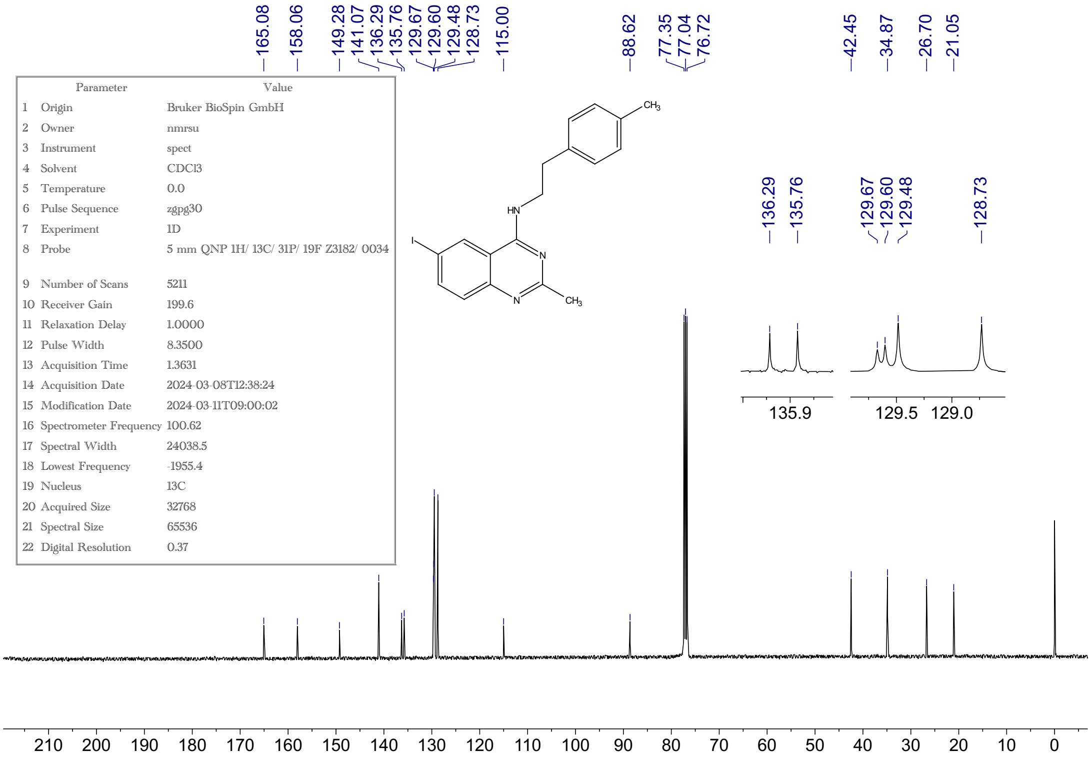

Figure S55. ^{13}C NMR spectrum (100 MHz) of compound **15d** in $CDCl_3$.

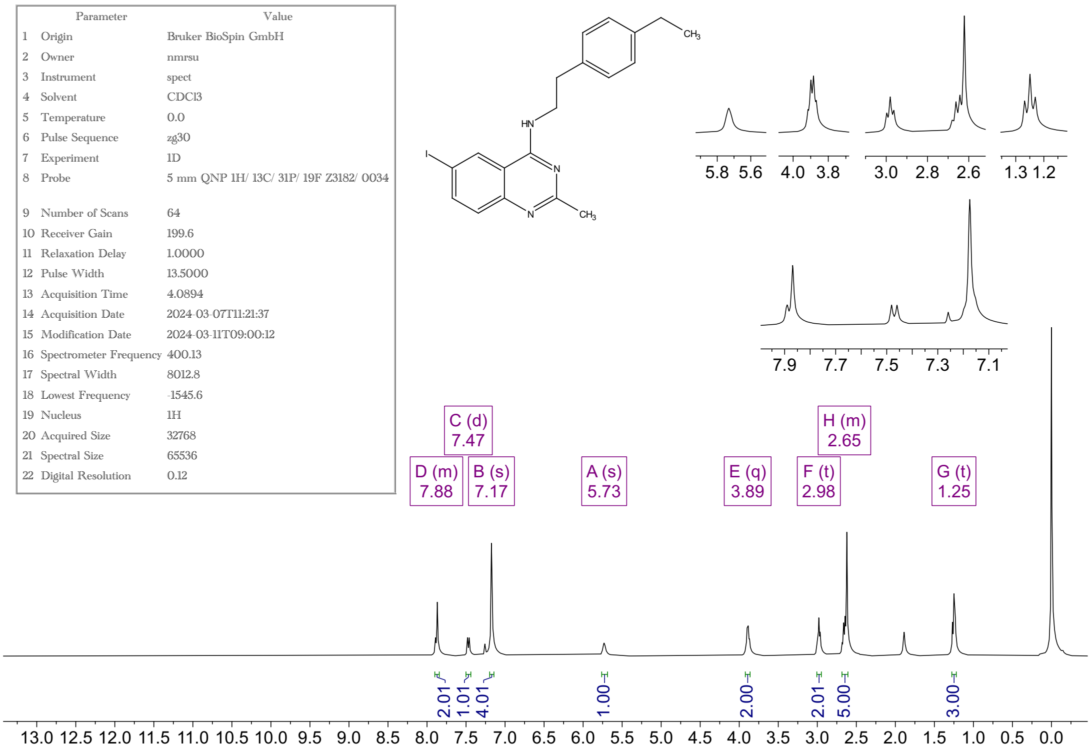

Figure S56. ^{1}H NMR spectrum (400 MHz) of compound **16d** in $CDCl_3$.

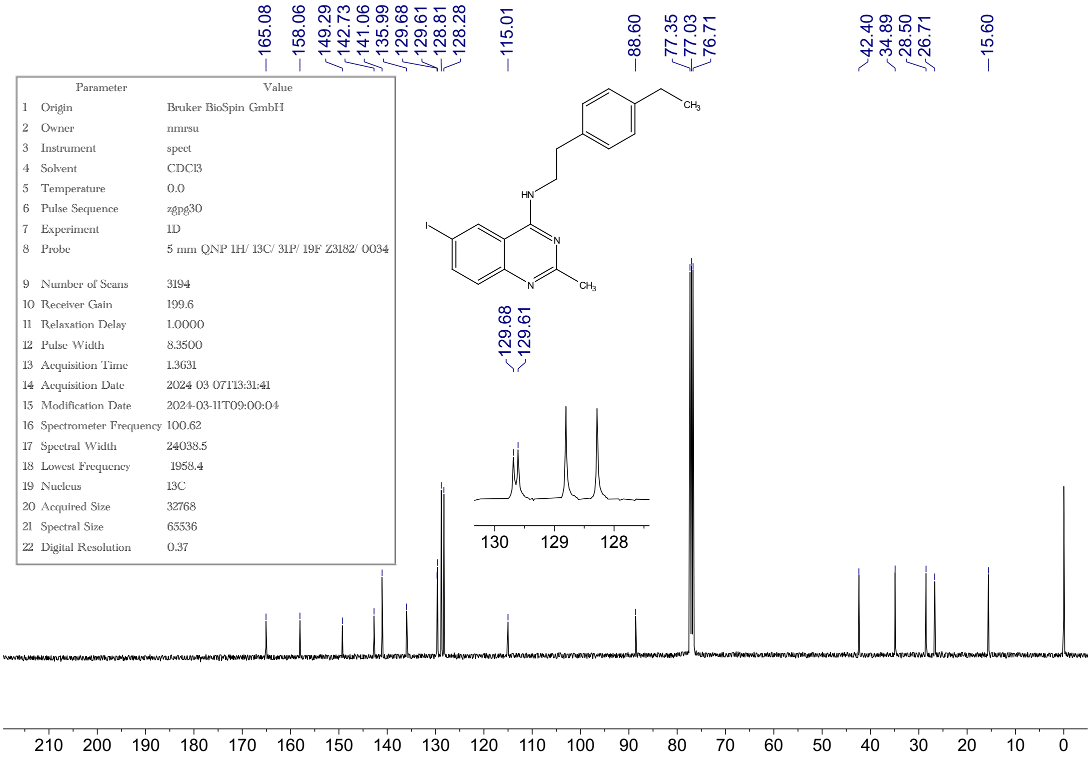

Figure S57. ^{13}C NMR spectrum (100 MHz) of compound **16d** in $CDCl_3$.

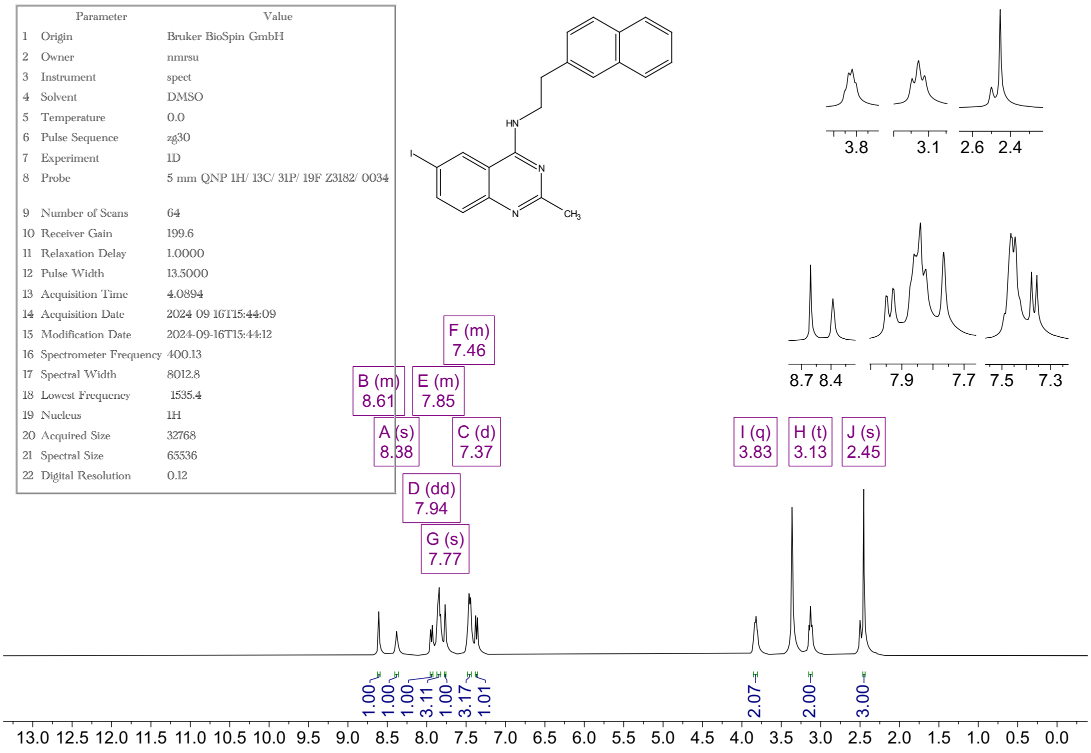

Figure S58. ^{1}H NMR spectrum (400 MHz) of compound **17d** in DMSO-d_6.

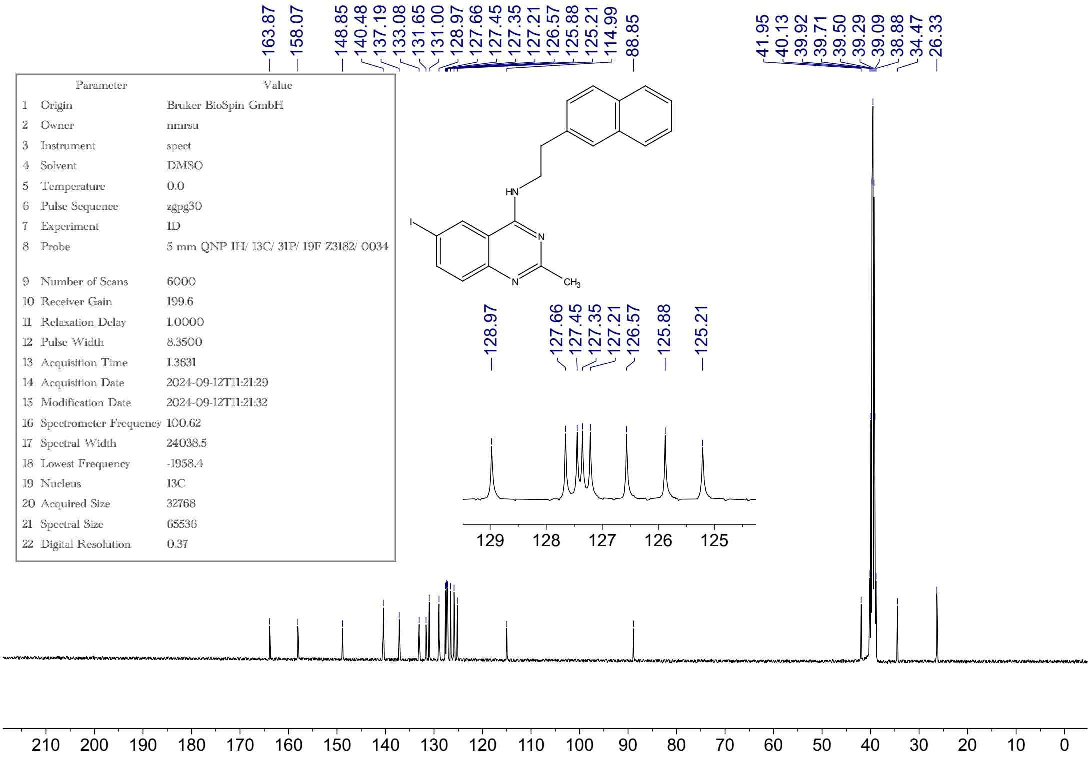

Figure S59. ^{13}C NMR spectrum (100 MHz) of compound **17d** in DMSO-d_6.

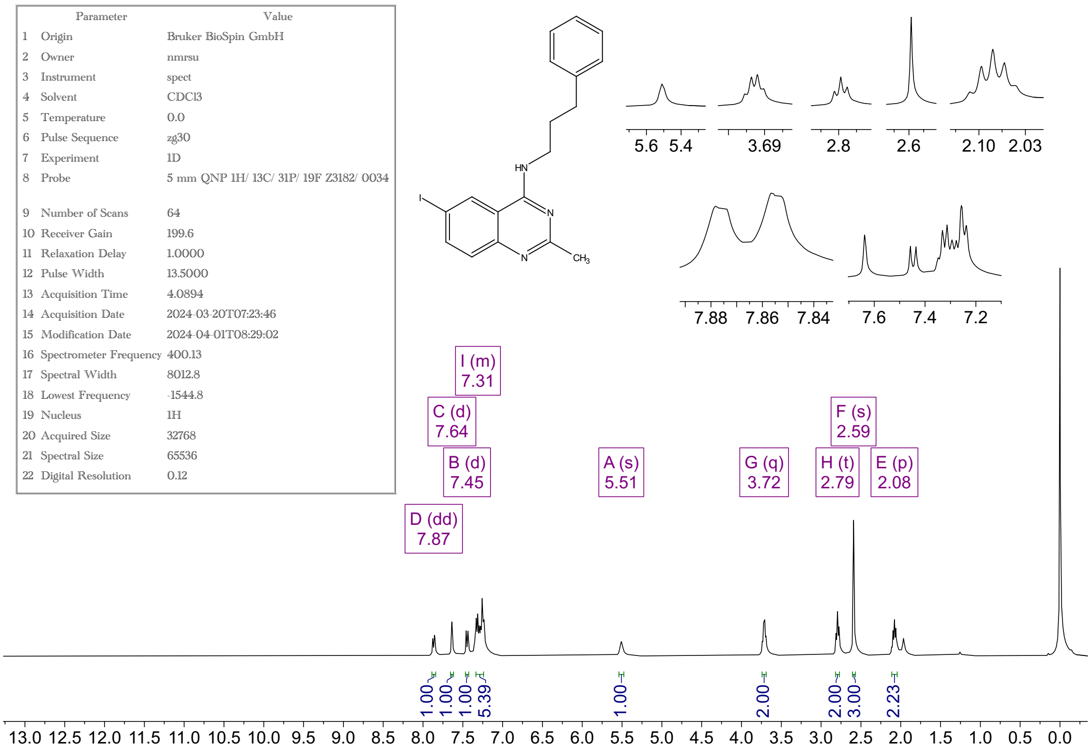

Figure S60. ^{1}H NMR spectrum (400 MHz) of compound **18d** in $CDCl_3$.

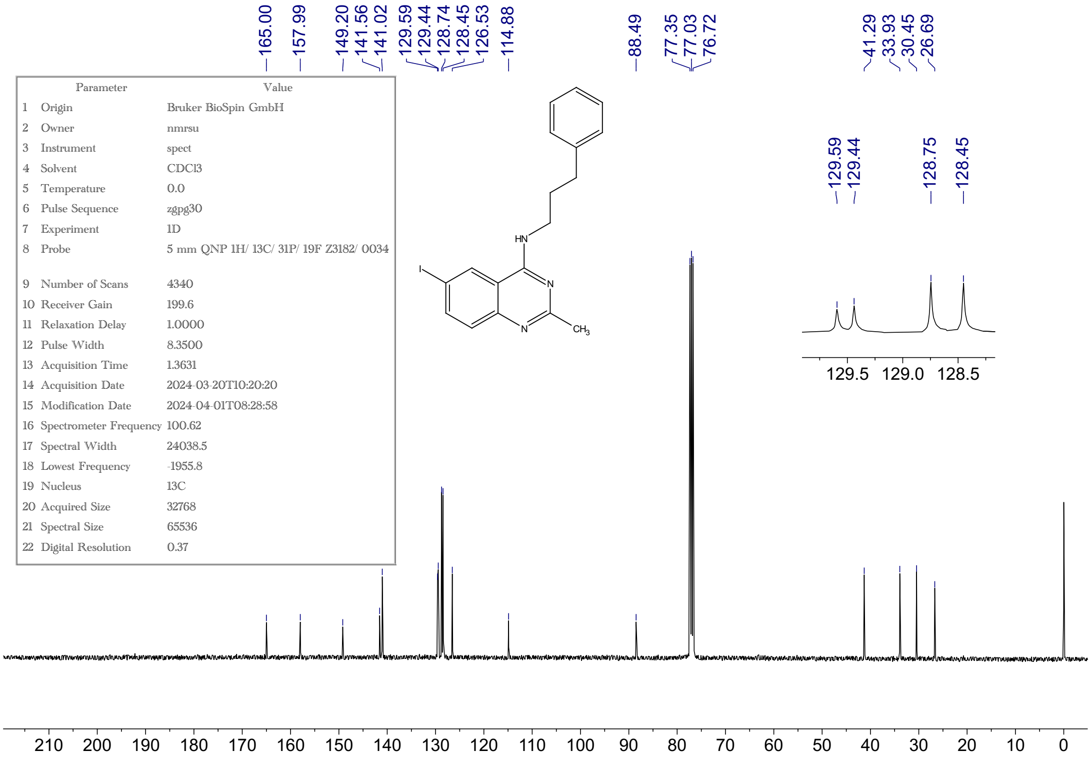

Figure S61. ^{13}C NMR spectrum (100 MHz) of compound **18d** in $CDCl_3$.

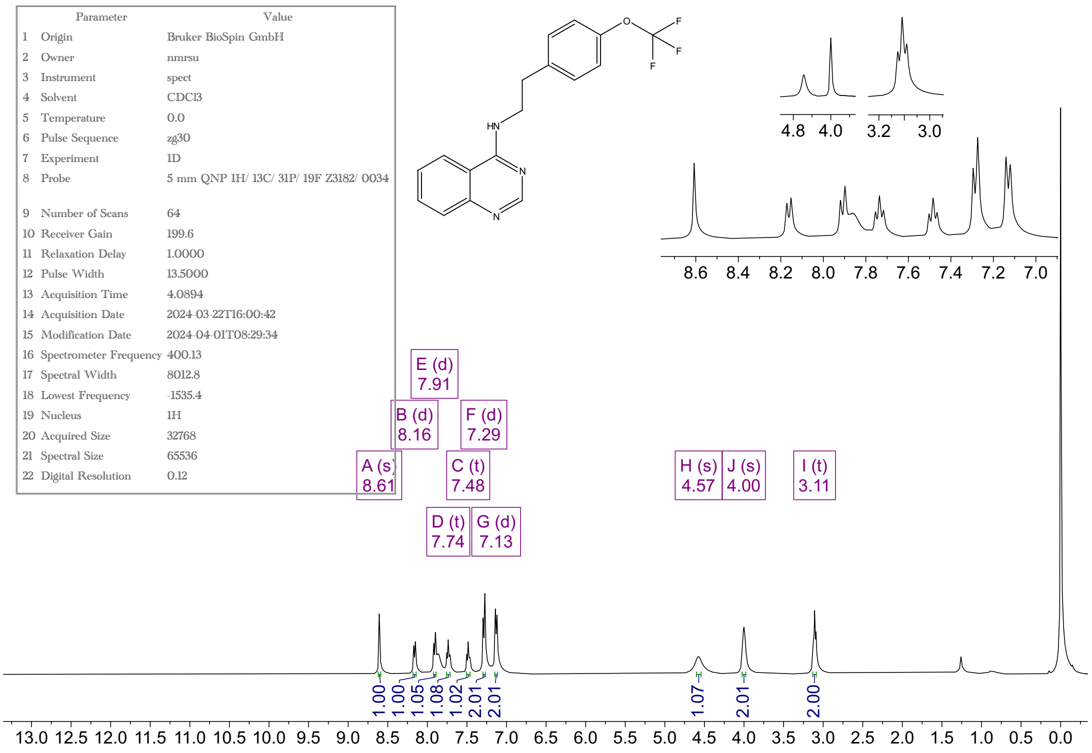

Figure S62. ^{1}H NMR spectrum (400 MHz) of compound **13e** in $CDCl_3$.

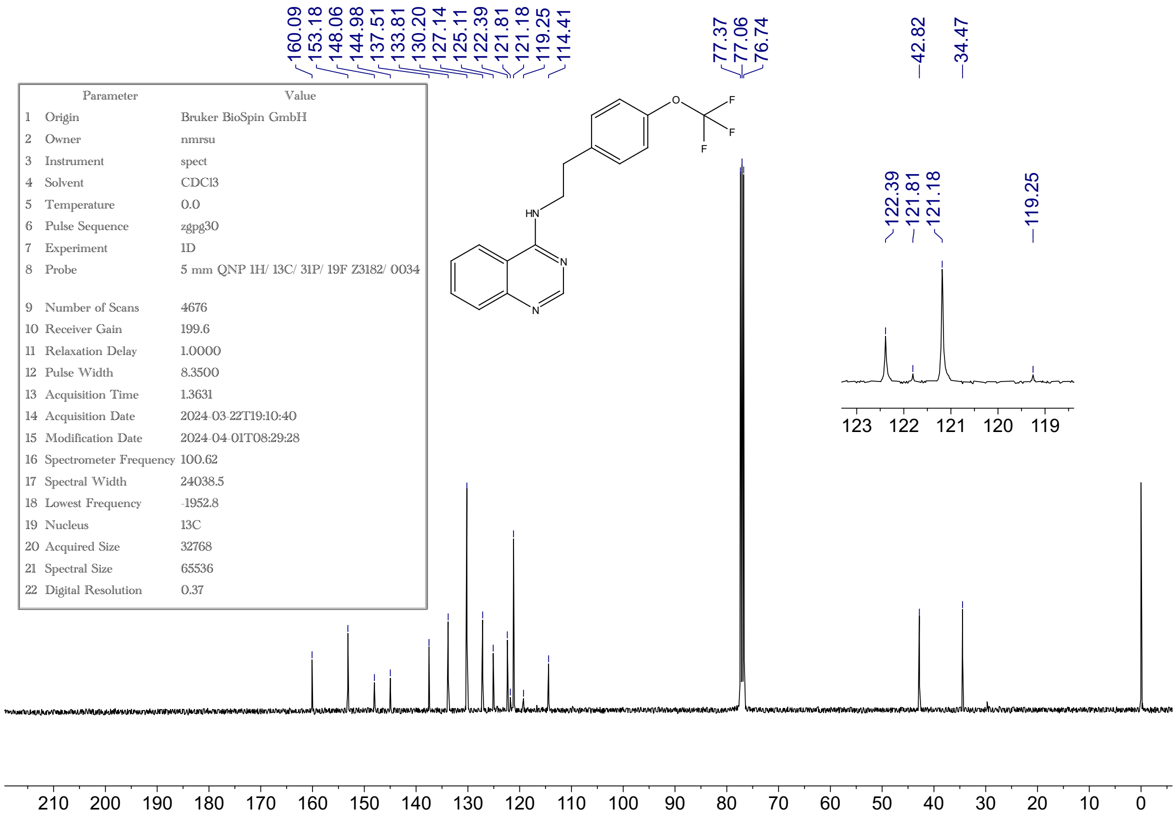

Figure S63. ^{13}C NMR spectrum (100 MHz) of compound **13e** in $CDCl_3$.

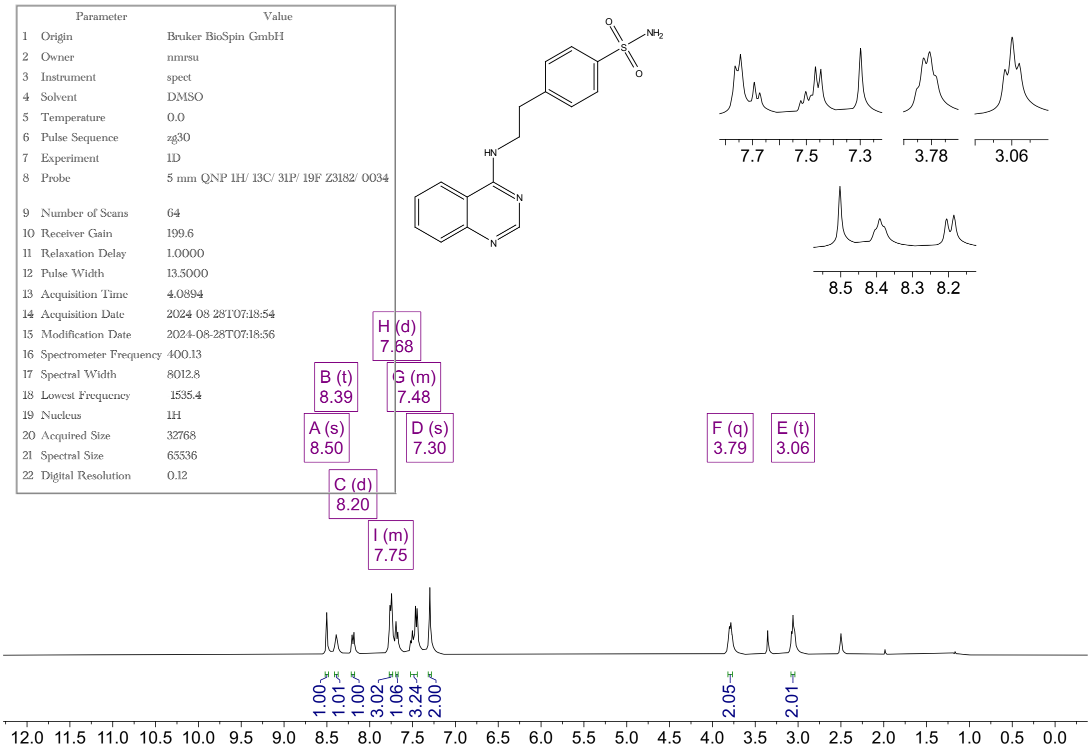

Figure S64. ^{1}H NMR spectrum (400 MHz) of compound **14e** in DMSO-d$_6$.

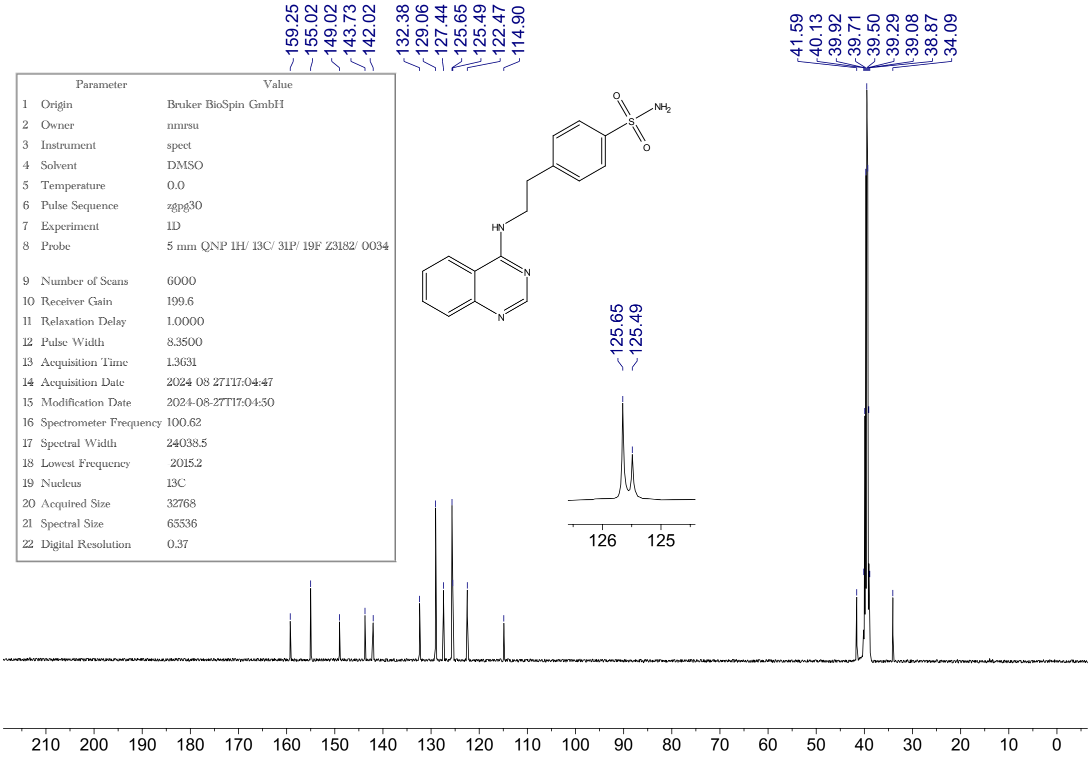

Figure S65. ^{13}C NMR spectrum (100 MHz) of compound **14e** in DMSO-d$_6$.

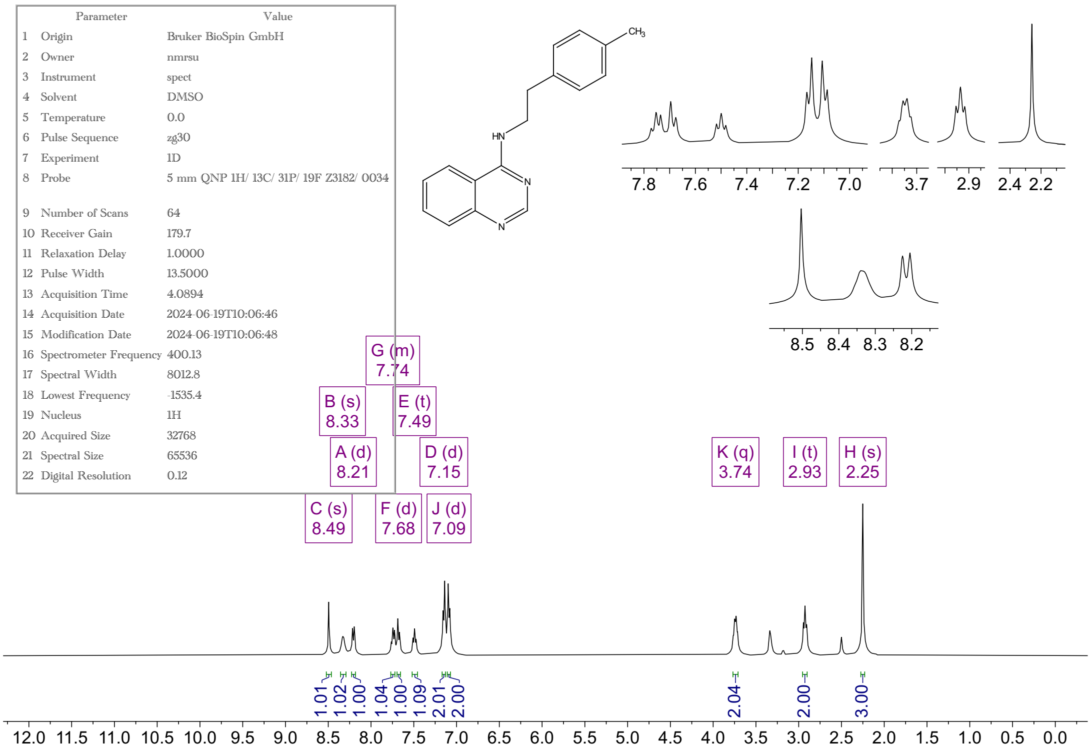

Figure S66. ^1H NMR spectrum (400 MHz) of compound **15e** in DMSO-d$_6$.

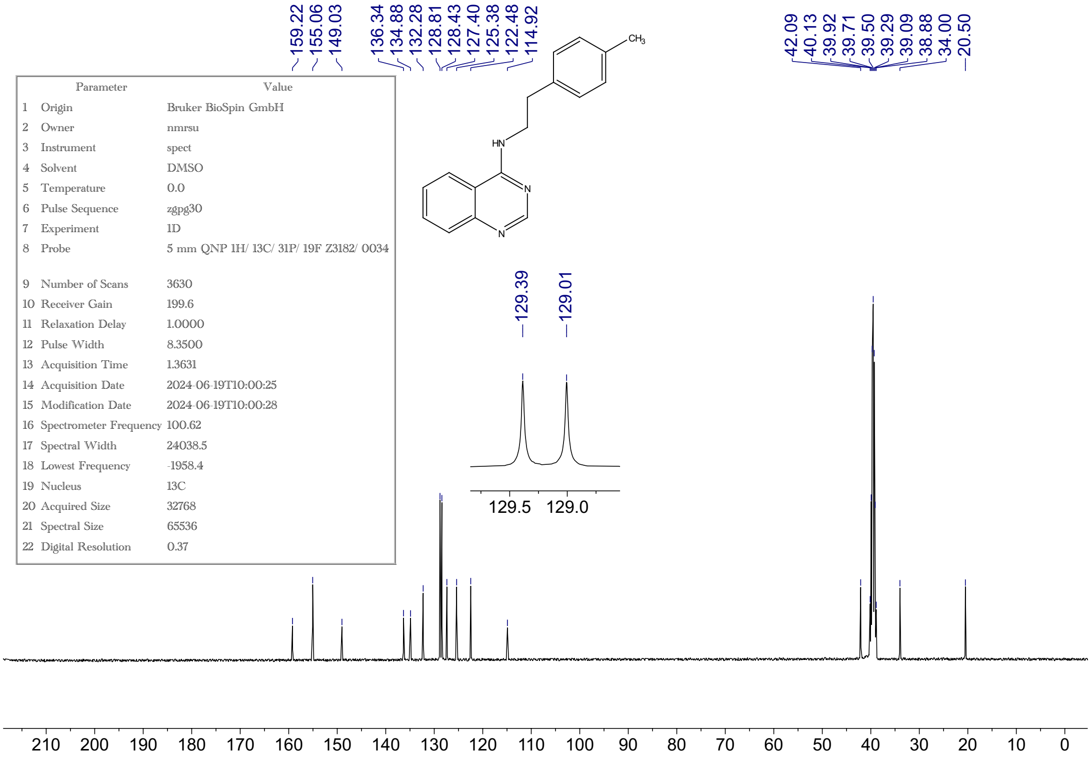

Figure S67. ^{13}C NMR spectrum (100 MHz) of compound **15e** in DMSO-d$_6$.

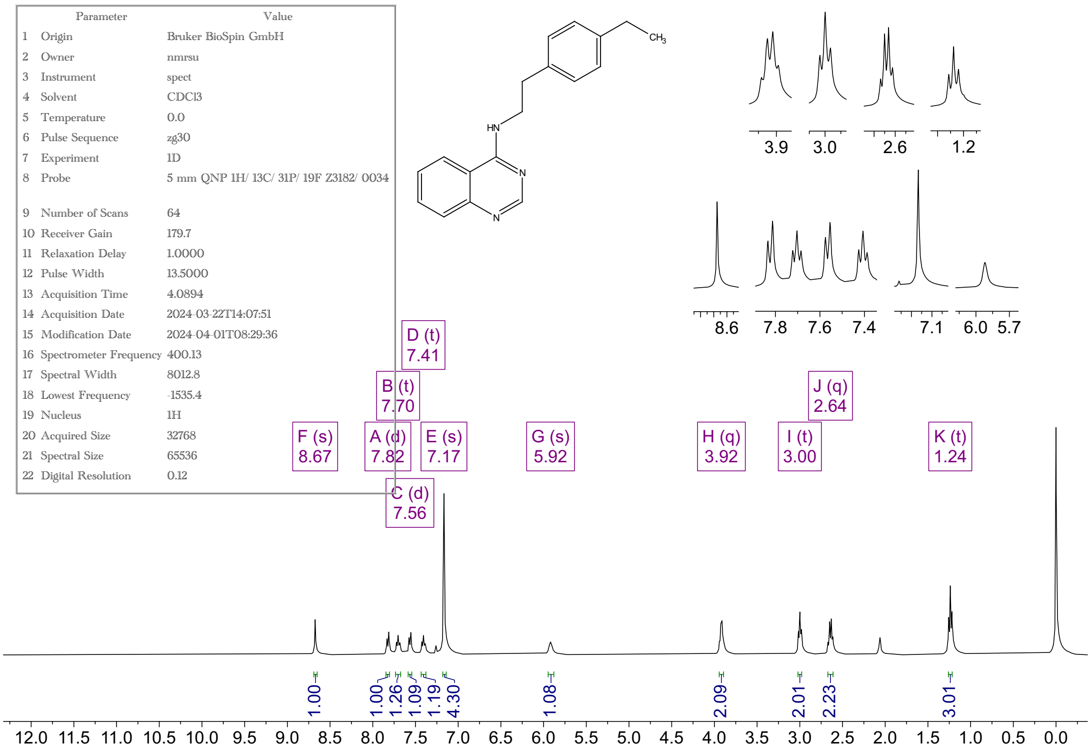

Figure S68. ^{1}H NMR spectrum (400 MHz) of compound **16e** in $CDCl_3$.

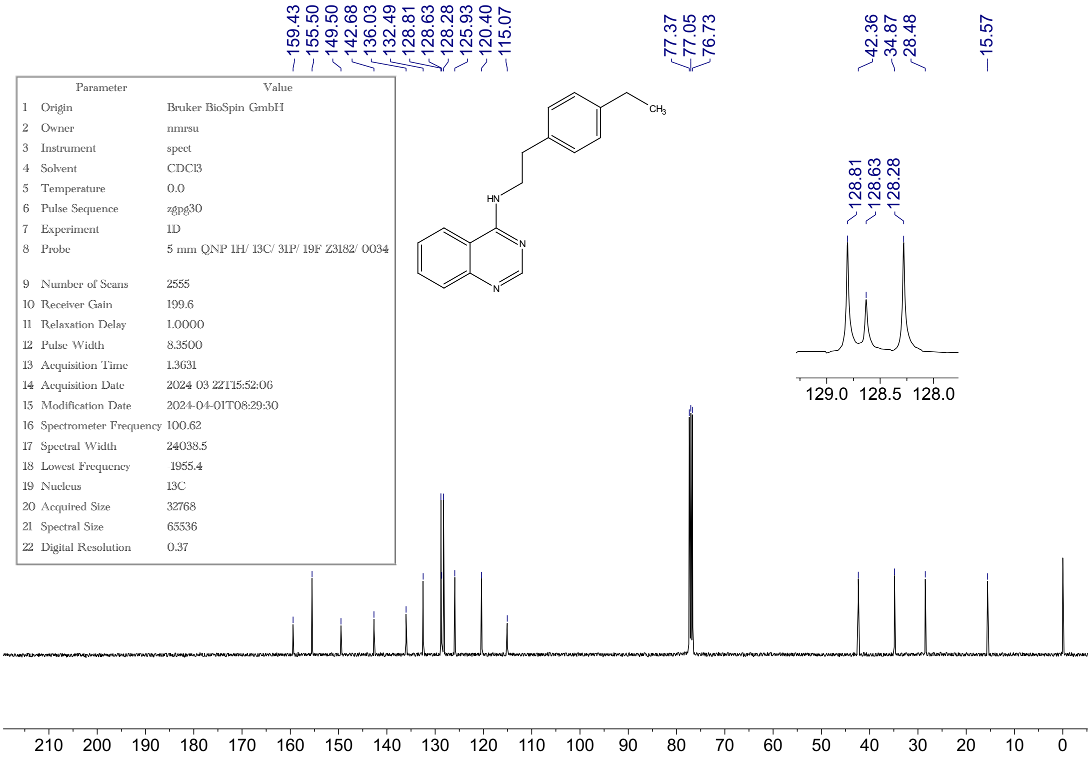

Figure S69. ^{13}C NMR spectrum (100 MHz) of compound **16e** in $CDCl_3$.

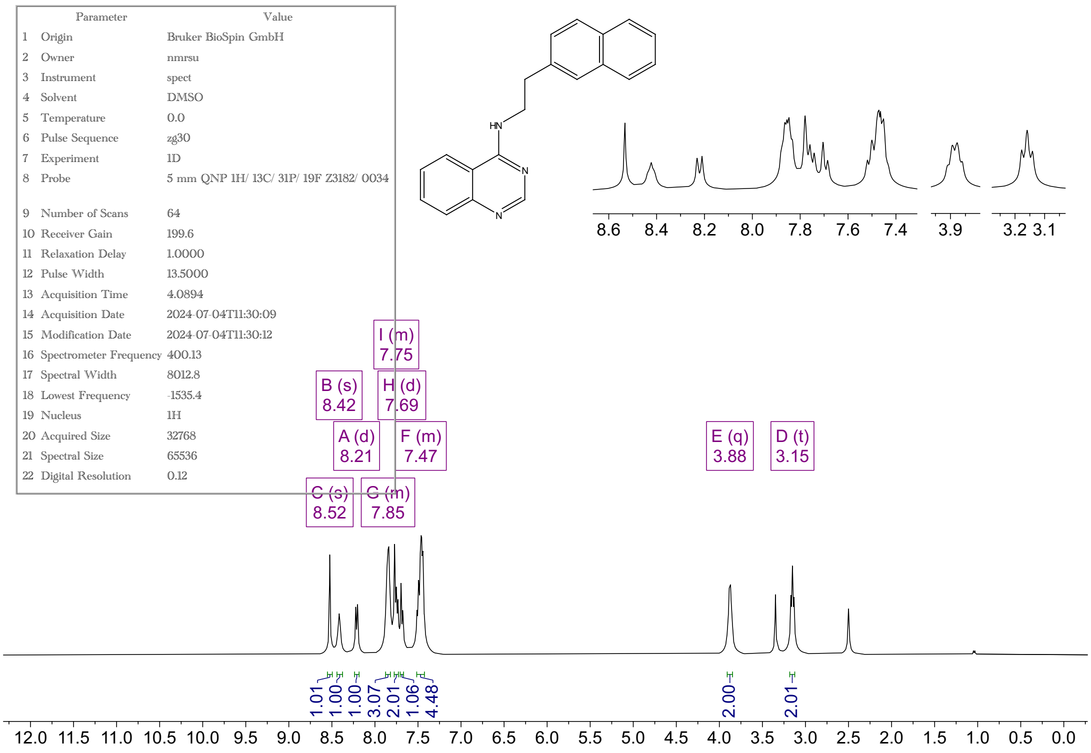

Figure S70. ^{1}H NMR spectrum (400 MHz) of compound **17e** in DMSO-d_6.

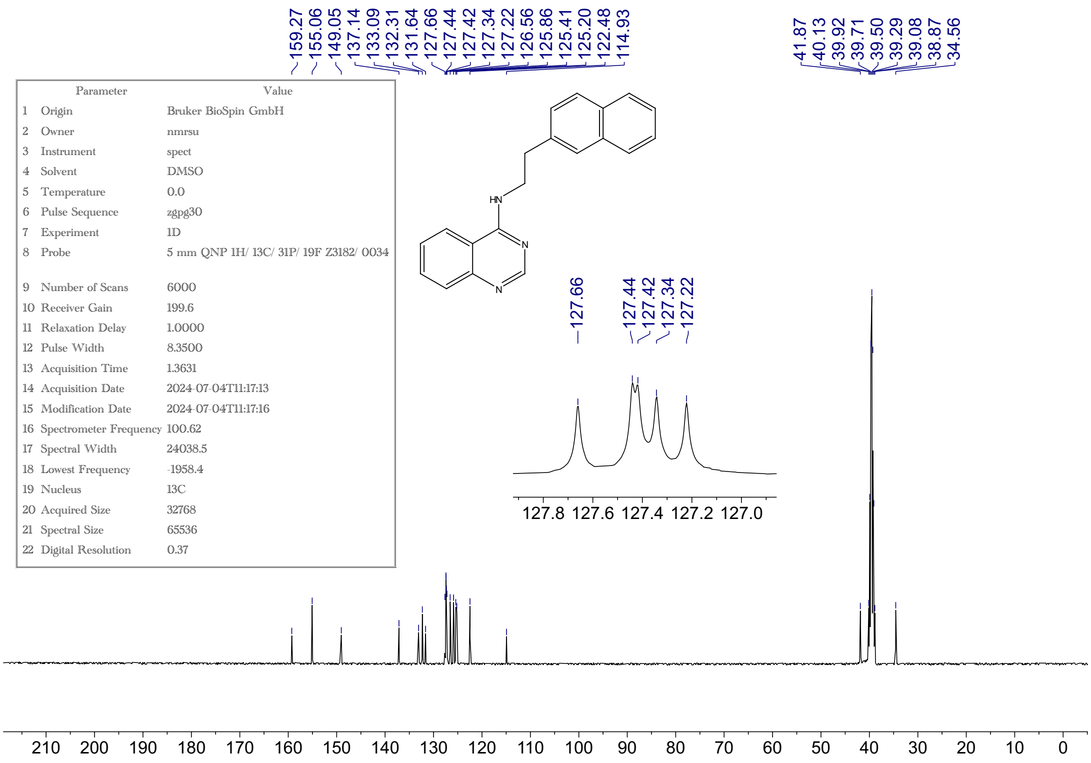

Figure S71. ^{13}C NMR spectrum (100 MHz) of compound **17e** in DMSO-d_6.

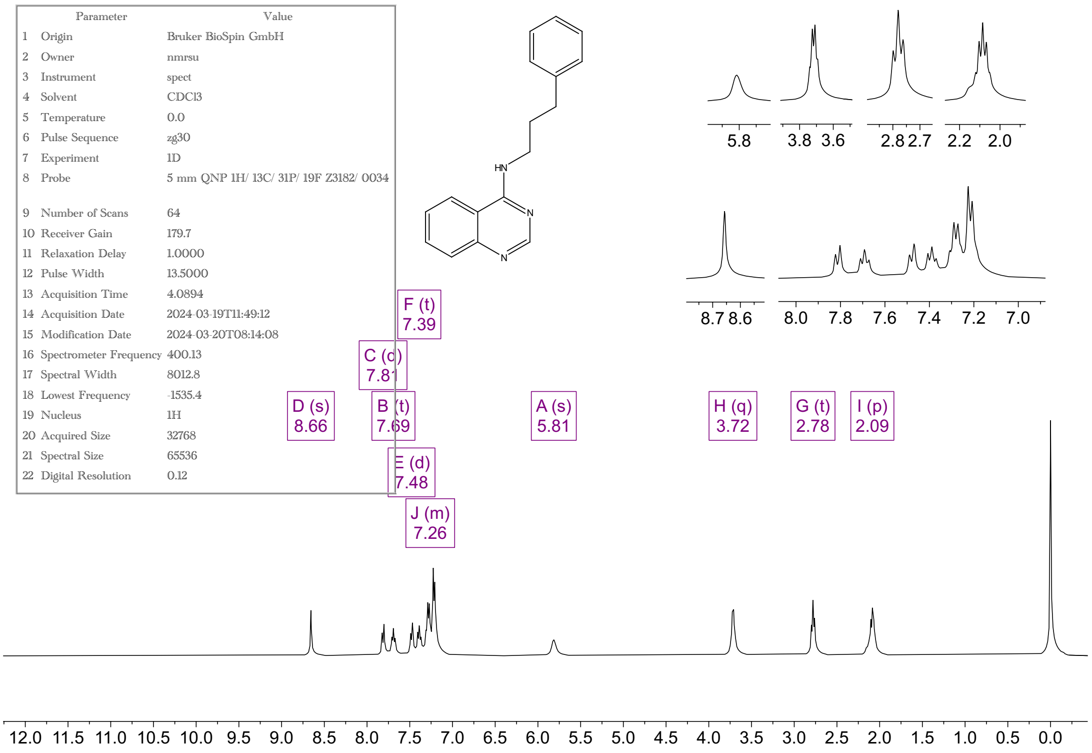

Figure S72. ^{1}H NMR spectrum (400 MHz) of compound **18e** in $CDCl_3$.

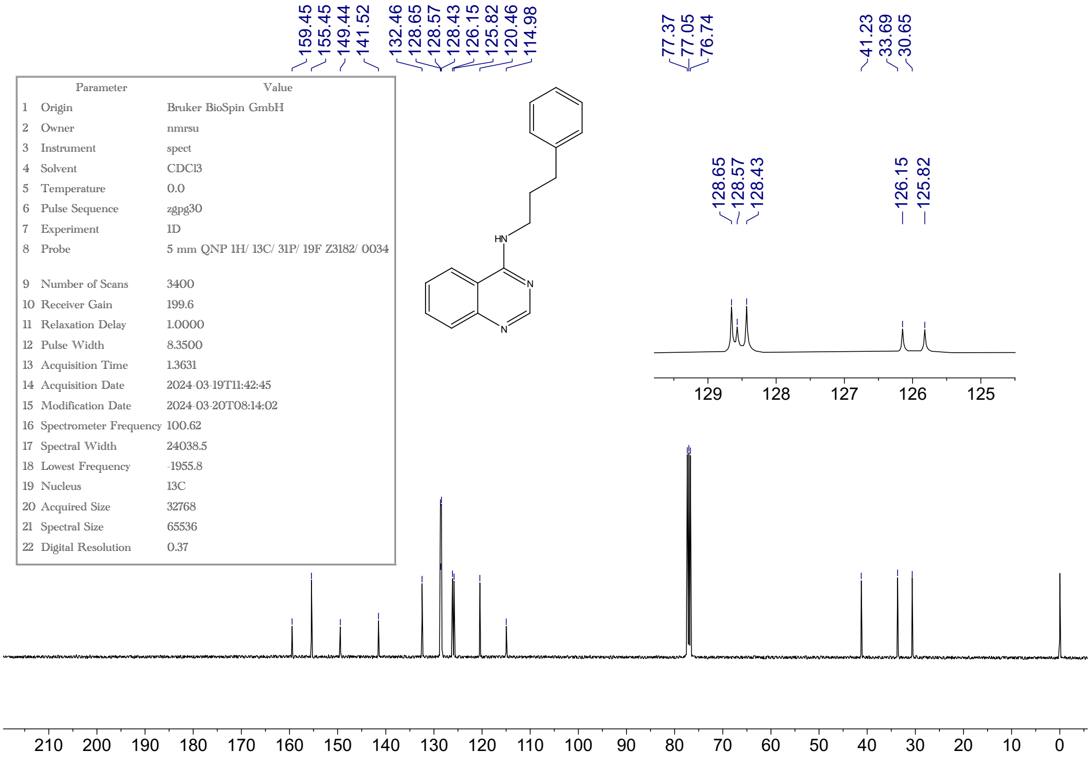

Figure S73. ^{13}C NMR spectrum (100 MHz) of compound **18e** in $CDCl_3$.

4. References

(1) Von Niementowski, St. Synthesen von Chinazolinverbindungen. *J. Für Prakt. Chem.* **1895**, *51* (1), 564–572. https://doi.org/10.1002/prac.18950510150.
(2) Arraché Gonçalves, G.; Castro Do Nascimento, F.; Moura E Silva, S.; Valim Bizarro, C.; Augusto Basso, L.; Machado, P. Synthesis of N-Phenethylquinazolin-4-Amines via Silylation-Amination Mediated by Hexamethyldisilazane. *Results Chem.* **2022**, *4*, 100539. https://doi.org/10.1016/j.rechem.2022.100539.
(3) Palomino, J.-C.; Martin, A.; Camacho, M.; Guerra, H.; Swings, J.; Portaels, F. Resazurin Microtiter Assay Plate: Simple and Inexpensive Method for Detection of Drug Resistance in *Mycobacterium Tuberculosis*. *Antimicrob. Agents Chemother.* **2002**, *46* (8), 2720–2722. https://doi.org/10.1128/AAC.46.8.2720-2722.2002.
(4) Carroll, P.; Muwanguzi-Karugaba, J.; Parish, T. Codon-Optimized DsRed Fluorescent Protein for Use in Mycobacterium Tuberculosis. *BMC Res. Notes* **2018**, *11* (1), 685. https://doi.org/10.1186/s13104-018-3798-3.
(5) Ollinger, J.; Bailey, M. A.; Moraski, G. C.; Casey, A.; Florio, S.; Alling, T.; Miller, M. J.; Parish, T. A Dual Read-Out Assay to Evaluate the Potency of Compounds Active against Mycobacterium Tuberculosis. *PLoS ONE* **2013**, *8* (4), e60531. https://doi.org/10.1371/journal.pone.0060531.
(6) Manning, A. J.; Ovechkina, Y.; McGillivray, A.; Flint, L.; Roberts, D. M.; Parish, T. A High Content Microscopy Assay to Determine Drug Activity against Intracellular Mycobacterium Tuberculosis. *Methods* **2017**, *127*, 3–11. https://doi.org/10.1016/j.ymeth.2017.03.022.
(7) Van Meerloo, J.; Kaspers, G. J. L.; Cloos, J. Cell Sensitivity Assays: The MTT Assay. In *Cancer Cell Culture*; Cree, I. A., Ed.; Methods in Molecular Biology; Humana Press: Totowa, NJ, 2011; Vol. 731, pp 237–245. https://doi.org/10.1007/978-1-61779-080-5_20.
(8) Repetto, G.; Del Peso, A.; Zurita, J. L. Neutral Red Uptake Assay for the Estimation of Cell Viability/Cytotoxicity. *Nat. Protoc.* **2008**, *3* (7), 1125–1131. https://doi.org/10.1038/nprot.2008.75.
(9) Marroquin, L. D.; Hynes, J.; Dykens, J. A.; Jamieson, J. D.; Will, Y. Circumventing the Crabtree Effect: Replacing Media Glucose with Galactose Increases Susceptibility of HepG2 Cells to Mitochondrial Toxicants. *Toxicol. Sci.* **2007**, *97* (2), 539–547. https://doi.org/10.1093/toxsci/kfm052.
(10) Dong, J.; Wang, N.-N.; Liu, K.-Y.; Zhu, M.-F.; Yun, Y.-H.; Zeng, W.-B.; Chen, A. F.; Cao, D.-S. ChemBCPP: A Freely Available Web Server for Calculating Commonly Used Physicochemical Properties. *Chemom. Intell. Lab. Syst.* **2017**, *171*, 65–73. https://doi.org/10.1016/j.chemolab.2017.10.006.
(11) BioByte Corp. *cLogp User Guide*. http://www.biobyte.com/bb/prod/40manual.pdf (accessed 2024-11-24).
